# Facilitators, barriers and acceptability of malaria reactive surveillance and response strategies in Vietnam: a mixed-methods study

**DOI:** 10.1136/bmjph-2024-000961

**Published:** 2024-12-16

**Authors:** Nguyen Xuan Thang, Win Han Oo, Win Htike, Ngo Thi Van Anh, Ngo Duc Thang, Dinh Son Ha, Bui Thi Luan, Vu Tuan Anh, Nguyen Ha Nam, May Chan Oo, Kaung Myat Thu, Kaung Myat Khant, Ei Phyu Htwe, Nilar Aye Tun, Katherine O'Flaherty, Paul A Agius, Freya J I Fowkes

**Affiliations:** 1 Department of Epidemiology, National Institute of Malariology Parasitology and Entomology, Hanoi, Vietnam; 2 Disease Elimination, Burnet Institute, Melbourne, Victoria, Australia; 3 Centre for Epidemiology and Biostatistics, Melbourne School of Population and Global Health, University of Melbourne, Melbourne, Victoria, Australia; 4 Health Security, Burnet Institute, Yangon, Myanmar; 5 Health Poverty Action, Hanoi, Vietnam; 6 Department of Epidemiology and Preventive Medicine, Monash University, Melbourne, Victoria, Australia; 7 Faculty of Health, Deakin University, Melbourne, Victoria, Australia

**Keywords:** Communicable Disease Control, Population Surveillance, Disease Notification, Disease Eradication, Community Health

## Abstract

**Introduction:**

Vietnam has achieved significant reductions in its malaria caseload over the past decades and is progressing towards malaria elimination. To achieve malaria elimination, the Vietnam Ministry of Health issued Guidelines for Malaria Surveillance and Prevention, a surveillance guide that describes malaria reactive surveillance and response (RASR) strategies—its implementation—is yet to be evaluated. Here, the facilitators, barriers and acceptability of the implementation of RASR strategies in Vietnam are explored and discussed thoroughly to provide recommendations for improvement of RASR strategies.

**Methods:**

A mixed-methods study was conducted in Binh Thuan and Phu Yen Provinces in Vietnam from November 2021 to April 2022 including quantitative surveys with health stakeholders and staff (n=36) and frontline health service providers (n=38), qualitative focus group discussions with frontline health service providers and mobile migrant populations (n=70) and semistructured in-depth interview with health stakeholders and staff (n=28). Quantitative and qualitative data were analysed descriptively and thematically.

**Results:**

Vietnam’s health system supports RASR strategies, and the RASR implementation data are well captured in the electronic communicable disease surveillance system of the Ministry of Health. Overall, RASR strategies are acceptable to both stakeholders and community members. However, successful implementation of RASR strategies is hindered by declining community interest in malaria elimination programme and limitations of infrastructure, budget, human resources, and terrain difficulties.

**Conclusions:**

Overall health system support and policy commitment are key to successful implementation of RASR strategies and therefore for achieving malaria elimination. Despite high-performance and well-accepted RASR strategies, more financial and human resource investments are warranted to investigate and respond to all malaria foci in time. Village health workers could be used effectively to engage community members and mobile migrant populations in RASR activities.

**WHAT IS ALREADY KNOWN ON THIS TOPIC**

- Vietnam adopted 2-7 reactive surveillance and response (RASR) strategies, and its performance in terms of completeness and timeliness is high. However, its facilitators and barriers and acceptability are yet to be explored.

**WHAT THIS STUDY ADDS**

- Overall, implementation of RASR strategies in Vietnam is well accepted by the community and stakeholders. To further improve performance of RASR strategies, better community engagement and investments in the RASR activities are required.

**HOW THIS STUDY MIGHT AFFECT RESEARCH, PRACTICE OR POLICY**

- This study identifies how RASR strategies can be best used to progress malaria elimination and will have policy impact in Vietnam and broadly in Greater Mekong Subregion.

## Introduction

 Vietnam has achieved remarkable progress in malaria caseload reduction.^1^ From 2000 to 2021, malaria cases decreased by 99.4%,^2^ and in 2022, there were only 455 confirmed malaria cases, 90 of these being classified as imported, and zero deaths were reported.^3^ Vietnam now aims to certify 55 out of 63 provinces as malaria-free by 2025 and is making significant progress towards the national and Greater Mekong Subregion’s goal of malaria elimination by 2030.^4^

To achieve these goals, Vietnam is implementing a 5-year (2021–2025) national strategy for malaria elimination which focuses on reducing malaria morbidity and mortality through early diagnosis and effective treatment.^5^ The success of this strategy depends on the effective implementation of the nationwide malaria surveillance and response system. The National Institute of Malariology, Parasitology and Entomology (NIMPE) is enhancing surveillance as a core intervention that encompasses disease tracking, and taking action in response to the index malaria case and associated programmatic responses.^6^ In 2016, the international Reactive surveillance and Response (RASR) approaches of proven effectiveness,^7^,^9^ namely the 1-3-7 strategy adopted by the WHO and other international organisations, were adapted to the national context.

The Vietnam RASR strategy includes malaria case notification by frontline health service providers (FHSP) to respective focal persons in district and provincial health departments via paper-based reporting, direct phone calling or the web-based electronic Communicable Disease Surveillance System – Malaria Management System (eCDS-MMS). When a malaria case is notified, responsible persons from the district health department investigate the case using the case investigation form and classify the case (local, imported, introduced or relapse cases) supervised by provincial health department staff. Afterwards, the index case household and surrounding area are investigated by district and provincial health department staff for the presence of malaria foci which includes interviewing household members, reactive case detection (RACD), including diagnosis and treatment of detected secondary malaria cases, and entomological surveillance and responses, such as larval source management. The detected foci are also classified as active, residual non-active or clear foci and responded to accordingly.^5 10 11^

In March 2016, the Vietnam Ministry of Health ordered Decision 741 that all suspected malaria cases must be confirmed with microscopy, reported within two and investigated within 3 days of diagnosis. Additionally, focus investigation and responses including RACD must be done among 20–30 neighbouring households of the index household as early as possible after the case investigation.^10^ In October 2021, the process of carrying out the RASR strategy was modified as per the new Decision 4922, in which either rapid diagnostic test, microscopy or PCR is allowed for confirmation of a malaria case. Case notification and investigation must be completed within 2 days from diagnosis, followed by the focus investigation and responses within 7 days of a malaria case diagnosis.^11^ Monitoring of the implementation of the RASR strategy is essential to identifying areas for improvement of its overall effectiveness. Recently, the performance and feasibility of the RASR strategy were assessed and found to be high (≥ 79% completeness and timeliness of case and focus investigation).^12^ However, the acceptability of and facilitators and barriers to its implementation have yet to be explored. This paper thoroughly investigates the facilitators, barriers and acceptability of RASR strategies and its implementation in Vietnam, thereby providing recommendations for the improvement of RASR strategies with the ultimate aim of contributing to malaria elimination goals of Vietnam and more broadly in the Greater Mekong Subregion.

## Materials and methods

### Study design, setting and populations

In Vietnam, at the subnational level, RASR activities are carried out by the staff of the Centre for Disease Control unit of the respective provinces and cities, district health centres and commune health centres. They are supervised and supported by central level staff from Department of Preventive Medicine, Ministry of Health, NIMPE, Institute of Malariology, Parasitology and Entomology (Quy Nhon) and Institute of Malariology, Parasitology and Entomology (Ho Chi Minh City).

The study included primary data collection of quantitative surveys and qualitative focus group discussions and semistructured in-depth interviews. Opinions and views on the facilitators and barriers and acceptability regarding steps of RASR that include case notification, case investigation and classification, and focus investigation and responses were explored. Findings from surveys, focus group discussion and in-depth interviews regarding the performance and feasibility of RASR have previously been reported.^12^ The study also explored development of and participants’ experiences in management of RASR activities. The reporting of the study adhered to the Strengthening the Reporting of Observational studies in Epidemiology checklists^13^ (online supplemental additional 1).

The mixed-methods primary data collections were conducted in Phu Yen and Binh Thuan Provinces in Vietnam from November 2021 to April 2022. Phu Yen and Binh Thuan Provinces were chosen as these provinces have been implementing malaria elimination activities including RASR strategies with international funding support, presence of participants who could provide valuable information of RASR strategies and operational and budgetary feasibility.

Surveys were administered to health stakeholders and staff responsible for managing or supervising RASR (n=36), as well as FHSP (n=38) using Questionnaires 1 and 2, respectively (online supplemental additional 2). The majority of health stakeholders and malaria programme staff surveyed were male and held roles in malaria diagnosis, prevention and control, research, and surveillance or management, while the majority of participants in the FHSP survey were female clinical service providers such as medical doctors, nurses, midwives, health centre staff and village health workers (VHWs) (online supplemental table 1). Two-stage sampling was applied by first selecting the health facilities implementing RASR from March 2016 as per Decision 741 in endemic areas in the two selected provinces, followed by purposive selection of participants who have experience in malaria control and elimination in the selected health facilities. Given the purposive selection of all available participants in the selected health facilities and intended descriptive analysis of the survey data, no formal sample size calculation was done.

Qualitative consultations with health stakeholders, staff and key experts at national and provincial levels (n=28) in Phu Yen and Binh Thuan provinces were done by conducting in-depth interviews. Focus group discussions with FHSPs at district (Dong Xuan and Bac Binh districts) and commune levels (n=34) and in the community with forest-going mobile and migrant populations (MMPs) (n=36) (online supplemental additional 4) in groups of four to five people were also conducted (online supplemental table 2). MMPs were community members who often go to field or forest sites or who travel to another country or province. MMPs who reported being diagnosed with malaria at least once were selected by commune health centre staff from villages that had malaria cases. Purposive sampling with recruitment criteria predetermined by NIMPE researchers was used for recruiting stakeholders/staff and MMPs. The recruitment criteria included locality, designated role in managing malaria RASR and representation of the organisation. The phenomenological approach was applied to qualitative consultations.

### Data collection, management and analysis

Both quantitative and qualitative data collections were conducted confidentially in private locations and in the primary language of the participants, Vietnamese, in all cases. NIMPE staff (epidemiology unit staff) interviewed the study participants face-to-face using printed questionnaires and topic guides during surveys, focus group discussions (four staff facilitators, one coordinator and one note taker) and in-depth interviews (two staff facilitators, one note taker) which were held for approximately 45, 60 and 90 min, respectively.

The quantitative survey data were entered into the Excel data spreadsheet and imported into Stata V.16.1 for data cleaning, management and analysis (online supplemental file 5). Quantitative descriptive analyses were performed to understand potential barriers and enablers in each step of RASR. The focus group discussions and in-depth interviews were audio-recorded with the consent of the participants. Audio recordings were transcribed verbatim in Vietnamese and translated into English alongside the field notes by two researchers who also took part in qualitative data collection and note taking. They were then organised, managed and analysed in NVivo V.12. Thematic (deductive followed by inductive) analysis was undertaken using the qualitative descriptive approach.^14^ A deductive thematic framework that includes coding definitions, themes and subthemes were developed before the analysis. Two researchers immersed the data by reading the transcripts three times. An inter-coder reliability test was done by coding a selected transcript by two researchers independently and cross-checking the coded transcripts for agreement. Each of the two researchers then coded data separately referring the deductive thematic framework. After coding, the researchers then discussed themes and subthemes to reach a consensus on the final thematic framework and interpretation.^15^ Facilitators and barriers to and acceptability of implementing RASR strategies in Vietnam were reported thematically.

### Ethical considerations

The study protocol (online supplemental file 6) has been reviewed and approved by the Alfred Ethics Review Committee, Australia (Approval Number - 393/21) and the Ethical Review Board, NIMPE Vietnam (Approval Number – 32/HDDD). Written informed consent was obtained from all study participants before data collection.

### Patient and public involvement

In this study, the members of the public such as health stakeholders, staff and key experts as well as FHSPs were involved in the design, conduction and dissemination of the research. Before data collection, preliminary consultations were done with participants for selection of study areas, sampling strategy and data collection procedure and logistics. During data collection, participants assisted in recruitment and logistics of the data collection. Stakeholders and staff from the two provinces attended the dissemination meeting organised in Hanoi, Vietnam, in February 2023 and provided critical feedback.

## Results

### Facilitators

Respondents reported that commitment by all stakeholders and having the national policy support for malaria elimination and RASR strategies as a facilitator of the successful implementation of RASR in Vietnam. RASR is integrated in the national health system. Health staff and facilities including private health facilities at the community to the central level are committed and contribute to malaria elimination and RASR activities.

In focus group discussions, FHSPs identified that the timely case notification of the index case, thorough case investigation and comprehensive execution of RACD was a facilitator to proactively and promptly detect secondary malaria cases. In addition, in qualitative consultations, FHSPs and stakeholders mentioned that integrating RASR data into national electronic reporting system of eCDS-MMS significantly enhanced timely notification, investigation and responses in the RASR strategy compared with the traditional paper-based notification and reporting.

Another important factor identified as a facilitator to the successful execution of case and focus investigations was availability of correct travel and movement information from the malaria cases and surrounding community members. MMPs mentioned that there is no reason to hide their travel history including visiting the forest. They are willing to cooperate with case and focus investigations led by FHSPs.

*There is no challenge (to declare the forest visits). If a doctor comes to investigate, I will cooperate. If a doctor instructs, I will follow* (MMP, Phu Yen Province)

Minimal or no language barrier between FHSPs and community members was also identified as a facilitator to the successful implementation of RASR.

*About languages, all ethnic people here speak Kinh, so there is no problem about language.* (FHSP, Binh Thuan Province)

### Barriers

The majority of survey respondents mentioned that there was no barrier for timely case notification (40/73, 54.8%), case investigation (49/73, 67.1%) or focus investigation (52/72, 72.2%) (table 1), which was supported by qualitative findings.

**Table 1:** Barriers to implementation and adherence to current reactive surveillance and response strategies (survey)

Barriers	Health stakeholders/staff	FHSPs	Total
Barriers to conducting case notification within 1 day,*† n (%)			
No barrier	17 (48.6)	23 (60.5)	40 (54.8)
Poor phone connection/difficult reporting	0 (0.0)	4 (10.5)	4 (5.5)
Difficult or unable to contact/find patient	1 (2.9)	2 (5.3)	3 (4.1)
Insufficient time	3 (8.6)	5 (13.2)	8 (11.0)
Insufficient manpower	0 (0.0)	1 (2.6)	1 (1.4)
Weather and transportation difficulties	2 (5.7)	0 (0.0)	2 (2.7)
Unspecified barriers	13 (37.1)	5 (13.2)	18 (24.7)
Barriers to conducting case investigation within 3 days,*† n (%)			
No barrier	20 (57.1)	29 (76.3)	49 (67.1)
Difficult or unable to contact/find patient	0 (0.0)	2 (5.3)	2 (2.7)
Insufficient time	1 (2.9)	4 (10.5)	5 (6.8)
Weather and transportation difficulties	0 (0.0)	2 (5.3)	2 (2.7)
Unspecified barriers	14 (40.0)	3 (7.9)	17 (23.3)
Barriers to conducting focus investigation and response within 7 days,*† n (%)			
No barrier	22 (62.9)	30 (81.1)	52 (72.2)
Difficult or unable to contact/find patient	1 (2.9)	2 (5.4)	3 (4.2)
Insufficient time	1 (2.9)	3 (8.1)	4 (5.6)
Insufficient manpower	1 (2.9)	0 (0.0)	1 (1.4)
Unspecified barriers	10 (28.6)	2 (5.4)	12 (16.7)
Barriers to following current guidelines for implementation of RASR activities,*† n (%)			
No barrier	5 (15.6)	22 (59.5)	27 (39.1)
Language barrier	0 (0.0)	1 (2.7)	1 (1.4)
Poor cooperation from patient and lack of awareness	4 (12.5)	10 (27.0)	14 (20.3)
Difficult or unable to contact/find patient	1 (3.1)	2 (5.4)	3 (4.3)
Insufficient time	1 (3.1)	0 (0.0)	1 (1.4)
Insufficient funding	11 (34.4)	1 (2.7)	12 (17.4)
Insufficient manpower	10 (31.2)	0 (0.0)	10 (14.5)
Unspecified barriers	6 (18.8)	1 (2.7)	7 (10.1)
COVID-19 pandemic had impact on successful implementation of RASR strategies, n (%)			
Yes	29 (80.6)	29 (76.3)	58 (78.4)
No	7 (19.4)	9 (23.7)	16 (21.6)
Impact of COVID-19 pandemic on implementation of RASR strategies,† n (%)			
Unspecified impacts	16 (44.4)	9 (23.7)	25 (33.8)
Travel restriction, social distancing and isolation reduces malaria	10 (27.8)	7 (18.4)	17 (23.0)
Increased awareness on infectious diseases including malaria	1 (2.8)	1 (2.6)	2 (2.7)
Travel restriction and shortage of human resource reduces programme activities	3 (8.3)	7 (18.4)	10 (13.5)
Poor cooperation from community due to fear of COVID-19	2 (5.6)	5 (13.2)	7 (9.5)
Suggestion to improve current RASR activities,* n (%)			
No suggestion	-na-	13 (36.1)	13 (36.1)
Providing information, education and communication materials, preventive measures and financial support to community	-na-	13 (36.1)	13 (36.1)
Providing training to FHSPs	-na-	2 (5.6)	2 (5.6)
Providing financial and other supportive materials to FHSPs	-na-	6 (16.7)	6 (16.7)
Others	-na-	2 (5.6)	2 (5.6)

* Missing values present.

† Multiple responses allowed.

FHSPsfrontline health service providersRASRreactive surveillance and response

*In fact, there is no issue overall. We can do it (RASR activities).* (FHSP, Phu Yen Province)

Following unspecified barriers, insufficient time, manpower and weather and transportation difficulties were reported as barriers to conducting key RASR activities within the specified timeframes (table 1) and as overall challenges to conducting case investigation (online supplemental table 3). Although 27/69 (39.1%) of respondents mentioned there was no barrier to follow current guidelines for implementation of RASR activities, 14/69 (20.3%) of respondents reported lack of awareness about malaria elimination and poor cooperation from malaria cases, and 12/69 (17.4%) reported insufficient funding as barriers. For completion of case investigation and RACD in the community, difficulty in contacting (or locating) malaria cases was the most frequently reported challenge (28/70, 40.0% and 23/61, 37.7%), followed by uncooperative patients and insufficient information from patients (13/70, 18.6% and 12/61, 19.7%) and terrain difficulty (10/70, 14.3% and 8/61, 13.1%) (online supplemental table 3).

Decreased community interest and participation were identified as a barrier to the successful implementation of RASR. Malaria is no longer a public health threat in many areas in Vietnam following significant declines in cases and malaria associated mortality over the past two decades. As such, the community interest on malaria and its interventions including RASR activities also declined, and it was noted that local people were not interested to get tested for malaria if they were not experiencing febrile illness.

*In general, the people don’t pay any more attention about malaria because malaria is not prevalent as before.* (FHSP, Binh Thuan Province)

*Because of the customs and traditions of some people, they don’t agree to take the blood smear test if they don’t get fever.* (FHSP, Binh Thuan Province)

According to FHSPs, some community members were not willing to participate in case investigation and RACD due to the additional time commitment required. Nevertheless, community members including MMPs said that they cooperated RASR activities such as providing information in case and focus investigations and getting tested for malaria in RACD if initiated by FHSPs.

In qualitative consultations, FHSPs and stakeholders identified limitations in infrastructure, medicines and commodities, budget, and human resources as barriers to RASR implementation in the field. Shortages of medicines and commodities such as blood sample collection materials limited the timely and complete execution of all RASR activities. In focus group discussions, FHSPs reported that an uninterrupted supply of all required materials would be required if they are to implement the RASR strategy completely and in a timely manner. Furthermore, available budget for implementation of RASR was reported as limited and often reliant on external funding sources.

*To be honest, there is no budget from the National Program for taking blood smears. There is no budget for insecticide spraying; still owing salaries for spray men. They must meet the mosquito spraying target. But there is no budget for implementation of spraying. If there is no more supported project [international funding], it will be very difficult to continue the activities.* (FHSP, Binh Thuan Province)

*I am talking about the investigation of a malaria case. In recent years, it is supported by Regional Artemisinin Initiative [an international multilateral donor funded project]. In fact, the fund from national malaria program alone is not enough to implement RASR. With the support of project, it is doing well. Honestly, if any cases found outside of the project communes, I do not know how to handle it. Fortunately, all cases so far are in project communes.* (A stakeholder, Binh Thuan Province)

More financial investment was requested to maintain engagement in RASR among VHWs and for FHSPs to execute RASR activities effectively. FHSPs requested finances for mobile phone communication with the VHWs and patients, as well as motorbikes or electric bikes to travel to the patient’s residence for case and focus investigations. Similarly, community members including MMPs requested incentives and commodities such as cash, tonics (such as vitamins, nutritional supplements or herbal medicines) or mosquito repellent for them to fully cooperate in the RASR activities.

*People want tonics or mosquito repellent creams in return for blood sampling. In the past, there was a team that often come to investigate malaria hotspots and the person who took blood smears paid 10,000 VND per suspected case. From then on, people have compared the support provided between teams. This is also one of our difficulties [to convince the community] while the investigation is conducted*. (VHW, Binh Thuan Province)

It was also noted that in peripheral field offices, FHSP human resources for implementation of RASR were limited, and one staff member was often responsible for the implementation of many programmes.

*There is an issue about human resources. Only one person [in a field office] cannot handle all the tasks; but we are trying our best. In fact, each department has few staff but there are so many programs. we could not meet demands from all the programs.* (FHSP, Binh Thuan Province)

In focus group discussions, FHSPs reported that availability of electricity and internet access was intermittent in some rural areas but crucial for timely reporting of malaria cases especially through eCDS-MMS and therefore for the overall RASR strategy. Even if the internet connection is available in the villages, malaria cases detected outside of the village especially in the forest areas could not be reported in time. Further, when it comes to the case and focus investigations, terrain difficulties especially in the rainy season is one of the major barriers as the FHSPs from district and province levels need to visit patient residential areas. Case investigation was also difficult if the malaria patient was an MMP or a forest goer because they were moving around or going into the forest especially during the day. FHSPs normally work office hours which prevents them meeting and investigating the MMP malaria patient.

*When I went to their [forest goers’] hut, I didn’t meet them because they work elsewhere in the daytime although they sleep there.*(FHSP, Binh Thuan Province)

When FHSPs do not meet with the patient, they informed nearby people for the next visit and have to return another time, using extra resources for the same case (table 2).

**Table 2:** Summary of facilitators and barriers to implementation and adherence to reactive surveillance and response strategies in Vietnam

Facilitators	Barriers
Overall policy commitment and support for malaria elimination including RASR strategies Existing RASR strategies could capture all secondary malaria cases Using eCDS-MMS for RASR enhanced timely execution of RASR activities Availability of travel and population movement information No language barriers in the two selected provinces	Declined community interest and cooperation in RASR activities Limited availability of electricity and internet connection Shortage of medicines and commodities Limitations in budget and human resources Terrain difficulty Lengthy and complicated case investigation forms Difficulty in meeting and executing case investigation for MMPs and forest goers

eCDS-MMSelectronic Communicable Disease Surveillance System – Malaria Management SystemMMPsmobile and migrant populationsRASRreactive surveillance and response

*If we don't see them today, maybe we meet them another day. We informed their children who are staying in the hut (about next visit). We asked for their help to announce their parents that the CI team will visit and take the blood smear tomorrow. We can visit them in early morning at around 6:00 or 6:30 am. However, the road was bad if raining. So many potholes caused the difficulty for traveling.* (FHSP, Binh Thuan Province)

More than two-thirds of survey participants (58/74, 78.4%) confirmed that the COVID-19 pandemic had an impact on the implementation of RASR activities due to travel restrictions and social distancing (17/58, 29.3%) and a shortage of human resources in malaria programmes as they had to focus on COVID-19 responses (10/58, 17.2%) (table 1). However, FHSPs maintained essential RASR activities with available resources during the COVID-19 pandemic given there was special approval for travel related to RASR activities.

*Decree 16 [A standing order about COVID-19 restrictions] was applied. But the program could still operate RASR because the decree exempted travel related to case investigation.* (FHSP, Binh Thuan Province)

FHSPs from district and commune offices reported issues using the case investigation forms developed as per Decision 741, specifically sections requesting entomological information which cannot be provided at their respective levels. Therefore, they requested staff from province or central levels to join the case and focus investigation teams or suggested unnecessary sections in the forms be excluded.

*We could not use the investigation form from national program because it asks information related to vector densities, species composition and location, and so on. There are a lot of things to investigate. These forms must be completed by staff from province level. I have raised my opinion that province Centre for Diseases Control (CDC) should take care of entomology. District health centres will only do CI, examination, taking blood smear test and communication*.

*It is impossible to call for more supports from NIMPE. So, the Ministry of Health is requested to shorten the form and no longer to ask us to investigate vectors in order reflect the reality of local human resources and insufficient budget*. (FHSPs, Phu Yen Province)

### Acceptability of reactive surveillance and response (RASR) strategies

Staff from commune, district and provincial health departments in the two selected provinces accepted RASR strategies as one of their routine activities dedicated to the malaria elimination programme and followed the strategy despite identified barriers. FHSPs from commune health centres also highlighted the importance of the acceptability of RASR activities among VHWs and local authorities.

*When we [provincial or district medical officials] go to a village, we shall inform the head of a particular village or the secretary of commune communist party ahead of the business trip [for RASR] so that they will cooperate with us in the investigation. The VHWs play an important role in RASR activities in the community. The RASR strategy and RACD need to be supported by this VHW system*. (Stakeholder, Phu Yen Province)

Hence RASR strategies were not only acceptable to healthcare providers, but also among stakeholders from different organisations and facilities such as military medical units and army, commune people’s committees and civil society organisations. They supported field operation of case and focus investigations.

Community members and MMPs were unfamiliar with the name and exact strategy of RASR. They perceived it as one of the activities that promotes their general health. Hence, specific RASR activities (asking travel history of the index case; exploring clinical signs and symptoms of malaria such as fever among household members and close contacts; taking blood smear; rapid diagnostic testing; and some other activities in focus response such as spraying, impregnation of bed net and malaria behavioural change communication) were used as proxy descriptions in discussions to explore acceptability of RASR.

The acceptability and perception of community members including MMPs on RASR activities were not universal. MMPs in Binh Thuan Province viewed RACD positively and were willing to cooperate in RASR activities. Participants expressed willingness to undergo testing to prevent illness, were appreciative that medical staff could perform testing in their homes and even expressed a willingness to undergo additional testing in high transmission seasons.

*It is our desire not to get sick. Sometimes we take part in blood testing and get treated. I don’t wait until I realise I am sick to get the blood test. The medical staff dedicatedly came to my home [for RACD]. In this rainy season, we would like them to take the test once a month because malaria is increasing during rainy season*. (MMP, Binh Thuan Province)

The MMPs followed health centre staff’s instruction when the health staff went to the villages to conduct focus investigations and supported the staff in identifying and locating peer MMPs for RACD. However, some staff from Phu Yen Provincial Health Department experienced resistance from community members to execute some RACD activities and had to provide incentives like medicines and tonics to MMPs and the neighbouring household members who were related to index malaria case to undergo malaria testing.

*When we say we will take blood smears [for RACD], not everyone was willing to let us take blood samples. So, in some localities, we had to pay out of pocket to buy medicines for all patients equally*. (Stakeholder, Phu Yen Province)

## Discussion

This study comprehensively explored the facilitators and barriers to implementation and adherence to RASR strategies in Vietnam. It also examined the acceptance to RASR strategies by health department staff and stakeholders and community members. In Vietnam, RASR strategies have policy commitment and support from the national health system. RASR implementation data is available in the national electronic surveillance system, eCDS-MMS, which enhances timely execution of RASR strategies. However, its usage also demands internet connection which limited the timely notification of some cases detected in forest fringe areas. Case and focus investigations require team effort but are currently limited in terms of budget and human resources particularly for the hard-to-reach foci, and interest in RASR activities and malaria elimination programmes has declined in some communities. The study participants requested more investment for RASR strategies and recommended VHWs to facilitate community engagement to overcome identified logistical barriers and to implement RASR strategies effectively, so they may contribute towards better malaria surveillance and elimination in Vietnam and broadly across the Greater Mekong Subregion.

Malaria elimination and successful implementation of RASR strategy need a collaborative effort, and it can only be achieved with policy commitment and multisectoral coordination.^6^ Overall, Vietnam has a well-established RASR strategy, health system and policy commitment for implementation of RASR activities.^4^ RASR activities are implemented in all 63 provinces, including in 48 provinces declaring malaria-free to ensure imported cases are reported. Health facilities at all levels including village and private health facilities are participating in malaria prevention and elimination including RASR activities. In addition, health agencies and branches of other ministries cooperate in RASR strategies (online supplemental figure 1).^4^ The current close coordination mechanism among different levels in Ministry of Health and with other ministries is a significant facilitator for RASR strategies and therefore malaria elimination in Vietnam, which should be maintained and strengthened in the future.

An identified facilitator of successful RASR implementation in Vietnam is the use of eCDS-MMS. In eCDS-MMS, the malaria surveillance and reporting system are integrated into the infectious diseases reporting system of the Ministry of Health, which plays an important role in improving the quality and reporting time of RASR data and ensuring the implementation of surveillance. Since 2022, eCDS-MMS has included all steps of RASR that captures investigation of cases and malaria foci.^5^ The detection and response to outbreaks in the prescribed time has gradually improved in the following years.^12^ It is monitored by the health system at all levels through tools on the dashboard of eCDS-MMS and the public health emergency operation centre systems for malaria since 2022 which supports the successful implementation of RASR strategies in Vietnam. However, while the transition to an electronic reporting system improved RASR strategies, a barrier to timely case notification was the requirement of internet connection at the site of malaria diagnosis. Even though the infrastructure, including coverage of electricity and internet, has rapidly increased in Vietnam, timely notification of malaria cases detected in forest fringe areas outside of the villages will still be a challenge without universal internet access.

This study did not apply participatory action research including scoring and ranking of key findings by the participants; we, therefore cannot speculate as to the importance of individual facilitators and barriers over others. However, some of the most frequently reported barriers to RASR implementation by all participant groups included insufficient time and funding to deliver RASR activities in a timely manner and in line with current guidelines. Shortage of medicines and supplies for RASR activities was another barrier reported by FHSPs. In Vietnam, there were periodic shortages of antimalarial medicines and supplies, especially in malaria-free areas that implement prevention of reintroduction strategies. Until recent years, the supply of materials and anti-malarial medicines to medical facilities was carried out by National Malaria Program.^16^ Starting from 2022, management of procurement, supply and distribution of medicines and supplies including anti-malarial medicines are decentralised to provinces and are funded with local funds.^2^ Along with the support of an emergency operation centre alert system for shortage of commodities, the issue of shortage of medicines and supplies for RASR activities have been resolved or improved. Effective and timely deployment of budget to all field sites to execute RASR activities was also identified as a barrier to successful RASR implementation. Currently in Vietnam, there are about 500 cases of malaria in about 200 malaria foci in a year that need to be investigated completely and in a timely manner.^3^ To effectively deploy the budget for investigation of all foci, it is necessary to use budget projections and forecasts in many provinces ensuring correct allocation of budget in the future.

The COVID-19 pandemic was identified by the majority of respondents as a barrier to effective RASR implementation. Participants identified social distancing and travel restrictions as the main challenges to RASR implementation as a result of the pandemic. However, despite these reported challenges, malaria programmes, including RASR, continued to operate due to special travel approvals granted by the provincial CDCs, district health centres and commune health stations, ensuring that key surveillance and response activities could still be carried out. A smaller proportion of participants identified diversion of human resources to COVID-19 activities as a challenge to RASR implementation. A significant portion of the healthcare workforce, who typically participated in RASR activities, were reassigned to COVID-19-related tasks. Leadership from provincial CDCs, preventive health systems at the district health centre, and commune health stations were involved in contact tracing and activities directed by the provincial COVID-19 prevention committees. These activities varied depending on the severity of the pandemic in each province, district and commune. These healthcare staff participated in tasks such as COVID-19 testing, contact tracing, vaccination campaigns and treatment of COVID-19 patients. As a result, they had less time to engage in regular RASR activities, leading to delays in the collection, analysis and reporting of malaria surveillance data. Additionally, some health staff, who would typically focus on RASR implementation, were given additional responsibilities such as organising and managing quarantine zones, supporting COVID-19 testing facilities and administering vaccinations. Many of them also participated in community health education about COVID-19 prevention measures, which further reduced their capacity to focus on malaria-related activities. Additionally, it is possible that ongoing preventative measures related to the COVID-19 pandemic including social distancing may have affected participation and data collection despite research activities commencing after major lockdown periods within Vietnam.

In some provinces of high endemicity in Vietnam, access to field sites is still a challenge for primary healthcare staff including activities for case and focus investigation that need to be completed within 7 days. Despite accounts of willingness to participate in RASR and even receive more frequent testing among some participants in qualitative consultations, stakeholders and FHSP reported a reluctance to undergo testing by some community members, particularly if the individual did not have a fever. Further, many malaria cases in Vietnam are concentrated in hard-to-reach areas, and hence the foci are difficult to be accessed and responded even from the nearest medical facility which is typically a commune health centre. This issue is superimposed by limitations in human resources and budget at the commune and district level health centres given commune and district staff are assigned for many primary healthcare programmes in the field. Additionally, although MMPs and forest goers normally provide all travel history when investigated, they are difficult to access for RASR activities. For population groups who move frequently and work in the forest, case and focus investigation are difficult to accomplish. Providing required budget for RASR activities including travel expenses, equipment and supplies for case and focus investigation and responses is important to ensure that the focus clusters are investigated and responded in 7 days. Alternatively, some of the case and focus investigation activities could be deployed to commune health centre staff and VHWs after capacity building with remote supervision by district and provincial staff. To successfully implement RASR activities among hard-to-reach populations such as MMPs and forest goers, the Village Management Committee plays an important role in determining the time of return of MMPs and forest goers to the village so that they can be investigated and tested for malaria. Managing them also requires collaboration of VHWs. With the assistance of VHWs, the transmission points in forests and fields can be identified, and MMPs can be effectively communicated with, screened for fever and malaria, and treated with anti-malarial medicines if necessary.

This study comprehensively explored facilitators, barriers and acceptability of RASR strategies in Vietnam using both quantitative and qualitative methods. The participants ranged from MMPs and forest goers, the malaria at-risk populations, to national level health stakeholders and staff. Differences in participant group roles and responsibilities were reflected in the responses to survey questionnaires. Health stakeholders and staff infrequently gave a specific reason for perceived barriers to implementing RASR activities in a timely manner, but specified funding and staffing issues as barriers to conducting RASR activities under the current guidelines. Conversely, FHSP specified insufficient time, transportation and weather as well as poor co-operation with patients as barriers implementing RASR activities in a timely manner and following RASR guidelines. In both participant groups, respondents more frequently reported perceiving no barrier to implementation of time-bound RASR activities compared with reporting either specific or unspecified barriers. The proportion of health stakeholders/staff that reported barriers to following current RASR implementation guidelines, however, was greater than that of FHSPs and may reflect differences in their respective roles in terms of professional hierarchy and likelihood to report perceived deficits in their role.

Given the range of participant roles in RASR implementation in this study, the findings may be generalised for implementation of RASR strategies across Vietnam to RASR implementors in similar roles. However, the generalisability of some findings, such as no language barrier in implementing RASR, is unclear given that the primary data collection was only undertaken in Binh Thuan and Phu Yen Provinces. The facilitators and barriers and acceptability to RASR strategies should be evaluated according to their local context in both Vietnam but also other areas in the Greater Mekong Subregion implementing RASR strategies. The facilitators and barriers to successful RASR implementation identified by participants in this study are comparable to those reported in assessments of RASR in other Mekong countries. A comprehensive review of RASR implementation in Myanmar reported human resources shortages and limited mobile and internet access in remote areas as major barriers to timely case notification.^17^ Similarly, a recent mixed-methods evaluation in Lao PDR reported telecommunication and transportation difficulties, staff shortages and issues related to contacting MMPs as barriers to effective implementation of RASR activities, as well as additional barriers related to overall RASR awareness among both healthcare workers and end users.^18^ Outside of low malaria transmission settings in the Greater Mekong Subregion, RASR has not been widely taken up as a strategy to achieve malaria declines due to the substantial resources and logistical challenges of implementing aspects such as RACD to regions with high higher case burdens.^19^ However, identifying the facilitators and barriers and investigating the acceptability of any malaria elimination strategy among implementors and end users are important for measuring the strategies impact and optimising future approaches.

In conclusion, RASR strategies are well implemented in Vietnam facilitated by good health system support and policy commitment. To further strengthen its implementation, barriers such as limitations in infrastructure, budget and human resources should be overcome. In order to increase community participation and acceptance in the RASR strategies and overall malaria elimination programme in Vietnam, VHWs could be used with support from the government as needed. Other Greater Mekong Subregion countries may review facilitators and barriers of Vietnam’s RASR strategies and reflect on and explore their own facilitators and barriers in order to contribute to the regional malaria elimination goal.

## supplementary material

10.1136/bmjph-2024-000961 online supplemental file 1

10.1136/bmjph-2024-000961 online supplemental file 2

10.1136/bmjph-2024-000961 online supplemental file 3

10.1136/bmjph-2024-000961 online supplemental file 4

10.1136/bmjph-2024-000961 online supplemental file 5

10.1136/bmjph-2024-000961 online supplemental file 6

## Data Availability

Data are available upon reasonable request. All data relevant to the study are included in the article or uploaded as supplementary information.

## References

[1] World Health Organization (2023). World Malaria Report 2023. 2023 Ed.

[2] World Health Organization (2022). Viet nam - malaria midterm review.

[3] Mekong malaria elimination programme (2023). Malaria Epidemiology Summary In: World Health Organization, Ed. April 2023 Ed.

[4] (2021). The institute of malariology parasitology and entomology and the UCSF malaria elimination initiative. Vietnam malaria transition and sustainability assessment.

[5] Vietnam Ministry of Health (2020). Vietnam national strategic plan on malaria control and elimination, 2021-2025.

[6] World Health Organization (2015). Global technical strategy for malaria 2016-2030.

[7] Lu G, Liu Y, Beiersmann C (2016). Challenges in and lessons learned during the implementation of the 1-3-7 malaria surveillance and response strategy in China: a qualitative study. Infect Dis Poverty.

[8] Cao J, Sturrock HJW, Cotter C (2014). Communicating and monitoring surveillance and response activities for malaria elimination: China’s “1-3-7” strategy. PLoS Med.

[9] Zhou S-S, Zhang S-S, Zhang L (2015). China’s 1-3-7 surveillance and response strategy for malaria elimination: Is case reporting, investigation and foci response happening according to plan?. Infect Dis Poverty.

[10] Vietnam Ministry of Health (2016). Promulgating the “Guidelines for Surveillance and Malaria Prevention”. In: Department of Preventive Medicine, Ed.

[11] Vietnam Ministry of Health (2021). Issuance of "Malaria Surveillance and Prevention Guidelines” In: Department of Preventive Medicine, Ed.

[12] Win Han Oo, Nguyen XT, Ngo TVA (2023). Performance and feasibility of reactive surveillance and response strategies for malaria elimination in Vietnam: a mixed-methods study. Malar J.

[13] von Elm E, Altman DG, Egger M (2007). The Strengthening the Reporting of Observational Studies in Epidemiology (STROBE) statement: guidelines for reporting observational studies. The Lancet.

[14] Green J, Willis K, Hughes E (2007). Generating best evidence from qualitative research: the role of data analysis. Aust N Z J Public Health.

[15] Miles MB, Huberman AM, Saldana J (2020). Qualitative Data Analysis: A Methods Sourcebook 4th Ed.

[16] World Health Organization (2018). National malaria programme review – Viet Nam.

[17] Htike W, Nay Yi Yi Linn, Kyawt Mon Win (2023). Reactive surveillance and response strategies for malaria elimination in Myanmar: a literature review. Malar J.

[18] Htike W, Win Han Oo, Aye Tun N (2024). Comprehensive evaluation of malaria reactive surveillance and response strategies in Lao People’s Democratic Republic: a mixed-methods study. BMJ Open.

[19] Yi B, Zhang L, Yin J (2023). 1-3-7 surveillance and response approach in malaria elimination: China’s practice and global adaptions. Malar J.

