# Supplementary material for: Facilitators, barriers and acceptability of malaria reactive surveillance and response strategies in Vietnam: a mixed-methods study
Source: BMJ Public Health. 2024 Dec 16;2(2):e000961. doi: 10.1136/bmjph-2024-000961 (PMC11816204; doi:10.1136/bmjph-2024-000961)
Supplement: online supplemental file 1 [file bmjph-2-2-s001.pdf]

STROBE Statement—Checklist of items that should be included in reports of *cross-sectional studies*

	Item No	Recommendation	Page No.	Relevant text from manuscript
**Title and abstract**	1	(*a*) Indicate the study's design with a commonly used term in the title or the abstract	1	Facilitators, barriers and acceptability of malaria reactive surveillance and response strategies in Vietnam: **a mixed-methods study**
		(*b*) Provide in the abstract an informative and balanced summary of what was done and what was found	2-3	The study synthesizes opinions and suggestions of RASR practitioners in two provinces of Vietnam on facilitator, barrier and acceptability to RASR implementation. RASR strategies are well-accepted by the malaria program stakeholders and frontline workers, but there were limitations of infrastructure, budget, human resources and terrain difficulties. Community members and mobile and migrant populations could be effectively engaged via community health workers.
**Introduction**				
Background/rationale	2	Explain the scientific background and rationale for the investigation being reported	4-5	The number of malaria cases in Vietnam in 2022 is 455 cases, a sharp decrease compared to the past decades. Vietnam now aims to certify 55 out of 63 provinces as malaria-free by 2025 and is enhancing surveillance as a core intervention and practising 2-7 RASR approach. Since October 2021, either rapid diagnostic test, microscopy or polymerase chain reaction is allowed for confirmation of a malaria case. Case notification and investigation must be completed within two days from diagnosis, followed by the focus investigation and responses within seven days after diagnosis of a malaria case. While the performance and feasibility of the RASR strategy was high (≥79% completeness and timeliness of case and focus investigation), the acceptability of, and facilitators and barriers to its implementation have yet to be explored.
Objectives	3	State specific objectives, including any prespecified hypotheses	5	To describe facilitators, barriers and acceptability to implementing RASR in Vietnam.<br>To explore how the barriers could be overcome to improve the implementation of RASR strategies.
**Methods**				
Study design	4	Present key elements of study design early in the paper	6	The study was designed to combine quantitative and qualitative research, including primary data collection of quantitative surveys, and qualitative

				focus group discussions (FGD) and semi-structured in-depth interviews (IDI).
Setting	5	Describe the setting, locations, and relevant dates, including periods of recruitment, exposure, follow-up, and data collection	6-7	Time: from 11/2021 to 4/2022 for survey in 2 provinces.<br>Location: 2 provinces in Vietnam (Phu Yen, Binh Thuan).<br>The surveys were administered to health stakeholders and staff (n = 36) responsible for managing or supervising RASR and FHSP (n = 38).<br>Data collection: Both quantitative and qualitative data collections were conducted in private locations with confidentiality. Primary language of the participants, Vietnamese, was used in data collection. NIMPE staff interviewed the study participants face-to-face using printed questionnaires and topic guides during surveying, focus group discussing and in-depth interviewing. The survey, in-depth interview and focus group discussion lasted approximately 45, 60 and 90 minutes, respectively.
Participants	6	(*a*) Give the eligibility criteria, and the sources and methods of selection of participants		Selected participants in the study are those who directly carry out RASR activities in 2 provinces of Vietnam, respectively at the provincial, district, commune and village health levels. People who are mobile populations and former patients.
Variables	7	Clearly define all outcomes, exposures, predictors, potential confounders, and effect modifiers. Give diagnostic criteria, if applicable		Participants in the survey: Number of respondents interviewed in the survey, FGD & IDI<br>Age of participants: Completed age in years.<br>Gender: Male and female of participants.<br>Education level: categorical variable with the highest educational level of participants.<br>Role of respondent: job position of the interviewee and the work involved in the malaria program.<br>The reasons for cases that were not investigated: the reasons from the health staffs who did not investigate malaria cases during implementation of the strategy 2-3-7/2-7 in Vietnam period 2017-9/2021.<br>Challenges in conducting case investigation: the challenges from the health staffs and the background where conduct the strategy 2-3-7/2-7 in Vietnam period 2017-9/2021.<br>What was done if the index case was not home when visited for case investigation: what they do for this case in the scenario

				that the index case not at home for case investigation.<br>What is done if someone from the household of the index case is not home, and they cannot be screened for RACD: what they do for this case in the scenario that someone from the index case's household is not at home for RACD.<br>Challenges in conducting screening in the community: challenges they have encountered when they conduct the screening in the community<br>Barriers to timely case notification: Barriers they have encountered in notifying malaria cases in a timely manner<br>Barriers to timely case investigation: Barriers they encountered in investigating malaria cases in a timely manner<br>Barriers to timely foci investigation and response: Barriers they encountered in conducting foci investigation and response activities in a timely manner<br>Barriers to following guidelines for RASR implementation: Barriers they encountered in following their RASR guideline<br>Impact of COVID-19 pandemic on implementing RASR strategies: the impact of COVID-19 pandemic they have experienced when they implement RASR strategies during the pandemic<br>Facilitators to implementing the RASR strategies: The factors facilitating the successful implementation of the RASR strategies as opined by them<br>Acceptability to implementation of RASR strategies: The perception of the malaria program stakeholders and frontline workers towards implementing RASR strategies and the perception of the community members and MMP towards response activities in RASR (e.g., spraying, net distribution, etc.)
Data sources/ measurement	8*	For each variable of interest, give sources of data and details of methods of assessment (measurement). Describe comparability of assessment methods if there is more than one group		Participants in the survey: From the interview of FDG and IDI. Count of number of people for each interview of FDG & IDI.<br>Age of participants: Interview year of birthday of participant of FDG and IDI.<br>Gender: From the interview of FDG and IDI. Categorical variable.<br>Education level: categorical variable and get it from interview in FDG and IDI.<br>Role of respondent: Open-ended questions during surveying, focus group

				discussions and in-depth interviews.<br>The reasons for cases that were not investigated: Open-ended questions during surveying, focus group discussions and in-depth interviews.<br>Challenges in conducting case investigation: Open-ended questions during surveying, focus group discussions and in-depth interviews.<br>What was done if the index case was not home when visited for case investigation: Open-ended questions during surveying, focus group discussions and in-depth interviews.<br>What is done if someone from the household of the index case is not home and they cannot be screened for RACD: Open-ended questions during surveying, focus group discussions and in-depth interviews<br>Challenges in conducting screening in the community: Open-ended questions during surveying, focus group discussions and in-depth interviews<br>Barriers to timely case notification: Open-ended questions during surveying, focus group discussions and in-depth interviews.<br>Barriers to timely case investigation: Open-ended questions during surveying, focus group discussions and in-depth interviews.<br>Barriers to timely foci investigation and response: Open-ended questions during surveying, focus group discussions and in-depth interviews.<br>Barriers to following guidelines for RASR implementation: Open-ended questions during surveying, focus group discussions and in-depth interviews.<br>Impact of COVID-19 pandemic on implementing RASR strategies: Open-ended questions during surveying, focus group discussions and in-depth interviews.<br>Facilitators to implementing the RASR strategies: Open-ended questions during focus group discussion and in-depth interviews<br>Acceptability to implementing the RASR strategies: Open-ended questions during focus group discussions and in-depth interviews
Bias	9	Describe any efforts to address potential sources of bias		Design research group discussion and in-depth interviews, using open-ended questions to cover topics in the RASR

				strategy. Error recalling facts about survey time, survey times, survey days were carefully exploited for open interviews and exchange in group discussions.<br>The investigator was trained and understood the RASR strategy in Vietnam, the interviewees were direct health workers, and leaders of the provincial and district health centres in RASR related activities in the province.<br>All levels and mobile populations have had access to RASR services, household malaria surveys.
Study size	10	Explain how the study size was arrived at		The quantity in the qualitative interview sample for in-depth interviews and group discussions is suitable for the number of people who directly do malaria activities and directly participate in RASR activities and this number also ensures information exploitation in the field. the best group according to qualitative designs of 5-8 people/group and in-depth interviews from 15 people or more
Quantitative variables	11	Explain how quantitative variables were handled in the analyses. If applicable, describe which groupings were chosen and why		Research combines quantitative and qualitative, but in this public, we describe the tables as qualitative. There are a number of quantitative variables such as age in this qualitative study.
Statistical methods	12	(*a*) Describe all statistical methods, including those used to control for confounding	8	The quantitative survey data was typed into the Excel data spreadsheet and imported into Stata version 16.1 for data cleaning, management and analysis. Quantitative descriptive analyses were performed to understand potential barriers and enablers in each step of RASR.<br>Qualitative data organised, managed and analysed in NVivo version 12.<br>The analysis was undertaken using the qualitative descriptive approach.<br>The two authors code the data and interpret the results, then discuss the results to avoid information errors and ensure independent results.<br>A deductive thematic framework that includes coding definitions, themes and subthemes.<br>After coding, the researchers then discussed themes and subthemes to reach a consensus on the final thematic framework and interpretation.<br>The results were reported thematically and as per the outcomes of facilitators and barriers, and acceptability to

				implementing RASR strategies in Vietnam.
		(*b*) Describe any methods used to examine subgroups and interactions		
		(*c*) Explain how missing data were addressed		There is no missing data during field data collection and de-tape, coding, interpretation of results.
		(*d*) If applicable, describe analytical methods taking account of sampling strategy		We used the survey in 2 provinces, the interviewees were people working in the local malaria program and working for the malaria surveillance system. Therefore, 100% of 2 CDCs with 2 district health centers and 4 commune health stations, village health workers in 4 communes were selected to ask questions.
		(*e*) Describe any sensitivity analyses		
**Results**				
Participants	13*	(a) Report numbers of individuals at each stage of study—eg numbers potentially eligible, examined for eligibility, confirmed eligible, included in the study, completing follow-up, and analysed		Individuals including officials at the provincial CDC levels, district health centres, commune health stations, village health stations and mobile populations were recruited in 2 provinces.<br>Number of samples were shown in the table on the study size section (Supplementary table 1 and 2).<br>At each provincial, district and commune level: interviews were conducted, selected subjects are malaria workers, program leaders at all levels and mobile people all agree to participate in the interview.
		(b) Give reasons for non-participation at each stage		
		(c) Consider use of a flow diagram		
Descriptive data	14*	(a) Give characteristics of study participants (eg demographic, clinical, social) and information on exposures and potential confounders		A mixed methods study of quantitative surveys with health stakeholders and staff (n = 36) and frontline health services providers (n = 38), and qualitative focus group discussions with frontline health services providers and mobile migrant populations (n = 70) and semi-structured in-depth interview with health stakeholders and staff (n = 28) was conducted in Binh Thuan and Phu Yen Provinces in Vietnam.<br>The average age is 41 years old, 34 women accounted for 46%. There are 61 people with college, university and post-graduate professional degrees. There are a number of people with qualifications from level 1, 2, 3 who are mobile people

				participating in the study
		(b) Indicate number of participants with missing data for each variable of interest		No missing data is present in the survey
Outcome data	15*	Report numbers of outcome events or summary measures		Participants in the survey: Description of number of participants in the research object.<br>Age of participants: Description of year of birthday of participant of FDG and IDI.<br>Gender: Description of gender of participant and proportion.<br>Education level: Description of education level of participant and proportion.<br>Role of respondent: Description of Role of respondent and position in the malaria program.<br>The reasons for cases that were not investigated: Describe some of the reasons for the failure to investigate cases.<br>Challenges in conducting case investigation: List some of the challenges at the study sites for carrying out case investigations.<br>What was done if the index case was not home when visited for case investigation: Describe the events, or next steps to take, after the reported case is absent from the village or home in the past.<br>What is done if someone from the household of the index case is not home, and they cannot be screened for RACD: Describe a plan to conduct a cluster investigation when the indicated case is not at home.<br>Challenges in conducting screening in the community: Describe some of the real challenges of conducting cluster investigations in the community.<br>Barriers to timely case notification: Describe the barriers in notifying malaria cases in a timely manner.<br>Barriers to timely case investigation: Describe the barriers in investigating malaria cases in a timely manner.<br>Barriers to timely foci investigation and response: Describe the barriers in conducting foci investigation and response activities in a timely manner.<br>Barriers to following guidelines for RASR implementation: Describe the barriers to following the guidelines for implementing the RASR activities.<br>Impact of COVID-19 pandemic on implementing RASR strategies: Describe

				the impact of COVID-19 pandemic on RASR strategy.<br>Facilitators to implementing the RASR strategies: Describe the facilitators enabling the successful implementation of RASR strategies<br>Acceptability to implementing the RASR strategies: Describe the perception of malaria program stakeholders, frontline workers and community members including mobile and migrant populations regarding the acceptability to the implementation of RASR activities.
Main results	16	(*a*) Give unadjusted estimates and, if applicable, confounder-adjusted estimates and their precision (eg, 95% confidence interval). Make clear which confounders were adjusted for and why they were included	9-18	The factors related to the challenge were collected and grouped from the opinions of the interviewees. Confounding factors are removed in compliance with national monitoring guidelines.<br>Respondents reported that commitment by all stakeholders and having the national policy support for malaria elimination and RASR strategies as a facilitator of the successful implementation of RASR in Vietnam.<br>Although 27/75, 39.1% of respondents mentioned there was no barrier to follow current guidelines for implementation of RASR activities, 14/75, 20.3% of respondents claimed that lack of awareness about malaria elimination and poor cooperation from patient, terrain difficulty and insufficient resources including funding (12/75, 17.4%) were common barriers.<br>Staff from commune, district and provincial health departments in the two selected provinces agreed, accepted and followed the RASR strategy. Despite challenges, they perceived that it is one of their routine activities dedicated to malaria elimination program.
		(*b*) Report category boundaries when continuous variables were categorized		
		(*c*) If relevant, consider translating estimates of relative risk into absolute risk for a meaningful time period		
Other analyses	17	Report other analyses done—eg analyses of subgroups and interactions, and		

		sensitivity analyses		
**Discussion**				
Key results	18	Summarise key results with reference to study objectives	18-19	This study comprehensively explored the facilitators and barriers to implementation and adherence to RASR strategies in Vietnam. It also examined the acceptance to RASR strategies by health department staff and stakeholders, and community members. In Vietnam, RASR strategies have policy commitment and support from the national health system. Its implementation data is mainstreamed in the national electronic surveillance system, eCDS-MMS, which enhances timely execution of RASR strategies. However, its usage also demands internet connection which limited the timely notification of some cases detected in forest fringe areas. Case and focus investigations require team effort but is currently limited in terms of budget and human resources particularly for the hard-to-reach foci, and interest in RASR activities and malaria elimination programs has declined in some communities (Table 2). The study participants requested more investment for RASR strategies and recommended VHWs facilitate community engagement to overcome identified logistical barriers and to implement RASR strategies effectively so they may contribute towards better malaria surveillance and elimination in Vietnam and broadly across the GMS.
Limitations	19	Discuss limitations of the study, taking into account sources of potential bias or imprecision. Discuss both direction and magnitude of any potential bias		The limitation of the study is the difference in malaria surveillance in Vietnam with the criteria for communes, districts and provinces in the malaria elimination phase compared with the WHO standards for the malaria elimination phase: In Vietnam, though a province has at least one commune in the malaria control phase, the whole province will be in the malaria elimination phase. Anyway, the methods and conventions to ensure that activities are consistent with those of WHO guidelines. Vietnam's provinces currently have 42 provinces in the malaria control phase and 21 in the malaria control and elimination phase, of which there are about 10 provinces in the malaria control phase, but the provinces are nationwide still implementing the RASR strategy.

				The eCDS software is gradually improving to manage RASR, the study was carried out during the malaria transition period, which was attached to the eCDS software.<br><br>The implementation phase of the study was also the period of updating the monitoring guidelines, some of the timelines in the RASR timelines were also changed and updated.
Interpretation	20	Give a cautious overall interpretation of results considering objectives, limitations, multiplicity of analyses, results from similar studies, and other relevant evidence	19-21	Malaria elimination and successful implementation of RASR strategy needs a collaborative effort and it can only be achieved with policy commitment and multisectoral coordination.6 Overall, Vietnam has a well-established RASR strategy, health system and policy commitment for implementation of RASR activities.4 Health facilities at all levels including village and private health facilities are participating in malaria prevention and elimination including RASR activities. In addition, health agencies and branches of other ministries cooperate in RASR strategies (Additional file 3, Supplementary figure 1).4 The current close coordination mechanisms among different levels in Ministry of Health and with other ministries is a significant facilitator for RASR strategies, and therefore malaria elimination, in Vietnam which should be maintained and strengthened in the future.<br><br>An identified facilitator of successful RASR implementation in Vietnam is the use of eCDS-MMS. In eCDS-MMS, the malaria surveillance and reporting system is integrated in the infectious diseases reporting system of the Ministry of Health, which plays an important role in improving the quality and reporting time of RASR data and ensuring the implementation of surveillance.<br><br>However, while the transition to an electronic reporting system improved RASR strategies, a barrier in timely case notification was the requirement of internet connection at the site of malaria diagnosis. Even though the infrastructure, including coverage of electricity and internet has rapidly increased in Vietnam, timely notification of malaria cases detected in forest fringe

				areas outside of the villages will still be a challenge without universal internet access.<br><br>Shortage of medicines and supplies for RASR activities was another barrier reported by FHSPs. In Vietnam, there were periodic shortages of antimalarial medicines and supplies, especially in malaria free areas that implement Prevention of Reintroduction strategy. Until recent years, the supply of materials and anti-malarial medicines to medical facilities was carried out by National Malaria Program. Starting from 2022, management of procurement, supply and distribution of medicines and supplies including antimalaria medicines are decentralised to provinces and are funded with local funds. Along with the support of emergency operation centre alert system for shortage of commodities, the issue of shortage of medicines and supplies for RASR activities have been resolved or improved.<br><br>In some provinces of high endemicity in Vietnam, access to field sites is still a challenge for primary healthcare staff including activities for case and focus investigation that need to be completed within seven days. Many malaria cases in Vietnam are concentrated in hard-to-reach areas and hence the foci are difficult to be accessed and responded even from the nearest medical facility which is typically a commune health centre. This issue is superimposed by limitations in human resources and budget at the commune and district level health centres given commune and district staff are assigned for many primary healthcare programs in the field.<br><br>Alternatively, some of the case and focus investigation activities could be deployed to commune health centre staff and VHWs after capacity building with remote supervision by district and provincial staff.<br><br>With the assistance of VHWs, the transmission points in forests and fields

				can be identified, and MMPs can be effectively communicated with, screened for fever and malaria, and treated with antimalarial medicines if necessary.<br><br>Other GMS countries may review facilitators and barriers of Vietnam's RASR strategies and explore their own facilitators and barriers in order to contribute to the regional malaria elimination goal.
Generalisability	21	Discuss the generalisability (external validity) of the study results		The findings from this study could be generalised for implementation of RASR strategies across Vietnam. However, some of the findings such as no language barrier in implementing RASR may be warranted in application in other provinces given the primary data collection only happened in Binh Thuan and Phu Yen Provinces. Further, facilitator and barriers, and acceptability to RASR strategies currently being implemented in other GMS countries should be evaluated according to their local context.
**Other information**				
Funding	22	Give the source of funding and the role of the funders for the present study and, if applicable, for the original study on which the present article is based		HPA involvement in research design and field support. Sponsoring data collection activities and medical ethics committees in Vietnam.

*Give information separately for exposed and unexposed groups.

Note: An Explanation and Elaboration article discusses each checklist item and gives methodological background and published examples of transparent reporting. The STROBE checklist is best used in conjunction with this article (freely available on the Web sites of PLoS Medicine at http://www.plosmedicine.org/, Annals of Internal Medicine at http://www.annals.org/, and Epidemiology at http://www.epidem.com/). Information on the STROBE Initiative is available at www.strobe-statement.org.
