# Supplementary material for: Facilitators, barriers and acceptability of malaria reactive surveillance and response strategies in Vietnam: a mixed-methods study
Source: BMJ Public Health. 2024 Dec 16;2(2):e000961. doi: 10.1136/bmjph-2024-000961 (PMC11816204; doi:10.1136/bmjph-2024-000961)
Supplement: online supplemental file 2 [file bmjph-2-2-s002.pdf]

Additional file 2: Questionnaires for surveying the malaria program stakeholders

Questionnaire 1: Malaria program stakeholders responsible for managing or supervising field reactive surveillance and response activities

**General background Information**		
0.1	Date (DD/MM/YYYY):	
0.2	Country:	
0.3	Name of organization	
**Background information of Malaria Program Stakeholder (manager/supervisor of field activities)**		
0.4	Completed age (in years):	
0.5	Gender	1. Male<br>2. Female<br>3. Other/prefer not to say
0.6	Highest education level	1. No formal education<br>2. Primary school level<br>3. Secondary school level<br>4. High school level<br>5. Degree holder<br>6. Others (specify)
0.7	What is your current role	
0.8	How long have you worked in your current role	_______years _______ months

**Section 1: General information on reactive surveillance and response approaches and malaria elimination programme**		
	Does your malaria programme follow (or aim to follow) a time-bound strategy for case reporting, case investigation and response activities?	1. No<br>2. Yes, the 1-3-7 approach<br>3. Yes, another approach (please specify):
	Where does this time-bound strategy for case reporting, case investigation and response activities apply?	1. Areas in elimination phase<br>2. All areas<br>3. Other (please specify)

**Section 2: Case Notification**		
	How are positive malaria cases initially reported?	1. Paper based reporting<br>2. Electronic reporting system<br>3. Telephone call<br>4. Messaging program (e.g. WhatsApp)
	In your opinion, how frequently are cases reported within 24 hours of diagnosis?	1. Never<br>2. Occasionally (less than 20%)

		3. Sometimes (20-50%)<br>4. More often than not (50-75%)<br>5. Usually (more than 75%)<br>6. Nearly always (more than 90%)

**Section 2: Case Investigation**		
	What event triggers a case investigation?	1. Case reported to national level<br>2. Case reported to peripheral level<br>7. Other, please specify:
	What is the policy for conducting a case investigation	1. All indigenous <u>and</u> imported cases<br>2. Indigenous cases <u>only</u><br>3. Imported cases <u>only</u><br>4. Other – please specify:
	To your knowledge, how frequently is case investigation completed for positive malaria cases	1. For all cases (100%)<br>2. For <20% of cases<br>3. Between 20% and 50% of cases<br>4. Between 50% and 75% of cases<br>5. More than 75% of cases but less than 100%
	If all cases are not investigated, what are the main reasons these cases are not investigated?	1. It is an imported case<br>2. It is outside of the district of the person investigating<br>3. The person could not be found<br>4. Not enough staff/resources<br>5. Daily cross-border case<br>6. Not applicable – every case is investigated<br>7. Other – please specify:
	What occurs for cases that are not investigated?	
	Is there a specific person in the malaria programme who is responsible for overseeing case investigations?	1. Yes, please specify title/role<br>2. No
	Who is responsible for performing case investigations in your malaria programme?	1. Village Health Volunteers or equivalent<br>2. Add other options
	Is this person trained in case investigation techniques?	1. Yes<br>2. No
	If so, how frequent is the training relating to case investigations?	1. Monthly<br>2. Quarterly<br>3. Yearly<br>4. Every second year

	Are personnel conducting investigations periodically supervised by managers?	1. Yes<br>2. No
	How regularly are personnel conducting investigations supervised by managers or other superiors?	1. Monthly<br>2. Quarterly<br>3. Yearly<br>4. Other – please specify
	How soon after a positive case is recorded is a case investigation initiated?	1. Within 24 hours<br>2. Within 48 hours<br>3. Within 72 hours<br>4. Within one week<br>5. Within one month
	Is there an SOP for case investigation?	1. Yes<br>2. No
	Is there a specific case investigation form to use when conducting investigations?	1. Yes<br>2. No
	Does case investigation involve visiting the index case?	1. Yes, always<br>2. No, never<br>3. Yes, sometimes
	When doing a case investigation, which of the following best describes how an appointment is made with the index case (choose one)	1. Telephone the index case<br>2. No prior communication – go to the index case residence to see if they are home<br>3. Other – please specify
	What is done if the index case is not home when they visit? (select all that apply)	1. Visit a second time: later that day or on a subsequent day<br>2. Telephone to schedule an appointment<br>3. We mark the case as imported<br>4. We mark the case as "not found"<br>5. We do not re-visit the index case<br>6. Inform volunteers to make appointment with the case<br>7. Other – Please specify:
	What time of day do you normally investigate cases? (choose all that apply)	1. Right after case detected at health facility<br>2. Before 8am<br>3. Between 8am-12noon<br>4. Between 12noon-5pm<br>5. After 5pm<br>6. Weekends<br>7. Whenever the team is available

	If all cases are not investigated, what are the main reasons these cases are not investigated?<br>(select all that apply)	1. It is an imported case<br>2. It is outside of my district<br>3. The person could not be found<br>4. Not enough staff/resources<br>5. Daily cross-border case<br>6. Not applicable – we investigate every case<br>7. Other – Please specify:
	Is supervised treatment conducted for positive cases?	1. Yes<br>2. No
	Is follow-up on adherence to treatment conducted for each case?	1. Yes<br>2. No
	Do case investigations involve checking on malaria prevention measures used by the index case?	1. Yes<br>2. No
	Do case investigations involve educating the index case on malaria risk factors and prevention?	1. Yes<br>2. No
	What information is used to determine if an index case is imported or local? Case has travelled to another endemic area (choose all that apply)	1. Village<br>2. District<br>3. Province/State<br>4. Region<br>5. Country<br>6. Any of the above<br>7. Other – please specify:
	Do case investigations involve mapping the location of the index case and if so how is this performed? E.g. geographical information system (GIS) to make the maps.	1. No<br>2. Yes, explain:
X	Does your programme collect information from index cases on their travel history?	1. Yes<br>2. No
	If yes to X, does your programme collect information on travel within the district of residence?	1. Yes<br>2. No
	If yes to X, does your programme collect information on travel outside the district of residence?	1. Yes<br>2. No
	If yes to X, does your programme collect information on travel outside of the country?	1. Yes<br>2. No

	How does your programme define imported cases?	1. Cases originating in another country<br>2. Cases occurring within the country but from a different province, district, or other administrative unit<br>3. Other, specify____
	Does your programme/country collect data and report on intra-country importation of cases (e.g. from different districts)	1. Yes<br>2. No
	What are some of the challenges in conducting case investigation?	

**Section 3: Foci Investigations and Reactive Case Detection**		
	Does your programme routinely conduct foci investigations?	1. Yes<br>2. No
Y	Does your programme routinely conduct reactive case detection?	1. Yes<br>2. No
3.2	If yes to Y, what is the trigger for reactive case detection in your programme/country?	3. Every indigenous case is a trigger (e.g. one case identified through passive case detection considered to be local)<br>4. All imported cases irrespective of duration of stay<br>5. Imported cases if they have stayed more than a certain number of days in country, specify no. days____
	How soon after a positive case is <u>recorded</u> is a foci investigation initiated (where necessary)?	1. Within 24 hours<br>2. Within 48 hours<br>3. Within 72 hours<br>4. Within seven days<br>5. Within 14 days<br>6. Within 28 days
	How soon after a positive case is <u>investigated</u> is a foci investigation initiated (where necessary)?	1. Within 24 hours<br>2. Within 48 hours<br>3. Within 72 hours<br>4. Within seven days<br>5. Within 14 days<br>6. Within 28 days
	What is the threshold number of infections, identified through passive case detection that triggers reactive case detection in your programme?	

	Do you screen household members of the index case?	1. Always<br>2. Never<br>3. Sometimes – please explain:
	When screening household members do you screen:	1. Febrile cases only<br>2. All household members (asymptomatic and febrile cases)<br>3. We do not screen household members of a positive case
	What is done if someone from the household of the index case is not home and they cannot be screened? (Check all that apply)	1. Visit the household later that day or on a subsequent day<br>2. Schedule an appointment with the household members to return<br>3. We do not return<br>4. Other – Please specify:
	How often do you screen neighbours of the index case in the community?	1. Always<br>2. Sometimes<br>3. Never
	When screening neighbours of the index case, are febrile individuals tested or all individuals	1. Febrile neighbours only<br>2. All neighbours<br>3. Not applicable
	What triggers screening in the community?	1. Local cases only<br>2. Local and imported cases<br>3. Imported cases only<br>4. When local cases reach a minimum threshold (please specify below):
	Do you screen a minimum number of households around a positive index case	1. No<br>2. Yes (specify minimum number of households):
	Do you screen a minimum number of people around a positive index case	1. No<br>2. Yes (specify minimum number of individuals):
	Do you screen within a minimum geographic radius around a positive index case	1. No<br>2. Yes (specify number of meters radius screened):
	What time of day do you normally conduct screening in the community? (Circle all that apply)	1. Right after case is detected at the health facility or in the community<br>2. Before 8am<br>3. Between 8am-12noon<br>4. Between 12noon-5pm<br>5. After 5pm<br>6. Weekdays<br>7. Weekends<br>8. Whenever the team is available

	If someone is missing at the time of screening in the community, do you return to screen them?	1. Yes<br>2. No<br>3. If you do not return, what do you do to reach that individual?
	When conducting RACD, which diagnostic method is used (mark all that apply)<br>are used and some are used in combination with others for diagnosis confirmation and speciation (results not mutually exclusive). All (13) respondents reported using microscopy, seven use rapid diagnostic tests (RDT), five use polymerase chain reaction (PCR), two use clinical diagnosis, and one uses serology (Table 5).	1. Microscopy<br>2. Rapid diagnostic test (RDT)<br>3. Polymerase chain reaction (PCR)<br>4. Clinical diagnosis<br>5. Serology
	What is the trigger for reactive case detection in your malaria program	1. Single confirmed case<br>2. >1 confirmed case within a specified radius<br>3. Other threshold of confirmed cases (describe)____________________
	What are some of the challenges in conducting screening in the community?	

**Section 4: Response activities**		
	What kinds of response activities may be triggered when a malaria case or focus is identified?	1. Raising awareness about causes of malaria transmission<br>2. Raising awareness about malaria prevention<br>3. Providing additional vector control if needed<br>4. Entomological surveillance<br>5. If entomological surveillance not possible, performing spot checks for mosquito breeding grounds
	How soon after a malaria case is initially reported are response activities usually commenced?	1. Within 24 hours<br>2. Within 48 hours<br>3. Within 72 hours<br>4. Within seven days<br>5. Within 14 days<br>6. Within 28 days

	How soon after a malaria case is investigated are response activities usually commenced?	1. Within 24 hours<br>2. Within 48 hours<br>3. Within 72 hours<br>4. Within seven days<br>5. Within 14 days<br>6. Within 28 days
	How soon after a malaria foci is initially investigated are response activities usually commenced?	1. Within 24 hours<br>2. Within 48 hours<br>3. Within 72 hours<br>4. Within seven days<br>5. Within 14 days<br>6. Within 28 days
	Within your malaria program are there any reactive surveillance and response activities specifically targeted to mobile and migrant populations, including forest-goers?	Need to refine/expand this question
	In your experience, does information gained from case and foci investigations and classification influence the kinds of response activities that are carried out. If so, how?	
	Do you think current reactive surveillance and response activities are sufficient for targeting P. vivax malaria?	Need to refine/expand this question

**Section 5: Facilitators and Barriers to implementation and adherence to current reactive surveillance and response strategies**		
	What do you think are the current barriers (if any) to conducting case notification within 1 day?	Add options or keep as open ended??
	What do you think are the current barriers (if any) to conducting case investigations within 3 days?	
	What do you think are the current barriers (if any) to conducting foci investigation and response within 7 days?	
	What are the current barriers to following current guidelines for implementation of	

	reactive surveillance and response activities?	
	In your experience, has the COVID-19 pandemic had an impact on successful implementation of reactive surveillance and response strategies?	

Questionnaire 2 for surveying frontline malaria service providers

General background Information		
0.1	Date (DD/MM/YYYY):	
0.2	Country:	
0.3	Name of organization:	
**Demographic information on frontline malaria service provider**		
0.4	Completed age (in years):	
0.5	Gender	1. Male<br>2. Female<br>3. Other/Prefer not to say
0.6	Highest education level	7. No formal education<br>8. Primary school level<br>9. Secondary school level<br>10. High school level<br>11. Degree holder<br>12. Others (specify)
0.7	What is your current role?	
0.8	How long have you worked in your current role	_______years _______ months

Section 1: Village/worksite characteristics		
1.1	Approximately how many households are in your village/worksite(s) or catchment area (for health facility)?	____ households
1.2	What is the approximate total population size of your village/worksite(s) or catchment area (for health facility)?	____ persons
1.3	Is there mobile phone signal in your village/worksite (s)? (Any SIM card)	1. Yes<br>2. No
1.4	If yes, is the mobile phone signal good at your village/worksite(s)?	1. Yes<br>2. No
1.5	Is there Internet access at your village/worksite(s)?	1. Yes<br>2. No
1.6	If yes, is the internet access good at your village/worksite(s)?	1. Yes<br>2. No

Section 2: Malaria testing and case notification		
3.1	On average, how many malaria diagnostic tests do you perform in one month?	____ tests

3.2	On average, how many days per month do you perform malaria rapid diagnostic tests?	___ days
3.3	In general, how are you notified about possible cases of malaria in your village/worksite(s)? **(Select all that apply)**	1. Phone call from patient<br>2. Phone call/referral from patient's family member or friend<br>3. Patient's visit to volunteer<br>4. House visit by volunteer<br>5. Mass testing<br>6. Other (Specify):
	How do you <u>first</u> report positive malaria cases to your supervisor/organization?	1. Paper based reporting<br>2. Electronic reporting system (e.g. mobile or tablet)<br>3. Telephone call<br>4. Messaging program (e.g. WhatsApp)
	How frequently are you able to report malaria cases within 24 hours of diagnosis?	1. Never<br>2. Occasionally (less than 20%)<br>3. Sometimes (20-50%)<br>4. More often than not (50-75%)<br>5. Usually (more than 75%)<br>6. Nearly always (more than 90%)

**Section 3: General information on reactive surveillance and response approaches and malaria elimination programme**		
	Within the malaria programme you work for, are you aware of a time-bound strategy for case reporting, case investigation and response activities?	1. No<br>2. Yes, the 1-3-7 approach<br>3. Yes, another approach (please specify)
	Where does this time-bound strategy for case reporting, case investigation and response activities apply?	1. Areas in elimination phase<br>2. All areas<br>3. Other (please specify)

**Section 4: Case Investigation**		
	What is the policy for conducting a case investigation in your malaria programme and area?	1. All indigenous <u>and</u> imported cases<br>2. Indigenous cases <u>only</u><br>3. Imported cases <u>only</u><br>4. Other – please specify:<br>5. Don't know
	What event triggers a case investigation?	1. Case reported to national level<br>2. Case reported to peripheral level<br>3. Other, please specify:<br>4. Don't know

	Are you personally involved in malaria case investigations?	1. Yes, always<br>2. Yes, sometimes<br>3. No, never
	If you are involved in malaria case investigations are you supervised in these activities?	1. Yes, by ____<br>2. No
	If you are not involved in case investigations, would you be willing to be involved in them in future?	1. Yes<br>2. No
	With respect to positive malaria cases identified by you or referred to you, how frequently is case investigation completed?	1. For all cases (100%)<br>2. For <20% of cases<br>3. Between 20% and 50% of cases<br>4. Between 50% and 75% of cases<br>5. More than 75% of cases but less than 100%
	If all cases are not investigated, what are the main reasons these cases are not investigated?	1. It is an imported case<br>2. It is outside of the district of the person investigating<br>3. The person could not be found<br>4. Not enough staff/resources<br>5. Daily cross-border case<br>6. Not applicable – every case is investigated<br>7. Other – please specify:
	What occurs for cases that are not investigated?	
	How regularly are personnel conducting investigations supervised by managers or other superiors?	1. Monthly<br>2. Quarterly<br>3. Yearly<br>4. Other – please specify
	How soon after a positive case is recorded is a case investigation initiated?	1. Within 24 hours<br>2. Within 48 hours<br>3. Within 72 hours<br>4. Within one week<br>5. Within one month
	Is there an SOP for case investigation?	1. Yes<br>2. No
	Do you follow the SOP for case investigations when required to conduct case investigations?	1. Yes<br>2. No
	Have you personally been trained to conduct case investigations?	1. Yes<br>2. No

	If so, how frequent is the training for case investigations?	1. Monthly<br>2. Quarterly<br>3. Yearly<br>3. Every second year
	Is there a specific case investigation form to use when conducting investigations?	1. Yes<br>2. No
	Does case investigation involve visiting the index case?	1. Yes, always<br>2. No, never<br>3. Yes, sometimes
	When doing a case investigation, which of the following best describes how you make an appointment with that index case? (choose one)	1. Telephone the index case<br>2. No prior communication – go to the index case residence to see if they are home<br>3. Other – please specify
	What do you do if the index case is not home when you visit? (select all that apply)	1. Visit a second time: later that day or on a subsequent day<br>2. Telephone to schedule an appointment<br>3. We mark the case as imported<br>4. We mark the case as "not found"<br>5. We do not re-visit the index case<br>6. Inform volunteers to make appointment with the case<br>7. Other – Please specify:
	What time of day do you normally investigate cases? (choose all that apply)	1. Right after case detected at health facility<br>2. Before 8am<br>3. Between 8am-12noon<br>4. Between 12noon-5pm<br>5. After 5pm<br>6. Weekends<br>7. Whenever the team is available
	If all cases are <u>not</u> investigated, what are the main reasons these cases are not investigated?<br>(select all that apply)	1. It is an imported case<br>2. It is outside of my district<br>3. The person could not be found<br>4. Not enough staff/resources<br>5. Daily cross-border case<br>6. Not applicable – we investigate every case<br>7. Other – Please specify:
	Do you conduct supervised treatment for positive cases?	1. Always<br>2. Sometimes<br>3. Never

	Do you conduct follow-up on adherence to treatment for each case?	1. Always<br>2. Sometimes<br>3. Never
	During case investigations do you check on malaria prevention measures used by the index case?	
	Do case investigations involve educating the index case on malaria risk factors and prevention?	
	What information is used to determine if an index case is imported or local? Case has travelled to another endemic area (choose all that apply)	1. Village<br>2. District<br>3. Province/State<br>4. Region<br>5. Country<br>6. Any of the above<br>7. Other – please specify:
	Do case investigations involve mapping the location of the index case and if so how is this performed? E.g. geographical information system (GIS) to make the maps.	1. Yes<br>2. No
X	Does your programme collect information from index case on their travel history?<br>Nine programmes participating in the survey collect information on whether the index case has had any recent contact with travellers or immigrants. Only three countries gather patient history of glucose-6-phosphate dehydrogenase (G6PD) deficiency, an inherited blood disorder prevalent in many malaria-endemic areas.	1. Yes<br>2. No
	If yes to X, does your programme collect information on travel within the district of residence? , outside the district of resi	1. Yes<br>2. No
	If yes to X, does your programme collect information on travel outside the district of residence?	1. Yes<br>2. No
	If yes to X, does your programme collect information on travel outside of the country?	1. Yes<br>2. No

	How does your programme define imported cases?	1. Cases originating in another country<br>2. Cases occurring within the country but from a different province, district, or other administrative unit<br>3. Other, specify____
	Does your programme/country collect data and report on intra-country importation of cases (e.g. from different districts)	1. Yes<br>2. No
	What are some of the challenges in conducting case investigation?	
	What role do you think malaria service providers like yourself should play in case investigation?	

**Section 3: Foci Investigations and Reactive Case Detection**		
3.2	What is the trigger for reactive case detection in your programme/country?	1. Every indigenous case is a trigger (e.g. one case identified through passive case detection considered to be local)<br>2. All imported cases irrespective of duration of stay<br>3. Imported cases if they have stayed more than a certain number of days in country, specify no. days____
3.1	What is the threshold number of infections, identified through passive case detection that triggers reactive case detection in your programme?	
	Do you screen household members of the index case?	1. Always<br>2. Never<br>3. Sometimes – please explain:
3.3	When screening household members do you screen:	1. Febrile cases only<br>2. All household members (asymptomatic and febrile cases)<br>3. We do not screen household members of a positive case
	What is done if someone from the household of the index case is not home and they cannot be screened? (Check all that apply)	1. Visit the household later that day or on a subsequent day<br>2. Schedule an appointment with the household members to return<br>3. We do not return

		4. Other – Please specify:
	How often do you screen neighbours of the index case in the community?	1. Always<br>2. Sometimes<br>3. Never
	When screening neighbours of the index case, are febrile individuals tested or all individuals	1. Febrile neighbours only<br>2. All neighbours<br>3. Not applicable
	What triggers screening in the community?	1. Local cases <u>only</u><br>2. Local <u>and</u> imported cases<br>3. Imported cases <u>only</u><br>4. When local cases reach a minimum threshold (please specify below):
	Do you screen a minimum number of households around a positive index case	1. No<br>2. Yes (specify minimum number of households):
	Do you screen a minimum number of people around a positive index case	1. No<br>2. Yes (specify minimum number of indviduals):
	Do you screen within a minimum geographic radius around a positive index case	1. No<br>2. Yes (specify number of meters radius screened):
	What time of day do you normally conduct screening in the community? (Circle all that apply)	1. Right after case is detected at the health facility or in the community<br>2. Before 8am<br>3. Between 8am-12noon<br>4. Between 12noon-5pm<br>5. After 5pm<br>6. Weekdays<br>7. Weekends<br>8. Whenever the team is available
	If someone is missing at the time of screening in the community, do you return to screen them?	1. Yes<br>2. No<br>3. If you do not return, what do you do to reach that individual?
	When conducting RACD, which diagnostic method is used (mark all that apply)<br>are used and some are used in combination with others for diagnosis confirmation and speciation (results not mutually exclusive). All (13) respondents reported using microscopy, seven use rapid diagnostic tests (RDT), five use	1. Microscopy<br>2. Rapid diagnostic test (RDT)<br>3. Polymerase chain reaction (PCR)<br>4. Clinical diagnosis<br>5. Serology

	polymerase chain reaction (PCR), two use clinical diagnosis, and one uses serology (Table 5).	
	To your knowledge, what is the trigger for reactive case detection in your malaria program	1. Single confirmed case<br>2. >1 confirmed case within a specified radius<br>3. Other threshold of confirmed cases (describe)____________________<br>4. Don't know
	What are some of the challenges in conducting screening in the community?	

**Section 4: Response activities**		
4.1	In your role, what response activities are you involved in following identification of a malaria focus? (select all that apply)	1. Raising awareness about causes of malaria transmission<br>2. Raising awareness about malaria prevention<br>3. Providing additional vector control if needed<br>4. Entomological surveillance<br>5. If entomological surveillance not possible, performing spot checks for mosquito breeding grounds
	In your experience, does information gained from case and foci investigations and classification influence the kinds of response activities that are carried out. If so, how?	6.
	How soon after a malaria case is initially <u>reported</u> are response activities usually commenced?	1. Within 24 hours<br>2. Within 48 hours<br>3. Within 72 hours<br>4. Within seven days<br>5. Within 14 days<br>6. Within 28 days
	How soon after a malaria case is <u>investigated</u> are response activities usually commenced?	1. Within 24 hours<br>2. Within 48 hours<br>3. Within 72 hours<br>4. Within seven days<br>5. Within 14 days<br>6. Within 28 days
	How soon after a <u>malaria foci is initially investigated</u> are response activities usually commenced?	1. Within 24 hours<br>2. Within 48 hours<br>3. Within 72 hours

		4. Within seven days<br>5. Within 14 days<br>6. Within 28 days

**Section 5: Facilitators and Barriers to implementation and adherence to current reactive surveillance and response strategies**		
	What do you think are the current barriers (if any) to conducting case notification within 1 day?	Add options or keep as open ended??
	What do you think are the current barriers (if any) to conducting case investigations within 3 days?	
	What do you think are the current barriers (if any) to conducting foci investigation and response within 7 days?	
	What are the current barriers to following current guidelines for implementation of reactive surveillance and response activities?	
	Do you have any suggestions for how current reactive surveillance and response activities can be improved?	
	In your experience, has the COVID-19 pandemic had an impact on successful implementation of reactive surveillance and response strategies	
