# Supplementary material for: Facilitators, barriers and acceptability of malaria reactive surveillance and response strategies in Vietnam: a mixed-methods study
Source: BMJ Public Health. 2024 Dec 16;2(2):e000961. doi: 10.1136/bmjph-2024-000961 (PMC11816204; doi:10.1136/bmjph-2024-000961)
Supplement: online supplemental file 3 [file bmjph-2-2-s003.pdf]

Supplementary tables

Supplementary Table 1: Background information of study participants in the survey

Characteristic	Health stakeholders/ staff	Frontline health services providers (FHSPs)	Total
Number of respondents interviewed	36	38	74
Completed age in years, mean (Standard Deviation (SD))	42.3 (10.4)	41.3 (10.0)	41.8 (10.1)
Gender, n (%)			
Male	26 (72.2)	14 (36.8)	40 (54.1)
Female	10 (27.8)	24 (63.2)	34 (45.9)
Highest education level, n (%)			
No formal education	1 (2.8)	0 (0)	1 (1.4)
Primary school level	0 (0)	1 (2.6)	1 (1.4)
Secondary school level	0 (0)	4 (10.5)	4 (5.4)
High school level	0 (0)	7 (18.4)	7 (9.5)
Degree holder	9 (25.0)	6 (15.8)	15 (20.3)
Others	26 (72.2)	20 (52.6)	46 (62.2)
Current role of respondent, n (%)			
Malaria diagnosis, prevention & control	14 (38.9)	-na-	14 (18.9)
Malaria research & surveillance	10 (27.8)	-na-	10 (13.5)
Management role	9 (25.0)	-na-	9 (12.2)
Medical doctor	-na-	3 (7.9)	3 (4.1)
Nurse	-na-	3 (7.9)	3 (4.1)
Midwife	-na-	1 (2.6)	1 (1.4)
Village health worker	-na-	14 (36.8)	14 (18.9)
Health centre staff	-na-	10 (26.3)	10 (13.5)
Others	3 (8.3)	7 (18.4)	10 (13.5)
Years of service in current role, mean (SD)	15.9 (11.1)	14.1 (8.9)*	15.0 (10.0)

*Missing values present

Supplementary Table 2: Participants in qualitative data collection of Focus Group Discussions (FGDs) and semi-structured interviews

Methods	Province	# Participant	Remark
Semi-structured interview with health stakeholders/ staff	Phu Yen	16	Interview with district and provincial level staff
FGD with FHSPs	Phu Yen	16	4 participants / FGD x 4 FGDs
FGD with mobile migrant populations (MMPs)	Phu Yen	16	4 participants / FGD x 4 FGDs
Semi-structured interview with health stakeholders/ staff	Binh Thuan	12	Interview with district and provincial level staff
FGD with FHSPs	Binh Thuan	18	4-5 participants / FGD x 4 FGD
FGD with MMPs	Binh Thuan	20	5 participants / FGD x 4 FGD
Total	2 Provinces	98	28 interviews and 16 FGDs

Supplementary Table 3: Barriers for case investigation and reactive case detection (RACD) (Survey)

Information on case and foci investigations including RACD	Health stakeholders/ staff	FHSPs	Total
**Main reasons for cases that were not investigated*†, n (%)**			
Case was an imported case	1 (2.8)	8 (23.5)	9 (12.9)
Case was outside of the district of the person investigating	1 (2.8)	10 (29.4)	11 (15.7)
The person could not be found	7 (19.4)	18 (52.9)	25 (35.7)
Not enough staff/resources	2 (5.6)	2 (5.9)	4 (5.7)
Case was a daily cross-border case	3 (8.3)	5 (14.7)	8 (11.4)
Not applicable – every case is investigated	18 (50.0)	9 (5)	27 (38.6)
Others	4 (11.1)	2 (5.9)	6 (8.6)
**Challenges in conducting case investigation*†, n (%)**			
No challenge	10 (27.8)	9 (26.5)	19 (27.1)
Language barrier	3 (8.3)	1 (2.9)	4 (5.7)
Insufficient information from patient/uncooperative patient	3 (8.3)	10 (29.4)	13 (18.6)
Difficult or unable to contact/find patient	14 (38.9)	14 (41.2)	28 (40.0)
Insufficient funding	3 (8.3)	2 (5.9)	5 (7.1)
Insufficient manpower	4 (11.1)	1 (2.9)	5 (7.1)
Weather and transportation difficulties	2 (5.6)	8 (23.5)	10 (14.3)
**What was done if the index case was not home when visited for case investigation*†, n (%)**			
Visit a second time: later that day or on the day after	8 (22.9)	13 (34.2)	21 (28.8)
Telephone to schedule an appointment	18 (51.4)	24 (63.2)	42 (57.5)
Mark the case as ‘imported’	0 (0.0)	0 (0.0)	0 (0.0)
Mark the case as ‘not found’	1 (2.9)	1 (2.6)	2 (2.7)
Do not re-visit the index case	0 (0.0)	0 (0.0)	0 (0.0)
Inform volunteers to make appointment with the case	13 (37.1)	12 (31.6)	25 (34.2)
Others	3 (8.6)	5 (13.2)	8 (11.0)
**What is done if someone from the household of the index case is not home and they cannot be screened for RACD*†, n (%)**			
Visit the household later that day or on a subsequent day	8 (22.9)	23 (60.5)	31 (42.5)
Schedule an appointment with the household members to return	25 (71.4)	19 (50.0)	44 (60.3)
Do not return	1 (2.9)	3 (7.9)	4 (5.5)
Other	1 (2.9)	0 (0.0)	1 (1.4)
**Challenges in conducting RACD in the community*†, n (%)**			
No challenge	8 (25.8)	3 (10.0)	11 (18.0)
Language barrier	1 (3.2)	0 (0.0)	1 (1.6)

Insufficient information from patient/uncooperative patient	3 (9.7)	9 (30.0)	12 (19.7)
Difficult or unable to contact/find patient	7 (22.6)	16 (53.3)	23 (37.7)
Insufficient funding	9 (29.0)	1 (3.3)	10 (16.4)
Insufficient manpower	8 (25.8)	0 (0.0)	8 (13.1)
Weather and transportation difficulties	3 (9.7)	5 (16.7)	8 (13.1)

*Missing values present

†Multiple responses allowed

Supplementary figures

Supplementary figure 1: Health system structure in Vietnam and budget allocation for health

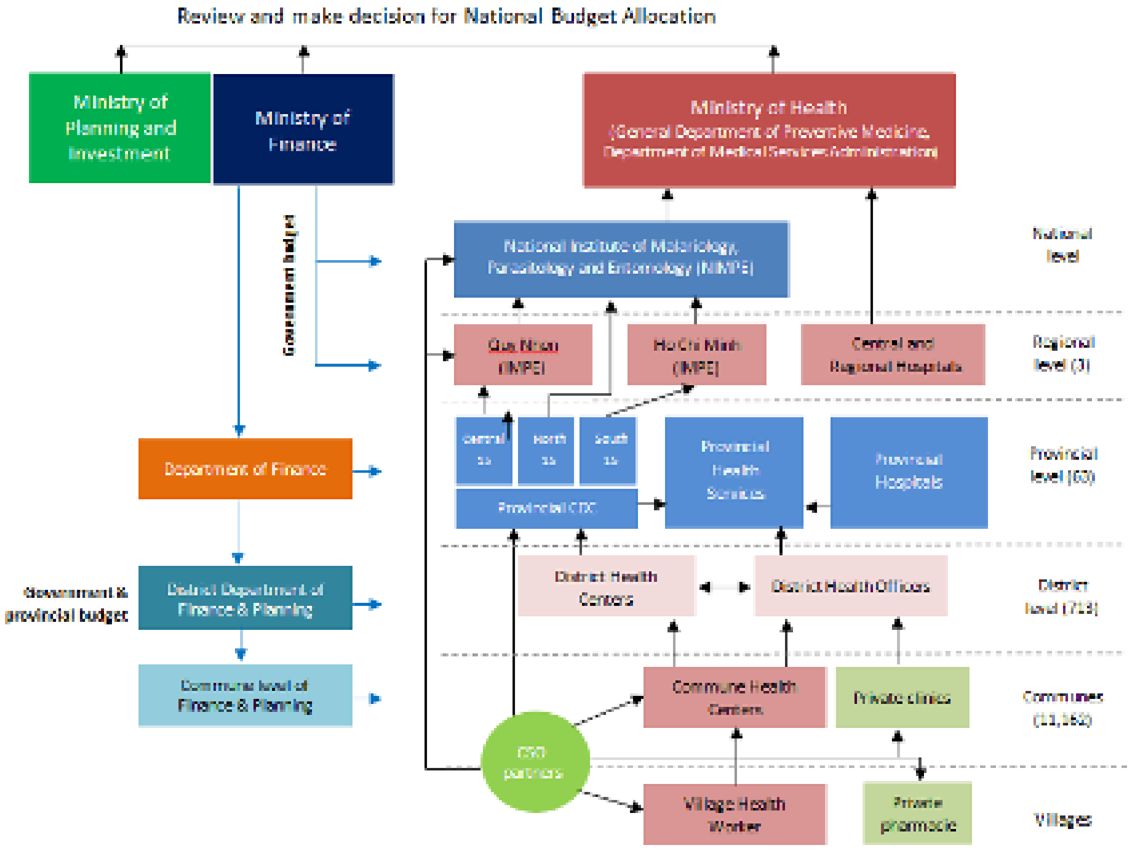

Adapted from Vietnam malaria transition and sustainability assessment report 2021 (Hanoi, Vietnam, page 35) by the Institute of Malariology Parasitology and Entomology and the University of California San Francisco Malaria Elimination Initiative.
