# Supplementary material for: Facilitators, barriers and acceptability of malaria reactive surveillance and response strategies in Vietnam: a mixed-methods study
Source: BMJ Public Health. 2024 Dec 16;2(2):e000961. doi: 10.1136/bmjph-2024-000961 (PMC11816204; doi:10.1136/bmjph-2024-000961)
Supplement: online supplemental file 5 [file bmjph-2-2-s005.pdf]

id	questionna	country	age_yr	sex	education	role	role_cat	totalservic
q1_01	Malaria pr	Vietnam	57	Male	Others	General m	Manageme	30
q1_02	Malaria pr	Vietnam	53	Male	Others	control the	Malaria dia	28
q1_03	Malaria pr	Vietnam	54	Male	Others	Head of inf	Manageme	30
q1_04	Malaria pr	Vietnam	31	Male	Degree ho	control the	Malaria dia	10
q1_05	Malaria pr	Vietnam	49	Male	Degree ho	Health staf	Others	22
q1_06	Malaria pr	Vietnam	30	Female	Others	Health staf	Others	1
q1_07	Malaria pr	Vietnam	44	Male	Others	Vice direct	Manageme	3
q1_08	Malaria pr	Vietnam	60	Male	Others	Control ma	Malaria dia	42
q1_09	Malaria pr	Vietnam	32	Male	Others	The head c	Manageme	13
q1_10	Malaria pr	Vietnam	43	Male	Others	Supervise t	Malaria dia	24
q1_11	Malaria pr	Vietnam	33	Female	Others	Malaria tes	Malaria dia	10
q1_12	Malaria pr	Vietnam	32	Female	Degree ho	Aggregate	Malaria res	11
q1_13	Malaria pr	Vietnam	42	Female	Others	Malaria tes	Malaria dia	20
q1_14	Malaria pr	Vietnam	32	Male	Degree ho	Malaria tes	Malaria dia	11
q1_15	Malaria pr	Vietnam	31	Female	Degree ho	Malaria tes	Malaria dia	10
q1_16	Malaria pr	Vietnam	30	Female	Others	Aggregate	Malaria res	10
q1_17	Malaria pr	Vietnam	33	Male	Degree ho	Statistical (	Malaria res	7
q1_18	Malaria pr	Vietnam	59	Male	Others	malaria ep	Malaria res	2
q1_19	Malaria pr	Vietnam	30	Male	Others	malaria ep	Malaria res	6
q1_20	Malaria pr	Vietnam	45	Male	Degree ho	malaria ep	Malaria res	22
q1_21	Malaria pr	Vietnam	54	Female	No formal	Malaria tes	Malaria dia	25
q1_22	Malaria pr	Vietnam	37	Male	Others	Malaria Sta	Malaria res	11
q1_23	Malaria pr	Vietnam	34	Female	Others	Malaria Sta	Malaria res	4
q1_24	Malaria pr	Vietnam	31	Male	Others	malaria res	Malaria res	5
q1_25	Malaria pr	Vietnam	40	Female	Others	malaria res	Malaria res	20
q1_26	Malaria pr	Vietnam	52	Male	Degree ho	In charge c	Manageme	5
q1_27	Malaria pr	Vietnam	49	Male	Degree ho	Manageme	Malaria dia	2
q1_28	Malaria pr	Vietnam	32	Male	Others	malaria pr	Malaria dia	11
q1_29	Malaria pr	Vietnam	53	Male	Others	malaria pr	Malaria dia	29
q1_30	Malaria pr	Vietnam	49	Male	Others	vice presid	Manageme	25
q1_31	Malaria pr	Vietnam	29	Female	Others	public heal	Manageme	4
q1_32	Malaria pr	Vietnam	32	Male	Others	Faculty of !	Others	4
q1_33	Malaria pr	Vietnam	53	Male	Others	Polyclinic t	Malaria dia	30
q1_34	Malaria pr	Vietnam	55	Male	Others	In charge c	Malaria dia	32
q1_35	Malaria pr	Vietnam	50	Male	Others	Head of De	Manageme	26
q1_36	Malaria pr	Vietnam	52	Male	Others	Vice presic	Manageme	28
q2_01	Frontline n	Vietnam	38	Male	High schoc	Nursing	Nurse	15
q2_02	Frontline n	Vietnam	38	Male	High schoc	Head of CH	Health cen	15
q2_03	Frontline n	Vietnam	49	Female	Degree ho	VHW	VHW	19
q2_04	Frontline n	Vietnam	49	Female	Degree ho	Assitant dc	Medical dc	27
q2_05	Frontline n	Vietnam	51	Male	Secondary	Vice head	Health cen	30
q2_06	Frontline n	Vietnam	49	Female	High schoc	Nursing	Nurse	20
q2_07	Frontline n	Vietnam	29	Male	Others	VHW	VHW	6
q2_08	Frontline n	Vietnam	28	Female	Others	Nursing	Nurse	5
q2_09	Frontline n	Vietnam	38	Male	Degree ho	Head of CH	Health cen	15
q2_10	Frontline n	Vietnam	41	Female	Others	midwives	Midwife	16
q2_11	Frontline n	Vietnam	28	Male	Secondary	VHW	VHW	1
q2_12	Frontline n	Vietnam	32	Male	High schoc	VHW	VHW	1
q2_13	Frontline n	Vietnam	31	Female	High schoc	VHW	VHW	12

q2_14	Frontline n	Vietnam	47	Male	Others	Head of CH	Health cen	29
q2_15	Frontline n	Vietnam	63	Male	Primary sc	VHW	VHW	10
q2_16	Frontline n	Vietnam	35	Female	Others	Health stat	Health cen	14
q2_17	Frontline n	Vietnam	40	Female	Others	Health stat	Health cen	14
q2_18	Frontline n	Vietnam	42	Female	Others	Malaria in	Others	19
q2_19	Frontline n	Vietnam	54	Female	Degree ho	VHW	VHW	14
q2_20	Frontline n	Vietnam	47	Female	Others	Head of CH	Health cen	26
q2_21	Frontline n	Vietnam	40	Female	Others	Health Car	Others	11
q2_22	Frontline n	Vietnam	52	Female	Others	CHS	Health cen	28
q2_23	Frontline n	Vietnam	55	Female	Others	VHW Than	VHW	
q2_24	Frontline n	Vietnam	34	Female	Degree ho	Pharmacy	Others	11
q2_25	Frontline n	Vietnam	37	Female	Others	Initial SK ca	Others	1
q2_26	Frontline n	Vietnam	40	Female	Others	VHW	VHW	23
q2_27	Frontline n	Vietnam	53	Female	Secondary	VHW Phuo	VHW	12
q2_28	Frontline n	Vietnam	53	Male	Others	Doctor	Medical do	26
q2_29	Frontline n	Vietnam	32	Female	Others	In charge c	Others	1
q2_30	Frontline n	Vietnam	24	Female	Others	Doctor	Medical do	1
q2_31	Frontline n	Vietnam	40	Male	High schoo	VHW	VHW	6
q2_32	Frontline n	Vietnam	37	Female	Others	In charge c	Others	15
q2_33	Frontline n	Vietnam	61	Female	Secondary	VHW	VHW	20
q2_34	Frontline n	Vietnam	31	Female	Others	Staff at Ph	Health cen	3
q2_35	Frontline n	Vietnam	41	Male	Others	In charge c	Others	20
q2_36	Frontline n	Vietnam	52	Male	Others	Head of CH	Health cen	24
q2_37	Frontline n	Vietnam	31	Male	High schoo	VHW	VHW	7
q2_38	Frontline n	Vietnam	28	Female	Degree ho	VHW	VHW	4

Malaria pr	Where this	How positi	Paper-base	Electronic	Telephone	Messaging	How frequ	Trigger for
Yes, other	All areas	2	No	Yes	No	No	Sometime	Case repor
Yes, other	All areas	1.3	Yes	No	Yes	No	Nearly alw	Other
Yes, 1-3-7	Areas in el	1.2	Yes	Yes	No	No	Often (50-	Other
Yes, 1-3-7	Areas in el	1.3	Yes	No	Yes	No	Nearly alw	Case repor
Yes, other	Areas in el	2	No	Yes	No	No	Nearly alw	Other
Yes, other		4	No	No	No	Yes	Nearly alw	Case repor
No	Areas in el	.					Occasional	Case reprt
Yes, 1-3-7	Areas in el	2	No	Yes	No	No	Nearly alw	Case repor
Yes, other	Others	1.2	Yes	Yes	No	No	Nearly alw	Case reprt
Yes, 1-3-7	Areas in el	1.4	Yes	No	No	Yes	Nearly alw	Case reprt
Yes, 1-3-7	All areas	2	No	Yes	No	No	Usually (<7	Other
Yes, 1-3-7	All areas	2	No	Yes	No	No	Nearly alw	Case repor
Yes, other	Areas in el	2	No	Yes	No	No	Nearly alw	Other
Yes, other	All areas	2	No	Yes	No	No	Nearly alw	Case repor
Yes, 1-3-7	Areas in el	3	No	No	Yes	No	Usually (<7	Case reprt
Yes, other	All areas	2	No	Yes	No	No	Nearly alw	Case repor
Yes, other	All areas	2	No	Yes	No	No	Nearly alw	Case repor
Yes, 1-3-7	All areas	4	No	No	No	Yes	Occasional	Case reprt
Yes, 1-3-7	All areas	.					Occasional	Case reprt
Yes, other	All areas	1.2	Yes	Yes	No	No	Usually (<7	Case reprt
Yes, 1-3-7	All areas	2.3	No	Yes	Yes	No	Often (50-	Other
Yes, 1-3-7	All areas	1.2	Yes	Yes	No	No	Usually (<7	Case reprt
Yes, 1-3-7	All areas	2	No	Yes	No	No	Usually (<7	Case repor
Yes, 1-3-7	All areas	2	No	Yes	No	No	Nearly alw	Case reprt
Yes, 1-3-7	All areas	2	No	Yes	No	No	Usually (<7	Other
Yes, other	All areas	2.3	No	Yes	Yes	No	Usually (<7	Other
Yes, other	Others	.					Occasional	Case reprt
Yes, 1-3-7	Areas in el	1	Yes	No	No	No	Nearly alw	Case repor
Yes, other	Areas in el	2	No	Yes	No	No	Nearly alw	Case repor
Yes, other	All areas	2.3	No	Yes	Yes	No	Nearly alw	Other
Yes, other	All areas	3	No	No	Yes	No	Usually (<7	Case repor
Yes, other	Others	2	No	Yes	No	No	Nearly alw	Case repor
Yes, other	All areas	3	No	No	Yes	No	Usually (<7	Case repor
Yes, 1-3-7	Areas in el	2	No	Yes	No	No	Nearly alw	Case repor
Yes, other	All areas	2	No	Yes	No	No	Nearly alw	Case repor
Yes, other	Others	2	No	Yes	No	No	Nearly alw	Case repor
Yes, 1-3-7		2	No	Yes	No	No	Usually (<7	Case reprt
Yes, 1-3-7	All areas	1.2	Yes	Yes	No	No	Nearly alw	Case repor
Yes, 1-3-7	Others	3	No	No	Yes	No	Nearly alw	Case repor
Yes, other	All areas	1	Yes	No	No	No	Often (50-	Case repor
Yes, 1-3-7	Areas in el	1	Yes	No	No	No	Never	Case repor
No	All areas	1	Yes	No	No	No	Sometime	Other
Yes, 1-3-7	All areas	1	Yes	No	No	No	Occasional	Case repor
Yes, 1-3-7	All areas	2	No	Yes	No	No	Nearly alw	Case repor
Yes, other	Areas in el	2	No	Yes	No	No	Often (50-	Case repor
Yes, 1-3-7	All areas	2	No	Yes	No	No	Nearly alw	Case repor
Yes, 1-3-7	Areas in el	2	No	Yes	No	No	Often (50-	Case repor
Yes, 1-3-7	All areas	3	No	No	Yes	No	Nearly alw	Case repor
Yes, other	Areas in el	3	No	No	Yes	No	Occasional	Other

Yes, 1-3-7	Areas in el	3	No	No	Yes	No	Nearly alw	Case repor
Yes, other	All areas	1	Yes	No	No	No	Occasional	Case repor
Yes, 1-3-7	Areas in el	2	No	Yes	No	No	Nearly alw	Case repor
Yes, 1-3-7	All areas	3	No	No	Yes	No	Nearly alw	Case repor
Yes, other	Areas in el	1	Yes	No	No	No	Nearly alw	Case repor
Yes, 1-3-7	All areas	3	No	No	Yes	No	Nearly alw	Case repor
Yes, other	All areas	1.3	Yes	No	Yes	No	Nearly alw	Case repor
Yes, other	All areas	1	Yes	No	No	No	Usually (<7	Case repor
Yes, other	Areas in el	1.3	Yes	No	Yes	No	Nearly alw	Case repor
	Don't knov	Mang đến	Yes	No	No	No	Usually (<7	Other
Yes, 1-3-7	All areas	3	No	No	Yes	No	Nearly alw	Case repor
Yes, other	All areas	2.3	No	Yes	Yes	No	Usually (<7	Case repor
Yes, other	Don't knov	3	No	No	Yes	No	Usually (<7	
Yes, 1-3-7	All areas	3	No	No	Yes	No	Nearly alw	Case repor
Yes, other	Others	3	No	No	Yes	No	Nearly alw	Case repor
Yes, other	Don't knov	2	No	Yes	No	No	Nearly alw	Case repor
Yes, 1-3-7	All areas	3	No	No	Yes	No	Nearly alw	Case repor
Yes, other	All areas	3	No	No	Yes	No	Nearly alw	Case repor
Yes, 1-3-7	Others	2	No	Yes	No	No	Nearly alw	Case repor
Yes, other	All areas	Báo cáo trı					Nearly alw	Case repor
		1	Yes	No	No	No	Usually (<7	Case repor
Yes, other	All areas	3	No	No	Yes	No	Nearly alw	Case repor
	Others	3	No	No	Yes	No	Occasional	Case repor
Yes, 1-3-7	All areas	3	No	No	Yes	No		Case repor
No	All areas	3	No	No	Yes	No	Nearly alw	Case repor

Policy for c	How frequ	Main reasc	Reason - ir	Reason - o	Reason - p	Reason - n	Reason - d	Reason - N
All indigen	For all case	6	No	No	No	No	No	Yes
Indigenous	For all case	6	No	No	No	No	No	Yes
Indigenous	For all case	6	No	No	No	No	No	Yes
Indigenous	For all case	6	No	No	No	No	No	Yes
All indigen	For all case	6	No	No	No	No	No	Yes
Indigenous	More than	4	No	No	No	Yes	No	No
All indigen	More than	6	No	No	No	No	No	Yes
All indigen	For all case	6	No	No	No	No	No	Yes
All indigen	More than	6	No	No	No	No	No	Yes
All indigen	More than	7	No	No	No	No	No	No
All indigen	For all case	3	No	No	Yes	No	No	No
Indigenous	More than	5	No	No	No	No	Yes	No
Indigenous	For all case	3	No	No	Yes	No	No	No
All indigen	For all case	4	No	No	No	Yes	No	No
Imported c	Between 2	5	No	No	No	No	Yes	No
Indigenous	For all case	3	No	No	Yes	No	No	No
All indigen	For all case	6	No	No	No	No	No	Yes
All indigen	For all case	6	No	No	No	No	No	Yes
All indigen	For all case	2	No	Yes	No	No	No	No
All indigen	For all case	6	No	No	No	No	No	Yes
All indigen	For all case	3	No	No	Yes	No	No	No
All indigen	For all case	3	No	No	Yes	No	No	No
All indigen	For all case	6	No	No	No	No	No	Yes
All indigen	More than	6	No	No	No	No	No	Yes
All indigen	Between 2	5	No	No	No	No	Yes	No
Others	For all case	6	No	No	No	No	No	Yes
		1	Yes	No	No	No	No	No
All indigen	For all case	7	No	No	No	No	No	No
All indigen	For all case	6	No	No	No	No	No	Yes
All indigen	For all case	6	No	No	No	No	No	Yes
All indigen	For all case	6	No	No	No	No	No	Yes
Indigenous	For all case	3	No	No	Yes	No	No	No
All indigen	For all case	3	No	No	Yes	No	No	No
All indigen	For all case	7	No	No	No	No	No	No
All indigen	For all case	6	No	No	No	No	No	Yes
Indigenous	For all case	7	No	No	No	No	No	No
All indigen	For all case	1,2,3,5,6	Yes	Yes	Yes	No	Yes	Yes
All indigen	For all case	3	No	No	Yes	No	No	No
Indigenous	For all case	1.2	Yes	Yes	No	No	No	No
All indigen	For all case	3	No	No	Yes	No	No	No
All indigen	For all case	3	No	No	Yes	No	No	No
All indigen	More than	2,5,6	No	Yes	No	No	Yes	Yes
Don't knov	More than	1,2,3	Yes	Yes	Yes	No	No	No
All indigen	More than	1,2	Yes	Yes	No	No	No	No
All indigen	For all case	2.3.5	No	Yes	Yes	No	Yes	No
All indigen	For all case	1.3	Yes	No	Yes	No	No	No
All indigen	More than	3	No	No	Yes	No	No	No
All indigen	More than	1,2,3	Yes	Yes	Yes	No	No	No
All indigen	For all case	3	No	No	Yes	No	No	No

Indigenous	For all case	1	Yes	No	No	No	No	No
All indigen	More than	1,2,6	Yes	Yes	No	No	No	Yes
All indigen	For all case	3	No	No	Yes	No	No	No
All indigen	Between 5	3	No	No	Yes	No	No	No
All indigen	More than	6	No	No	No	No	No	Yes
Indigenous	More than	6	No	No	No	No	No	Yes
All indigen	For all case	7: Điều tra	No	No	No	No	No	No
Don't know	Between 5	4	No	No	No	Yes	No	No
All indigen	More than	6	No	No	No	No	No	Yes
Indigenous	For all case	.						
All indigen	More than	6	No	No	No	No	No	Yes
All indigen	For all case	.						
	More than	3	No	No	Yes	No	No	No
All indigen	More than	5	No	No	No	No	Yes	No
All indigen	For all case	7. Điều tra	No	No	No	No	No	No
All indigen	For all case	.						
Indigenous	More than	6	No	No	No	No	No	Yes
All indigen	Between 5	4	No	No	No	Yes	No	No
All indigen	For all case	2.3	No	Yes	Yes	No	No	No
All indigen	For all case	3	No	No	Yes	No	No	No
Indigenous	For all case	3	No	No	Yes	No	No	No
Indigenous	For all case	.						
All indigen	For all case	6	No	No	No	No	No	Yes
All indigen	Between 5	3	No	No	Yes	No	No	No
Indigenous	More than	2,3,5	No	Yes	Yes	No	Yes	No

Other reas	What occu	q1_10	Specific pe	Responsibl	Whether tl	Frequency	Personnel	Frequency
No	Don’t knov	2	No	Others	Yes	Yearly	Yes	Quarterly
No	It maybe h	1	Yes	Others	Yes	Yearly	Yes	Quarterly
No	It is very d	1: The staf	Yes	Others	Yes	Yearly	Yes	Other
No	No	1	Yes	Others	Yes	Yearly	Yes	Quarterly
No	no	1	Yes	Others	Yes	Yearly	Yes	Quarterly
No	no	1	Yes	Others	No	Quarterly	Yes	Quarterly
No	No	1	Yes	Others	Yes	Yearly	Yes	Quarterly
No	Consequer	1	Yes	VHV or eq	No	Monthly	No	Quarterly
No	No	1: the mal	Yes	Others	Yes	Yearly	Yes	Quarterly
Yes	No affect a	1	Yes	Others		Monthly		Monthly
No	not availab	1	Yes	Others	Yes	Yearly	Yes	Yearly
No	disease sp	1	Yes	VHV or eq	Yes	Every seco	Yes	Monthly
No	Spread in t	1	Yes	Others	Yes	Every seco	Yes	Monthly
No	DON'T KN	1	Yes	Others	Yes	Every seco	Yes	Quarterly
No	will spread	1	Yes	VHV or eq	Yes	Yearly	Yes	Yearly
No	The source	1	Yes	Others	Yes	Yearly	Yes	Monthly
No	No	1	Yes	Others	Yes	Yearly	Yes	Yearly
No	Spread of	1	Yes	Others	Yes	Yearly	Yes	Yearly
No	making it c	1	Yes	VHV or eq	Yes	Monthly	Yes	Monthly
No	spread ma	1	Yes	Others	Yes	Quarterly	No	Quarterly
No	inadequat	1	Yes	VHV or eq	Yes	Yearly	Yes	Monthly
No	Spread in t	1	Yes	Others	Yes	Yearly	Yes	Other
No	Spread in t	1	Yes	Others	Yes	Yearly	Yes	Other
No	Spread in t	2	No	Others	Yes	Every seco	Yes	Quarterly
No	spread int	1	Yes	VHV or eq	Yes	Quarterly	Yes	Quarterly
No	Severe ma	1	Yes	VHV or eq	Yes	Quarterly	Yes	Quarterly
No	Spread in t	1	Yes	Others	Yes	Yearly	Yes	Quarterly
Yes	no effect	1	Yes	Others	Yes	Every seco	Yes	Quarterly
No	no effect	1	Yes	Others	Yes	Every seco	Yes	Other
No	no effect	1	Yes	Others	Yes	Yearly	Yes	Quarterly
No	no effect	1	Yes	Others	Yes	Yearly	Yes	Other
No	Risk of spr	1	Yes	Others	Yes	Every seco	Yes	Other
No	The risk of	1	Yes	Others	Don't knov		Yes	Yearly
Yes	Taken to tl	1	Yes	Others	Yes	Yearly	Yes	Quarterly
No	.	1	Yes	Others	Yes	Yearly	Yes	Quarterly
Yes	Late detec	1	Yes	Others	Yes	Every seco	Yes	Other
No	Patient no	.						Monthly
No		.						Monthly
No		.						Monthly
No	They go to	.						Monthly
No		.						Monthly
No	infect to o	.						Monthly
No	The malari	.						Monthly
No	Outbreak a	.						Quarterly
No	there is th	.						Monthly
No	it is a outb	.						Monthly
No	If we don’t	.						Monthly
No	if we don’t	.						Quarterly
No	It could be	.						Monthly

No		.	Monthly
No		.	Other
No	No drug re	.	Monthly
No	infect to ot	.	Quarterly
No	infect to ot	.	Yearly
No		.	Monthly
Yes	Severe pat	.	Other
No	Don't knov	.	Yearly
No		.	Quarterly
	Reduces he	.	Other
No	.	.	Other
	.	.	Other
No	Life-threat	.	Monthly
No	.	.	Yearly
Yes	Spread dis	.	Other
	No investig	.	Yearly
No	.	.	Yearly
No	.	.	Yearly
No	No new ca	.	Other
No	100% inves	.	Yearly
No	Failure to r	.	Other
	Undetecte	.	Yearly
No	.	.	
No	Spread dis	.	
No	Report to t	.	Other

How soon	Presence c	Presence c	CI involves	Method of	What is do	If index ca	If index ca	If index ca
Within 48	Yes	Yes		Telephone	6	No	No	No
Within 72	Yes	Yes	Yes, alway	Telephone	6	No	No	No
Within one	Yes	Yes	Yes, somet	Telephone	2	No	Yes	No
Within 48	Yes	Yes		Telephone	2	No	Yes	No
Within 48	Yes	Yes	Yes, alway	Other	1	Yes	No	No
Within 48	Yes	Yes	Yes, alway	Telephone	6	No	No	No
Within 48	Yes	Yes	Yes, alway	Telephone	2	No	Yes	No
Within 24	Yes	Yes	Yes, alway	Telephone	1	Yes	No	No
Within 72	Yes	Yes	Yes, alway	Telephone	6	No	No	No
Within 48	Yes	Yes	Yes, alway	No prior cc	7	No	No	No
Within 72	Yes	Yes	Yes, alway	Telephone	4.6	No	No	No
Within 72	Yes	Yes	Yes, somet	Telephone	6	No	No	No
Within 48	Yes	Yes	Yes, alway	No prior cc	6	No	No	No
Within 24	Yes	Yes	Yes, alway	Telephone	6	No	No	No
Within one	Yes	Yes	Yes, somet	Telephone	1	Yes	No	No
Within 72	Yes	Yes	Yes, alway	Telephone	1.2	Yes	Yes	No
Within 48	Yes	Yes	Yes, alway	Telephone	1.2	Yes	Yes	No
Within 48	Yes	Yes	Yes, alway	Telephone	2	No	Yes	No
Within 48	Yes	Yes	Yes, alway	Telephone	2	No	Yes	No
Within 48	Yes	Yes	Yes, alway	Telephone	2.6	No	Yes	No
Within 72	Yes	Yes	Yes, alway	Other	6	No	No	No
Within 48	Yes	Yes	Yes, alway	Telephone	2	No	Yes	No
Within 48	Yes	Yes	Yes, alway	No prior cc	6	No	No	No
Within 72	Yes	Yes	Yes, somet	Telephone	2	No	Yes	No
Within one	Yes	Yes	Yes, somet	Telephone	6	No	No	No
Within 72	Yes	Yes	Yes, somet	Telephone	2	No	Yes	No
Within 72	Yes	Yes	Yes, alway	Other	1.2	Yes	Yes	No
Within 72	Yes	Yes	Yes, alway	Telephone	2	No	Yes	No
Within 72	Yes	Yes	Yes, alway	Telephone	1,2,6	Yes	Yes	No
Within 48	Yes	Yes	Yes, alway	No prior cc	7	No	No	No
Within 72	Yes	Yes	Yes, alway	Telephone	2.7	No	Yes	No
Within 72	Yes	Yes	Yes, alway	Telephone	2	No	Yes	No
Within 48	Yes	Yes	No, never		.			
Within 48	Yes	Yes	Yes, somet	Telephone	2	No	Yes	No
Within 24	Yes	Yes	Yes, somet	Telephone	1	Yes	No	No
Within 72	Yes	Yes	Yes, alway	Telephone	2	No	Yes	No
Within one	Yes	Yes	Yes, alway	Telephone	1,2,6	Yes	Yes	No
Within 72	Yes	Yes	Yes, alway	Telephone	2	No	Yes	No
Within 72	Yes	Yes	Yes, alway	No prior cc	6	No	No	No
Within one	Yes	Yes	Yes, alway	No prior cc	1.2	Yes	Yes	No
Within one	Yes	Yes	Yes, alway	Telephone	2	No	Yes	No
Within 24	Yes	No		Telephone	1	Yes	No	No
Within 72	Yes	Yes	Yes, alway	No prior cc	1,2,6	Yes	Yes	No
Within 72	Yes	Yes	Yes, alway	Telephone	1	Yes	No	No
Within 72	Yes	Yes	Yes, alway	Telephone	2	No	Yes	No
Within 72	Yes	Yes	Yes, alway	Telephone	6	No	No	No
Within 48	Yes	Yes	Yes, alway	Telephone	1	Yes	No	No
Within 72	Yes	Yes	Yes, alway	Telephone	1.2	Yes	Yes	No
Within 72	Yes	Yes	Yes, alway	Telephone	2	No	Yes	No

Within 48	Yes	Yes	Yes, alway	Other	2	No	Yes	No
Within 48	Yes	Yes	Yes, alway	No prior cc	6	No	No	No
Within 24	Yes	Yes	Yes, alway	Telephone	6	No	No	No
Within 72	No	Yes	Yes, alway	Telephone	2	No	Yes	No
Within 48	Yes	Yes	Yes, alway	Other	2	No	Yes	No
Within 24	Yes	Yes	Yes, alway	Telephone	2	No	Yes	No
Within 24	Yes	Yes	Yes, alway	Telephone	1,2,6	Yes	Yes	No
Within 48	No	No	No, never	Telephone	1	Yes	No	No
Within 72	Yes	Yes	Yes, alway	Telephone	1.2	Yes	Yes	No
Within 72	Yes	Yes	Yes, alway	Telephone	2	No	Yes	No
Within 24	Yes	Yes	Yes, alway	Other	2.7	No	Yes	No
Within 24	No	Yes	Yes, alway	Telephone	7	No	No	No
Within one	Yes	Yes	Yes, alway	No prior cc	7	No	No	No
Within 24	Yes	Yes	Yes, alway	Other	2.4	No	Yes	No
Within one	Yes	Yes	Yes, alway	Other	1,2,6	Yes	Yes	No
Within one	Yes	Yes	Yes, alway	Other	6	No	No	No
Within 24	Yes	Yes	Yes, alway	Telephone	2	No	Yes	No
Within 24	Yes	Yes	Yes, alway	Telephone	1.2	Yes	Yes	No
Within 72	Yes	Yes	Yes, alway	Telephone	2.6	No	Yes	No
Within 72	No	Yes	Yes, alway	Other	7	No	No	No
Within 24	Yes	Yes	Yes, alway	Telephone	1,2,6	Yes	Yes	No
Within one	Yes	Yes	Yes, somet	Other	2	No	Yes	No
Within 24	Yes	Yes	Yes, alway	Telephone	6	No	No	No
Within 24	Yes	No	Yes, alway	Other	2	No	Yes	No
Within 72	Yes	Yes	Yes, alway	No prior cc	7	No	No	No

If index cas	If index cas	If index cas	If index cas	Time of da	CI normally	CI normally	CI normally	CI normally
No	No	Yes	No	2	No	Yes	No	No
No	No	Yes	No	2	No	Yes	No	No
No	No	No	No	2.5	No	Yes	No	No
No	No	No	No	2	No	Yes	No	No
No	No	No	No	4	No	No	No	Yes
No	No	Yes	No	3	No	No	Yes	No
No	No	No	No	2	No	Yes	No	No
No	No	No	No	5	No	No	No	No
No	No	Yes	No	5	No	No	No	No
No	No	No	Yes	4.5	No	No	No	Yes
Yes	No	Yes	No	1.7	Yes	No	No	No
No	No	Yes	No	1	Yes	No	No	No
No	No	Yes	No	1	Yes	No	No	No
No	No	Yes	No	1	Yes	No	No	No
No	No	No	No	1	Yes	No	No	No
No	No	No	No	1	Yes	No	No	No
No	No	No	No	1.7	Yes	No	No	No
No	No	No	No	1	Yes	No	No	No
No	No	No	No	2,5,6	No	Yes	No	No
No	No	Yes	No	2.5	No	Yes	No	No
No	No	Yes	No	7	No	No	No	No
No	No	No	No	1	Yes	No	No	No
No	No	Yes	No	5.7	No	No	No	No
No	No	No	No	1	Yes	No	No	No
No	No	Yes	No	1	Yes	No	No	No
No	No	No	No	2	No	Yes	No	No
No	No	No	No	3	No	No	Yes	No
No	No	No	No	3	No	No	Yes	No
No	No	Yes	No	1.7	Yes	No	No	No
No	No	No	Yes	7	No	No	No	No
No	No	No	Yes	3	No	No	Yes	No
No	No	No	No	1.5	Yes	No	No	No
				.				
No	No	No	No	4	No	No	No	Yes
No	No	No	No	3	No	No	Yes	No
No	No	No	No	1	Yes	No	No	No
No	No	Yes	No	1,2,7	Yes	Yes	No	No
No	No	No	No	5	No	No	No	No
No	No	Yes	No	5	No	No	No	No
No	No	No	No	1,5,6	Yes	No	No	No
No	No	No	No	2	No	Yes	No	No
No	No	No	No	1	Yes	No	No	No
No	No	Yes	No	5	No	No	No	No
No	No	No	No	1	Yes	No	No	No
No	No	No	No	1	Yes	No	No	No
No	No	Yes	No	1	Yes	No	No	No
No	No	No	No	1	Yes	No	No	No
No	No	No	No	1.5	Yes	No	No	No
No	No	No	No	2	No	Yes	No	No

No	No	No	No	1	Yes	No	No	No
No	No	Yes	No	1,5,6	Yes	No	No	No
No	No	Yes	No	1	Yes	No	No	No
No	No	No	No	7	No	No	No	No
No	No	No	No	2	No	Yes	No	No
No	No	No	No	7	No	No	No	No
No	No	Yes	No	7	No	No	No	No
No	No	No	No	.				
No	No	No	No	3	No	No	Yes	No
No	No	No	No	3.4	No	No	Yes	Yes
No	No	No	Yes	2.7	No	Yes	No	No
No	No	No	Yes	7	No	No	No	No
No	No	No	Yes	7	No	No	No	No
Yes	No	No	No	7	No	No	No	No
No	No	Yes	No	1.5	Yes	No	No	No
No	No	Yes	No	5	No	No	No	No
No	No	No	No	7	No	No	No	No
No	No	No	No	3	No	No	Yes	No
No	No	Yes	No	1.5	Yes	No	No	No
No	No	No	Yes	2.5	No	Yes	No	No
No	No	Yes	No	2.5	No	Yes	No	No
No	No	No	No	2	No	Yes	No	No
No	No	Yes	No	1	Yes	No	No	No
No	No	No	No	4	No	No	No	Yes
No	No	No	Yes	3.7	No	No	Yes	No

CI normall	CI normall	CI normall	Main reasc	Reason - ir	Reason - o	Reason - p	Reason - n	Reason - d
No	No	No	6	No	No	No	No	No
No	No	No	6	No	No	No	No	No
Yes	No	No	3.7	No	No	Yes	No	No
No	No	No	6	No	No	No	No	No
No	No	No	3.5	No	No	Yes	No	Yes
No	No	No	4	No	No	No	Yes	No
No	No	No	6	No	No	No	No	No
Yes	No	No	6	No	No	No	No	No
Yes	No	No	6	No	No	No	No	No
Yes	No	No	7	No	No	No	No	No
No	No	Yes	1.3	Yes	No	Yes	No	No
No	No	No	3	No	No	Yes	No	No
No	No	No	3	No	No	Yes	No	No
No	No	No	3	No	No	Yes	No	No
No	No	No	3	No	No	Yes	No	No
No	No	No	1,2,3,5	Yes	Yes	Yes	No	Yes
No	No	Yes	3	No	No	Yes	No	No
No	No	No	1,2,3,4,5	Yes	Yes	Yes	Yes	Yes
Yes	Yes	No	2.3	No	Yes	Yes	No	No
Yes	No	No	3	No	No	Yes	No	No
No	No	Yes	1,2,3	Yes	Yes	Yes	No	No
No	No	No	3	No	No	Yes	No	No
Yes	No	Yes	4	No	No	No	Yes	No
No	No	No	3	No	No	Yes	No	No
No	No	No	1.3	Yes	No	Yes	No	No
No	No	No	7	No	No	No	No	No
No	No	No	6	No	No	No	No	No
No	No	No	7	No	No	No	No	No
No	No	Yes	7	No	No	No	No	No
No	No	Yes	6	No	No	No	No	No
No	No	No	6	No	No	No	No	No
Yes	No	No	1,2,3	Yes	Yes	Yes	No	No
			.					
No	No	No	3,7. BN sốt	No	No	Yes	No	No
No	No	No	6	No	No	No	No	No
No	No	No	1,3,7. do tl	Yes	No	Yes	No	No
No	No	Yes	6	No	No	No	No	No
Yes	No	No	3	No	No	Yes	No	No
Yes	No	No	1	Yes	No	No	No	No
Yes	Yes	No	1,2,6	Yes	Yes	No	No	No
No	No	No	1	Yes	No	No	No	No
No	No	No	2,3,5	No	Yes	Yes	No	Yes
Yes	No	No	1	Yes	No	No	No	No
No	No	No	1	Yes	No	No	No	No
No	No	No	3	No	No	Yes	No	No
No	No	No	1	Yes	No	No	No	No
No	No	No	7	No	No	No	No	No
Yes	No	No	1.3	Yes	No	Yes	No	No
No	No	No	7	No	No	No	No	No

No	No	No	6	No	No	No	No	No
Yes	Yes	No	1.4	Yes	No	No	Yes	No
No	No	No	3	No	No	Yes	No	No
No	No	Yes	3	No	No	Yes	No	No
No	No	No	6	No	No	No	No	No
No	No	Yes	6	No	No	No	No	No
No	No	Yes	3	No	No	Yes	No	No
			7	No	No	No	No	No
No	No	No	6	No	No	No	No	No
No	No	No	2	No	Yes	No	No	No
No	No	Yes	3	No	No	Yes	No	No
No	No	Yes	.					
No	No	Yes	3	No	No	Yes	No	No
No	No	Yes	6	No	No	No	No	No
Yes	No	No	2.3	No	Yes	Yes	No	No
Yes	No	No	1.3	Yes	No	Yes	No	No
No	No	Yes	6	No	No	No	No	No
No	No	No	1	Yes	No	No	No	No
Yes	No	No	3	No	No	Yes	No	No
Yes	No	No	7	No	No	No	No	No
Yes	No	No	3	No	No	Yes	No	No
No	No	No	7	No	No	No	No	No
No	No	No	6	No	No	No	No	No
No	No	No	4	No	No	No	Yes	No
No	No	Yes	2	No	Yes	No	No	No

Reason - N	Other reas	q1_24	Supervisec	q1_25	Follow-up	q1_26	CI involves	q1_27
Yes	No	Yes	Yes	Yes	Yes	Yes	Yes	No
Yes	No	no	No	no	No	yes	Yes	yes
No	Yes	No	No	no	No	yes	Yes	yes
Yes	No	Yes	Yes	Yes	Yes	Yes	Yes	Yes
No	No	Yes, but it	Yes	Yes, at the	Yes	yes	Yes	yes
No	No	Yes	Yes	Yes	Yes	Yes	Yes	yes
Yes	No	Yes, we tel	Yes	Yes	Yes	Yes, IEC foı	Yes	Yes
Yes	No	Yes	Yes	Yes	Yes	Yes; Takinբ	Yes	Yes
Yes	No	Yes	Yes	Yes	Yes	Yes	Yes	Yes
No	Yes	Yes	Yes	Yes	Yes	Yes	Yes	yes
No	No	Yes	Yes	Yes	Yes	có, vẫn thụ	Yes	Yes
No	No	Yes	Yes	yes	Yes	Yes	Yes	Yes
No	No	Yes	Yes	Yes	Yes	Yes	Yes	Yes
No	No	Yes	Yes	yes	Yes	yes	Yes	yes
No	No	Yes	Yes	yes	Yes	yes	Yes	yes
No	No	Yes	Yes	Yes	Yes	Yes	Yes	Yes
No	No	No	No	No	No	Yes	Yes	Yes
No	No	yes	Yes	yes	Yes	yes	Yes	yes
No	No	Yes	Yes	yes	Yes	yes	Yes	yes
No	No	yes	Yes	yes	Yes	yes	Yes	yes
No	No	yes	Yes	yes	Yes	no	No	yes
No	No	yes	Yes	yes	Yes	yes	Yes	yes
No	No	yes	Yes	yes	Yes	yes	Yes	yes
No	No	no	No	yes	Yes	yes	Yes	yes
No	No	yes	Yes	yes	Yes	yes	Yes	yes
No	Yes	Yes	Yes	Yes	Yes	Yes	Yes	Yes
Yes	No	Yes	Yes	yes	Yes	yes	Yes	yes
No	Yes	yes	Yes	yes	Yes	yes	Yes	yes
No	Yes	yes	Yes	yes	Yes	yes	Yes	yes
Yes	No	yes	Yes	yes	Yes	yes	Yes	yes
Yes	No	yes	Yes	yes	Yes	yes	Yes	yes
No	No	yes	Yes	yes	Yes	yes	Yes	yes
				yes	Yes	yes	Yes	yes
No	Yes	no	No	yes	Yes	yes	Yes	yes
Yes	No	yes	Yes	yes	Yes	yes	Yes	yes
No	Yes	yes	Yes	yes	Yes	yes	Yes	yes
Yes	No	Always	Yes	Always	Yes	using the r	Yes	about reas
No	No	Always	Yes	Always	Yes	Yes	Yes	Yes
No	No	Always	Yes	Always	Yes	Yes	Yes	Yes
Yes	No	Always	Yes	Always	Yes	Checking t	Yes	Bring the II
No	No	Always	Yes	Always	Yes	Yes	Yes	Yes
No	No	Always	Yes	Always	Yes	.		.
No	No	Always	Yes	Always	Yes	Yes	Yes	Yes
No	No	Always	Yes	Always	Yes	Yes	Yes	Yes
No	No	Always	Yes	Always	Yes	yes, check	Yes	Yes, becau
No	No	Always	Yes	Always	Yes	Yes	Yes	Yes
No	Yes	Always	Yes	Always	Yes	yes	Yes	yes
No	No	Always	Yes	Always	Yes	Yes, we ch	Yes	Yes. We cc
No	Yes	Always	Yes	Always	Yes	Yes	Yes	Yes

Yes	No	Always	Yes	Always	Yes	Yes	Yes	Yes
No	No	Always	Yes	Sometime:	Yes	Yes	Yes	Yes
No	No	Always	Yes	Always	Yes	Yes	Yes	yes
No	No	Always	Yes	Always	Yes	Yes	Yes	Yes, IEC
Yes	No	Always	Yes	Always	Yes	yes	Yes	yes, iec in
Yes	No	Never	No	Always	Yes	Yes	Yes	Yes
No	No	Always	Yes	Always	Yes	Yes	Yes	Yes
No	Yes	Always	Yes	Always	Yes	Yes	Yes	Yes
Yes	No	Always	Yes	Always	Yes	Yes	Yes	Yes
No	No	Always	Yes	Always	Yes	Coordinate	Yes	No
No	No	Always	Yes	Always	Yes	Yes, hand	Yes	Have
		Always	Yes	Always	Yes	Ask if you	Yes	Yes: Instru
No	No	Always	Yes	Always	Yes	Have	Yes	Have
Yes	No	Never	No	Always	Yes	Have	Yes	Have
No	No	Always	Yes	Always	Yes	Have	Yes	Have
No	No	Always	Yes	Always	Yes	Sleep unde	Yes	Propagand
Yes	No	Never	No	Always	Yes	Have	Yes	Have
No	No	Always	Yes	Never	No	Have	Yes	Have
No	No	Always	Yes	Always	Yes	Have	Yes	Have
No	Yes	Always	Yes	.		Ask where	Yes	Media: The
No	No	Always	Yes	Always	Yes	Have	Yes	Have
No	Yes	Always	Yes	Always	Yes	Do you sle	Yes	Media: Wh
Yes	No	Always	Yes	Sometime:	Yes	Have	Yes	Have
No	No	Always	Yes	Sometime:	Yes	Have	Yes	Have
No	No	Never	No	Never	No	Have	Yes	Have

CI involves	Informatio	Info used -	Info used -	Info used -	Info used -	Info used -	Info used -	Info used -
No	7	No	No	No	No	No	No	Yes
Yes	1,2,3	Yes	Yes	Yes	No	No	No	No
Yes	1	Yes	No	No	No	No	No	No
Yes	1,2,3,5	Yes	Yes	Yes	No	Yes	No	No
Yes	2	No	Yes	No	No	No	No	No
Yes	1	Yes	No	No	No	No	No	No
Yes	1,2,3,5	Yes	Yes	Yes	No	Yes	No	No
Yes	6	No	No	No	No	No	Yes	No
Yes	3	No	No	Yes	No	No	No	No
Yes	1	Yes	No	No	No	No	No	No
Yes	1,2,3,4,5	Yes	Yes	Yes	Yes	Yes	No	No
Yes	3	No	No	Yes	No	No	No	No
Yes	6	No	No	No	No	No	Yes	No
Yes	1	Yes	No	No	No	No	No	No
Yes	1	Yes	No	No	No	No	No	No
Yes	6	No	No	No	No	No	Yes	No
Yes	6	No	No	No	No	No	Yes	No
Yes	1,2,3,4,5,6	Yes	Yes	Yes	Yes	Yes	Yes	No
Yes	1,2,3	Yes	Yes	Yes	No	No	No	No
Yes	1.6	Yes	No	No	No	No	Yes	No
Yes	1,2,3,4,5	Yes	Yes	Yes	Yes	Yes	No	No
Yes	6	No	No	No	No	No	Yes	No
Yes	6	No	No	No	No	No	Yes	No
Yes	3,4,5	No	No	Yes	Yes	Yes	No	No
Yes	1	Yes	No	No	No	No	No	No
Yes	1,2,3,4,5	Yes	Yes	Yes	Yes	Yes	No	No
Yes	6	No	No	No	No	No	Yes	No
Yes	1	Yes	No	No	No	No	No	No
Yes	1,2,3	Yes	Yes	Yes	No	No	No	No
Yes	2	No	Yes	No	No	No	No	No
Yes	1,2,3,5	Yes	Yes	Yes	No	Yes	No	No
Yes	1	Yes	No	No	No	No	No	No
Yes	.							
Yes	2,3,4	No	Yes	Yes	Yes	No	No	No
Yes	7	No	No	No	No	No	No	Yes
Yes	1	Yes	No	No	No	No	No	No
Yes	1,2,3	Yes	Yes	Yes	No	No	No	No
Yes	3	No	No	Yes	No	No	No	No
Yes	6	No	No	No	No	No	Yes	No
Yes	1,2,3	Yes	Yes	Yes	No	No	No	No
Yes	3	No	No	Yes	No	No	No	No
	1	Yes	No	No	No	No	No	No
Yes	6	No	No	No	No	No	Yes	No
Yes	6	No	No	No	No	No	Yes	No
Yes	6	No	No	No	No	No	Yes	No
Yes	6	No	No	No	No	No	Yes	No
Yes	6	No	No	No	No	No	Yes	No
Yes	6	No	No	No	No	No	Yes	No
Yes	1.4	Yes	No	No	Yes	No	No	No

Yes	6	No	No	No	No	No	Yes	No
Yes	1,2,3	Yes	Yes	Yes	No	No	No	No
Yes	2	No	Yes	No	No	No	No	No
Yes	6	No	No	No	No	No	Yes	No
Yes	1,2,3	Yes	Yes	Yes	No	No	No	No
Yes	1,2,3,4,5	Yes	Yes	Yes	Yes	Yes	No	No
Yes	1	Yes	No	No	No	No	No	No
Yes	6	No	No	No	No	No	Yes	No
Yes	1,2,3,4	Yes	Yes	Yes	Yes	No	No	No
No	1	Yes	No	No	No	No	No	No
Yes	1	Yes	No	No	No	No	No	No
Yes	1,2,3,4,5	Yes	Yes	Yes	Yes	Yes	No	No
Yes	1,2,3	Yes	Yes	Yes	No	No	No	No
Yes	2,3,4,5	No	Yes	Yes	Yes	Yes	No	No
Yes	1	Yes	No	No	No	No	No	No
Yes	1,2,3	Yes	Yes	Yes	No	No	No	No
Yes	2,3,4,5	No	Yes	Yes	Yes	Yes	No	No
Yes	6	No	No	No	No	No	Yes	No
Yes	1	Yes	No	No	No	No	No	No
Yes	1,2,3	Yes	Yes	Yes	No	No	No	No
Yes	1	Yes	No	No	No	No	No	No
Yes	1,2,3,4	Yes	Yes	Yes	Yes	No	No	No
Yes	1,2,3	Yes	Yes	Yes	No	No	No	No
Yes	7	No	No	No	No	No	No	Yes
Yes	1	Yes	No	No	No	No	No	No

CI involves	CI involves	Collect tra	Collect tra	Collect tra	Collect tra	How impo	Collect dat	Challenges
No	No	Yes	Yes	Yes	Yes	Cases occu	Yes	lack of staf
No	No	Yes	Yes	Yes	No	Cases occu	Yes	the langua
No	No	Yes	Yes	Yes	No	Cases occu	Yes	It is quite c
No	No	Yes	Yes	Yes	Yes	Cases occu	Yes	No
No GPS	No	Yes	Yes	Yes	Yes	Cases occu	Yes	No
Yes	Yes	Yes	Yes	Yes	Yes	Cases occu	Yes	Need to IE
No	No	Yes	Yes	Yes	Yes	Cases occu	Yes	The low in
Yes	Yes	Yes	Yes	Yes	Yes	Cases occu	Yes	The patien
No	No	Yes	Yes	Yes	Yes	Cases occu	Yes	No
No	No	Yes	Yes	Yes	Yes	Cases occu	Yes	The budge
Locating th	Yes	Yes	Yes	No	Yes	Cases origi	Yes	foreign cas
No	No	Yes	Yes	Yes	Yes	Cases occu	Yes	Population
Yes	Yes	Yes	Yes	Yes	Yes	Cases occu	Yes	Patient no
No	No	Yes	Yes	Yes	Yes	Cases occu	Yes	No funding
No	No	Yes	No	Yes	Yes	Cases origi	Yes	No
Yes	Yes	Yes	Yes	Yes	Yes	Both 1 and	Yes	No
Yes	Yes	Yes	Yes	Yes	Yes	Both 1 and	Yes	No
Yes	Yes	Yes	Yes	Yes	Yes	Both 1 and	Yes	do not see
determine	Yes	Yes	Yes	Yes	Yes	Both 1 and	Yes	Difficulties
No	No	Yes	Yes	Yes	Yes	Cases occu	Yes	lack of cad
Yes	Yes	Yes	Yes	Yes	Yes	Both 1 and	Yes	prepare a
Yes	Yes	Yes	Yes	No	No	Both 1 and	Yes	Can't find
Yes	Yes	Yes	Yes	No	No	Others	Yes	There is nc
No	No	Yes	Yes	Yes	Yes	Cases occu	Yes	Roads in p
No	No	Yes	Yes	Yes	No	Cases origi	Yes	Difficulties
have a sup	Yes	Yes	Yes	Yes	Yes	Others	Yes	Malaria pa
No	No	Yes	Yes	Yes	No	Both 1 and	Yes	No challen
prehistoric	Yes	Yes	Yes	Yes	No	Cases occu	Yes	compatrio
no, only in	No	Yes	Yes	Yes	No	Cases occu	Yes	movement
No	No	Yes	Yes	Yes	Yes	Cases occu	Yes	No challen
No	No	Yes	Yes	Yes	Yes	Cases origi	Yes	No challen
No	No	Yes	Yes	Yes	Yes	Cases occu	Yes	Language,
don't know	Don't know	Yes					Yes	Do not do
No	No	Yes	Yes	Yes	Yes	Others	No	no
No	No	Yes	Yes	Yes	Yes	Others	Yes	malaria pa
No	No	Yes	Yes	Yes	Yes	Cases occu	Yes	Funds, mai
Yes, they k	Yes	Yes	Yes	Yes	Yes	Cases occu	Yes	The low pc
Yes	Yes	Yes	Yes	Yes	Yes	Cases occu	Yes	The migrar
.		Yes	No				Yes	Few case r
Yes, we kn	Yes	Yes	Yes	Yes	No	Cases occu	Yes	They go to
Yes	Yes	Yes	Yes	Yes	Yes	Cases occu	Yes	Imported c
No	No	Yes	Yes	Yes	No	Others	Yes	difficult ab
No	No	Yes	Yes	Yes	No	Cases occu	Yes	to cotact tl
Yes, we go	Yes	Yes	Yes	Yes	No	Cases occu	Yes	The hard r
Yes	Yes	Yes	Yes	Yes	Yes	Cases occu	Yes	The people
Yes, go the	Yes	Yes	Yes	Yes	No	Cases occu	Yes	The difficu
No	No	Yes	Yes	Yes	Yes	Cases occu	Yes	the weathe
Yes, we us	Yes	Yes	Yes	Yes	Yes	Cases occu	Yes	They don't
No	No	Yes	Yes	No	No	Cases occu	No	No

No	No	Yes	Yes	Yes	Yes	Cases occu	Yes	The malari
Yes	Yes	Yes	Yes	No	Yes	Cases occu	Yes	Yes
yes, confir	Yes	Yes	Yes	Yes	No	Cases occu	Yes	No
No	No	Yes	Yes	Yes	No	Cases occu	Yes	Don't kno
No	No	Yes	Yes	Yes	Yes	Cases occu	Yes	no
No	No	Yes	Yes	Yes	No	Cases occu	Yes	No
No	No	Yes	Yes	Yes	Yes	Cases occu	Yes	Patient is r
No	No	Yes	Yes	Yes	Yes	Cases occu	Yes	are not
No	No	Yes	Yes	Yes	Yes	Cases occu	Yes	No
No	No	Yes	Yes	Yes	Yes	Cases occu	No	People far
No	No	Yes	Yes	Yes	No	Others	Yes	Lack of ma
No	No	Yes	Yes	Yes	Yes	Cases occu	Yes	Walking is
No	No		Yes	Yes	Yes	Cases occu	Yes	Going hom
No	No	Yes	Yes	Yes	No	Cases occu	Yes	There is no
No	No	Yes	Yes	Yes	Yes	Cases occu	Yes	Difficult to
No	No	Yes	Yes	Yes	Yes	Both 1 and	Yes	Ethnic min
No	No	Yes	Yes	Yes	No	Cases occu	Yes	No
No	No	Yes	Yes	Yes	Yes	Cases origi	Yes	People do
No	No	Yes	Yes	Yes	Yes	Cases occu	Yes	BN cannot
No	No	Yes	Yes	Yes	Yes	Both 1 and	Yes	No
No	No	Yes	Yes	Yes	Yes	Cases occu	Yes	Communic
The UCSF	No	Yes	Yes	Yes	Yes	Others	Yes	Difficulty w
No	No	Yes	Yes	Yes	Yes	Cases occu	Yes	Residents
.		Yes	Yes	Yes	No	Others	No	Unclear
No	No	Yes	Yes	Yes	No	Others	Yes	Lack of fun

CI challeng	CI challeng	CI challeng	CI challeng	CI challeng	CI challeng	CI challeng	Type of ca:	How soon
No	No	No	No	No	Yes	No	Every indig	Within 7 d
No	Yes	No	No	No	No	No	Every indig	Within 7 d
No	No	No	Yes	No	No	No	Every indig	Within 7 d
Yes	No	No	No	No	No	No	Every indig	Within 72
Yes	No	No	No	No	No	No	Every indig	Within 7 d
No	No	Yes	No	No	No	No	Every indig	Within 48
No	No	No	No	No	No	Yes	both 1 & 2	Within 72
No	No	No	Yes	No	No	No	Every indig	Within 7 d
Yes	No	No	No	No	No	No	Every indig	Within 48
No	No	No	No	Yes	No	No	Every indig	Within 28
No	No	No	Yes	No	No	No	both 1 & 2	Within 14
No	No	No	Yes	No	No	No	Every indig	Within 7 d
No	No	No	Yes	No	No	No	Every indig	Within 24
No	No	No	No	Yes	No	No	Every indig	Within 24
Yes	No	No	No	No	No	No	Every indig	Within 48
Yes	No	No	No	No	No	No	Every indig	Within 7 d
Yes	No	No	No	No	No	No	Every indig	Within 72
No	No	No	Yes	No	No	No	Every indig	Within 72
No	No	No	Yes	No	No	No	both 1 & 2	Within 24
No	No	No	Yes	No	Yes	No	both 1 & 2	Within 7 d
No	No	No	Yes	No	No	No	Every indig	Within 14
No	No	Yes	Yes	No	No	No	Every indig	Within 7 d
No	No	No	No	No	Yes	No	Every indig	Within 72
No	No	No	No	No	No	Yes	Every indig	Within 7 d
No	No	No	Yes	No	No	No	Every indig	Within 14
No	No	No	Yes	No	No	No	Every indig	Within 7 d
Yes	No	No	No	No	No	No	Every indig	Within 48
No	Yes	Yes	No	No	No	No	Every indig	Within 7 d
No	No	No	Yes	No	No	No	Every indig	Within 7 d
Yes	No	No	No	No	No	No	Every indig	Within 72
Yes	No	No	No	No	No	No	Every indig	Within 72
No	Yes	No	No	No	No	No	Every indig	Within 72
No	No	No	No	No	No	No		
Yes	No	No	No	No	No	No	Every indig	Within 7 d
No	No	No	Yes	No	No	No	Every indig	Within 72
No	No	No	No	Yes	Yes	No	Every indig	Within 72
							both 1 & 2	
No	No	No	Yes	No	No	No	Every indig	
No	No	Yes	Yes	No	No	No	Every indig	
No	No	Yes	Yes	No	No	No	both 1 & 2	
No	No	Yes	Yes	No	No	No	All importe	
No	No	No	No	No	No	Yes	All importe	
No	No	No	Yes	No	No	No	Every indig	
No	No	No	No	No	No	Yes	Every indig	
No	No	Yes	Yes	No	No	No	Every indig	
No	No	No	No	No	No	Yes	Every indig	
No	No	No	No	No	No	Yes	Every indig	
No	No	Yes	No	No	No	No	Every indig	
Yes	No	No	No	No	No	No	Every indig	

No	No	Yes	No	No	No	Yes	Every indig
							Every indig
Yes	No	No	No	No	No	No	All importe
							All importe
Yes	No	No	No	No	No	No	Every indig
Yes	No	No	No	No	No	No	Every indig
No	No	Yes	Yes	No	No	No	Every indig
Yes	No	No	No	No	No	No	Every indig
Yes	No	No	No	No	No	No	Every indig
No	No	No	Yes	No	No	No	Every indig
No	No	No	No	Yes	Yes	No	Every indig
No	No	No	No	No	No	Yes	Every indig
No	No	No	Yes	No	No	No	Every indig
Yes	No	No	No	No	No	No	Every indig
No	No	No	Yes	No	No	No	Every indig
No	No	No	Yes	No	No	No	Every indig
Yes	No	No	No	No	No	No	Every indig
No	No	Yes	No	No	No	No	Every indig
No	No	Yes	Yes	No	No	No	Every indig
Yes	No	No	No	No	No	No	Every indig
No	Yes	No	Yes	No	No	No	Every indig
No	No	Yes	No	No	No	Yes	Every indig
No	No	No	Yes	No	No	Yes	All importe
							Every indig
No	No	No	No	Yes	No	No	Every indig

How soon	Threshold	Screen hou	Types of h	What is do	If househo	If househo	If househo	If househo
Within 7 d	1	Always	All househ	Schedule a	No	Yes	No	No
Within 14	1	Never	All househ	Visit later t	Yes	No	No	No
Within 14	1	Always	Febrile cas	Schedule a	No	Yes	No	No
Within 7 d	1	Always	All househ	Schedule a	No	Yes	No	No
Within 7 d	1	Never	Febrile cas	Schedule a	No	Yes	No	No
Within 72	1	Always	All househ	Schedule a	No	Yes	No	No
Within 7 d	1	Always	All househ	Schedule a	No	Yes	No	No
Within 14	1	Always	All househ	Schedule a	No	Yes	No	No
Within 7 d	1	Always	All househ	Schedule a	No	Yes	No	No
Within 28	2	Always	All househ	Do not ret	No	No	Yes	No
Within 14	1	Always	All househ	Visit later t	Yes	No	No	No
Within 7 d	1	Always	All househ	Schedule a	No	Yes	No	No
Within 48	1	Always	All househ	Visit later t	Yes	No	No	No
Within 24	1	Always	All househ	Schedule a	No	Yes	No	No
Within 7 d	1	Always	All househ	Visit later t	Yes	No	No	No
Within 7 d	1	Always	All househ	Schedule a	No	Yes	No	No
Within 7 d	1	Always	All househ	Visit later t	Yes	No	No	No
Within 7 d	1	Always	All househ	Schedule a	No	Yes	No	No
Within 24	2	Always	Febrile cas	Schedule a	No	Yes	No	No
Within 7 d	1	Always	All househ	Other	No	No	No	Yes
Within 7 d	5	Always	All househ	Schedule a	No	Yes	No	No
Within 7 d	1	Always	All househ	Schedule a	No	Yes	No	No
Within 72	1	Always	All househ	Schedule a	No	Yes	No	No
Within 7 d	1	Sometimes	All househ	Schedule a	No	Yes	No	No
Within 28	3	Always	All househ	Schedule a	No	Yes	No	No
Within 7 d	1	Always	All househ	Schedule a	No	Yes	No	No
Within 28	1	Always	All househ	Schedule a	No	Yes	No	No
Within 7 d	1	Always	All househ	Visit later t	Yes	No	No	No
Within 7 d	1	Always	All househ	Schedule a	No	Yes	No	No
Within 7 d	1	Always	All househ	Visit later t	Yes	No	No	No
Within 72	1	Always	All househ	Visit later t	Yes	No	No	No
Within 7 d	1	Always	All househ	Schedule a	No	Yes	No	No
	1							
Within 7 d	1	Always	All househ	Schedule a	No	Yes	No	No
Within 7 d	1	Always	Febrile cas	Schedule a	No	Yes	No	No
Within 7 d	1	Always	All househ	Schedule a	No	Yes	No	No
	0	Always	Febrile cas	1.2	Yes	Yes	No	No
	0	Always	All househ	Schedule a	No	Yes	No	No
		Always	Febrile cas	Visit later t	Yes	No	No	No
	0	Always	All househ	Visit later t	Yes	No	No	No
	2	Always	All househ	Visit later t	Yes	No	No	No
	0	Always	All househ	Visit later t	Yes	No	No	No
	0	Always	All househ	1.2	Yes	Yes	No	No
	0	Always	Febrile cas	Visit later t	Yes	No	No	No
	0	Always	All househ	Visit later t	Yes	No	No	No
	0	Always	All househ	Schedule a	No	Yes	No	No
	0	Always	Febrile cas	Visit later t	Yes	No	No	No
	1	Never	Febrile cas	Visit later t	Yes	No	No	No
	1	Sometimes	Febrile cas	Schedule a	No	Yes	No	No

1	Always	All househ	Visit later t	Yes	No	No	No
1	Always	Febrile cas	Visit later t	Yes	No	No	No
1	Always	Febrile cas	Visit later t	Yes	No	No	No
1	Always	All househ	Schedule a	No	Yes	No	No
1	Always	All househ	Visit later t	Yes	No	No	No
1	Always	Febrile cas	Schedule a	No	Yes	No	No
1	Always	All househ	Visit later t	Yes	No	No	No
1	Sometimes	Febrile cas	Visit later t	Yes	No	No	No
1	Always	All househ	1.2	Yes	Yes	No	No
1	Always	All househ	Schedule a	No	Yes	No	No
1	Always	Febrile cas	Schedule a	No	Yes	No	No
	Always	All househ	Schedule a	No	Yes	No	No
	Always	All househ	Visit later t	Yes	No	No	No
1	Always	All househ	1.2	Yes	Yes	No	No
1	Always	All househ	Schedule a	No	Yes	No	No
	Always	All househ	Schedule a	No	Yes	No	No
	Always	All househ	Schedule a	No	Yes	No	No
1	Always	All househ	1.2	Yes	Yes	No	No
1	Always	All househ	Do not retu	No	No	Yes	No
	Always	All househ	Schedule a	No	Yes	No	No
1	Always	All househ	1.2	Yes	Yes	No	No
2	Always	All househ	Do not retu	No	No	Yes	No
1	Always	Febrile cas	Schedule a	No	Yes	No	No
1	Always	All househ	Visit later t	Yes	No	No	No
1	Always	All househ	1.3	Yes	No	Yes	No

Frequency	Type of ne	Trigger for	Screen a m	Screen a m	Screen a m	Time of th	Time for sc	Time for sc
Always	All neighbo	Local cases	Yes	Yes	Yes	2	No	Yes
Always	All neighbo	Local cases	Yes	No	Yes	2	No	Yes
Always	Febrile nei	Local cases	Yes	Yes	Yes	2	No	Yes
Always	All neighbo	Local cases	Yes	Yes	Yes	2	No	Yes
Sometimes	All neighbo	When loca	Yes	Yes	Yes	2	No	Yes
Always	Febrile nei	Local cases	Yes	Yes	Yes	3	No	No
Always	All neighbo	Local cases	Yes	No	Yes	2	No	Yes
Always	All neighbo	Local cases	Yes	Yes	Yes	2	No	Yes
Always	Febrile nei	Local cases	Yes	Yes	Yes	5	No	No
Always	All neighbo	Local cases	Yes	No	Yes	4.5	No	No
Always	All neighbo	Local and i	Yes	Yes	Yes	1.6	Yes	No
Sometimes	All neighbo	Local cases	Yes	Yes	Yes	1	Yes	No
Always	All neighbo	Local and i	Yes	Yes	Yes	1	Yes	No
Always	All neighbo	Local and i	Yes	No	Yes	1	Yes	No
Always	All neighbo	Imported c	Yes	Yes	No	6	No	No
Always	All neighbo	Local cases	Yes	No	Yes	1	Yes	No
Always	All neighbo	Local cases	Yes	No	Yes	1	Yes	No
Always	All neighbo	Local cases	Yes	Yes	No	1	Yes	No
Always	Febrile nei	Local and i	Yes	Yes	Yes	1.2	Yes	Yes
Always	All neighbo	Local and i	Yes	No	Yes	2	No	Yes
Always	All neighbo	Local cases	Yes	Yes	Yes	2	No	Yes
Always	All neighbo	Local cases	Yes	Yes	Yes	1	Yes	No
Always	Febrile nei	Local and i	Yes	Yes	Yes	2	No	Yes
Sometimes	All neighbo	Local cases	Yes	Yes	Yes	8	No	No
Always	All neighbo	Local and i	Yes	Yes	Yes	1	Yes	No
Always	Febrile nei	Local cases	Yes	No	Yes	5	No	No
Always	All neighbo	Local cases	Yes	Yes		1	Yes	No
Always	All neighbo	Local cases	Yes	No	No	1	Yes	No
Always	Febrile nei	Local cases	Yes	Yes	No	8	No	No
Always	All neighbo	Local cases	Yes	No	Yes	5	No	No
Always	All neighbo	Local cases	Yes	No	Yes	3	No	No
Always	All neighbo	Local cases	Yes	Yes	Yes	1.5	Yes	No
						.		
Always	Febrile nei	Local cases	Yes	Yes	Yes	4	No	No
Always	Febrile nei	Local cases	Yes	No	Yes	3	No	No
Always	All neighbo	Local cases	Yes	Yes	Yes	1	Yes	No
Always	All neighbo	Local and i	Yes	Yes	Yes	1,2,6,7,8	Yes	Yes
Always	Febrile nei	Local and i	Yes	No	Yes	6	No	No
Always	All neighbo	Local cases	Yes	No	Yes	4	No	No
Always	All neighbo	Local and i	Yes	No	Yes	1,5,6	Yes	No
Always	All neighbo	Local and i	No	No	No	2	No	Yes
Always	All neighbo	Local and i	Yes	No	Yes	1	Yes	No
Always	All neighbo	Local and i	Yes	No	Yes	1,2,5	Yes	Yes
Always	Febrile nei	Local and i	Yes	Yes	Yes	1,2,5	Yes	Yes
Always	All neighbo	Local cases	Yes	Yes	Yes	2.5.6.7	No	Yes
Always	Febrile nei	Local and i	Yes	Yes	Yes	1.2	Yes	Yes
Always	All neighbo	Local and i	Yes	No	No	1.2	Yes	Yes
Always	Febrile nei	Local and i	Yes	Yes	Yes	2.5	No	Yes
Always	Febrile nei	Local cases	Yes	No	Yes	1.8	Yes	No

Always	All neighbo	Local cases	Yes	Yes	Yes	1.2	Yes	Yes
Sometimes	Febrile nei	Local cases	Yes	Yes		1	Yes	No
Always	Febrile nei	Local and i	Yes	Yes	No	1	Yes	No
Always	All neighbo	Local and i	Yes	Yes	Yes	1	Yes	No
Always	All neighbo	Local cases	Yes	No	No	.		
Always	Febrile nei	Local and i	Yes	No	Yes	6	No	No
Always	All neighbo	Local cases	Yes	Yes	Yes	1,2,5	Yes	Yes
Sometimes	Febrile nei	Local cases	Yes	Yes	Yes	3	No	No
Always	All neighbo	Local cases	Yes	Yes	Yes	3	No	No
Always	All neighbo	Local cases	Yes	No	Yes	3	No	No
Always	Febrile nei	Local cases	Yes	No	Yes	6	No	No
Always	All neighbo	Local cases	Yes	No	Yes	6	No	No
Always	All neighbo	Local cases	Yes	No	Yes	6	No	No
Always	All neighbo	Local cases	Yes	Yes	Yes	4.6	No	No
Always	Febrile nei	Local cases	Yes	Yes	Yes	1.5	Yes	No
Always	All neighbo	Local cases	Yes	No	Yes	5	No	No
Always	Febrile nei	Local cases	Yes	No	Yes	6	No	No
Always	All neighbo	Local cases	Yes	Yes	Yes	3	No	No
Always	All neighbo	Local cases	Yes	Yes	Yes	2.5	No	Yes
Always	All neighbo	Local cases	Yes	No	Yes	2.5	No	Yes
Always	Febrile nei	Local and i	Yes	Yes	Yes	2.5	No	Yes
Always	All neighbo	Local cases	Yes	No	Yes	2	No	Yes
Sometimes	Febrile nei	Local and i	Yes	Yes	Yes	1	Yes	No
Always	All neighbo	Local cases	Yes	Yes	Yes	1	Yes	No
Always	All neighbo	Local cases	Yes	Yes	Yes	2	No	Yes

Time for sc	Time for sc	Time for sc	Time for sc	Time for sc	Time for sc	Return to s	Diagnosis r	Diagnosis f
No	No	No	No	No	No	Yes	1.2	Yes
No	No	No	No	No	No	Yes	1.2	Yes
No	No	No	No	No	No	Yes	1.2	Yes
No	No	No	No	No	No	Yes	1.2	Yes
No	No	No	No	No	No	Yes	1.2	Yes
Yes	No	No	No	No	No	Yes	2	No
No	No	No	No	No	No	Yes	1.2	Yes
No	No	No	No	No	No	Yes	1	Yes
No	No	Yes	No	No	No	Yes	1.2	Yes
No	Yes	Yes	No	No	No	No	1.2	Yes
No	No	No	Yes	No	No	Yes	1.2	Yes
No	No	No	No	No	No	Yes	1.2	Yes
No	No	No	No	No	No	Yes	1	Yes
No	No	No	No	No	No	Yes	1	Yes
No	No	No	Yes	No	No	Yes	2	No
No	No	No	No	No	No	Yes	1.2	Yes
No	No	No	No	No	No	Yes	1.2	Yes
No	No	No	No	No	No	Yes	1,2,4	Yes
No	No	No	No	No	No	Yes	1,2,3,4,5	Yes
No	No	No	No	No	No	No	1.2	Yes
No	No	No	No	No	No	No	1.2	Yes
No	No	No	No	No	No	Yes	1,2,3	Yes
No	No	No	No	No	No		1	Yes
No	No	No	No	No	Yes	No	1.2	Yes
No	No	No	No	No	No	Yes	1	Yes
No	No	Yes	No	No	No	Yes	2	No
No	No	No	No	No	No	Yes	1	Yes
No	No	No	No	No	No	Yes	1.2	Yes
No	No	No	No	No	Yes	Yes	1,2,4	Yes
No	No	Yes	No	No	No	Yes	1,2,4	Yes
Yes	No	No	No	No	No	No	1.2	Yes
No	No	Yes	No	No	No	Yes	1.2	Yes
							.	
No	Yes	No	No	No	No	Yes	1.2	Yes
Yes	No	No	No	No	No	No	1	Yes
No	No	No	No	No	No	Yes	1.2	Yes
No	No	No	Yes	Yes	Yes	Yes	1,2,4	Yes
No	No	No	Yes	No	No	Yes	1.2	Yes
No	Yes	No	No	No	No	Yes	2	No
No	No	Yes	Yes	No	No	Yes	1	Yes
No	No	No	No	No	No	Yes	2	No
No	No	No	No	No	No	Yes	2	No
No	No	Yes	No	No	No	Yes	1.2	Yes
No	No	Yes	No	No	No	Yes	1.2.4	Yes
No	No	Yes	Yes	Yes	No	Yes	1.2.4	Yes
No	No	No	No	No	No	Yes	2	No
No	No	No	No	No	No	Yes	2	No
No	No	Yes	No	No	No	Yes	2	No
No	No	No	No	No	Yes	No	1	Yes

No	No	No	No	No	No	Yes	1,2,4	Yes
No	No	No	No	No	No	No	2	No
No	No	No	No	No	No	No	2	No
No	No	No	No	No	No	No	2	No
						Yes	1.2	Yes
No	No	No	Yes	No	No	Yes	2	No
No	No	Yes	No	No	No	Yes	1.2	Yes
Yes	No	No	No	No	No	Yes	1	Yes
Yes	No	No	No	No	No	Yes	1.2	Yes
Yes	No	No	No	No	No	Yes	2	No
No	No	No	Yes	No	No	Yes	1	Yes
No	No	No	Yes	No	No	Yes	1.2	Yes
No	No	No	Yes	No	No	Yes	2	No
No	Yes	No	Yes	No	No		1.2	Yes
No	No	Yes	No	No	No	Yes	1.2	Yes
No	No	Yes	No	No	No	Yes	1.2	Yes
No	No	No	Yes	No	No	Yes	1.2	Yes
Yes	No	No	No	No	No	Yes	1	Yes
No	No	Yes	No	No	No	No	1.2	Yes
No	No	Yes	No	No	No	Yes	1.2	Yes
No	No	Yes	No	No	No	Yes	1.2	Yes
No	No	No	No	No	No	No	1.2	Yes
No	No	No	No	No	No	Yes	2	No
No	No	No	No	No	No	Yes	.	
No	No	No	No	No	No	Yes	1.2	Yes

Diagnosis f	Diagnosis f	Diagnosis f	Diagnosis f	Number of	Challenges	Screening	Screening	Screening
Yes	No	No	No	Single conf	No challen	Yes	No	No
Yes	No	No	No	Single conf	The langua	No	Yes	Yes
Yes	No	No	No	Single conf	it is difficul	No	No	No
Yes	No	No	No	Single conf	No challen	Yes	No	No
Yes	No	No	No	Other thre	No challen	Yes	No	No
Yes	No	No	No	Other thre	No challen	Yes	No	No
Yes	No	No	No	Single conf	To mobiliz	No	No	No
No	No	No	No	Single conf	No challen	Yes	No	No
Yes	No	No	No	Single conf	The citizen	No	No	No
Yes	No	No	No	Other thre	No patient	No	No	No
Yes	No	No	No	Single conf	Investigati			
Yes	No	No	No	Single conf	Don't know			
No	No	No	No	Single conf	No funds t	No	No	No
No	No	No	No	>1 confirm	lack of fun	No	No	No
Yes	No	No	No	Single conf	No	Yes	No	No
Yes	No	No	No	Single conf	no	Yes	No	No
Yes	No	No	No	Single conf	Patient no	No	No	No
Yes	No	Yes	No	Single conf	Not enoug	No	No	No
Yes	Yes	Yes	Yes	Other thre	Not enoug	No	No	No
Yes	No	No	No	Single conf	Group orga	No	No	No
Yes	No	No	No	Single conf	Traffic is di	No	No	No
Yes	Yes	No	No	Single conf	uncoopera	No	No	Yes
No	No	No	No	Single conf	No human	No	No	No
Yes	No	No	No	Single conf	difficult to	No	No	No
No	No	No	No	Single conf	danger of s			
Yes	No	No	No	Single conf	There is TH			
No	No	No	No	Single conf	There is nc	No	No	No
Yes	No	No	No	Single conf	difficult ro	No	No	No
Yes	No	Yes	No	Single conf	Human res	No	No	No
Yes	No	Yes	No	Single conf	The same i	No	No	No
Yes	No	No	No	Single conf	no challeng	Yes	No	No
Yes	No	No	No	Single conf	Funds, mea	No	No	No
					.			
Yes	No	No	No	Single conf	Family mei	No	No	No
No	No	No	No	>1 confirm	People do	No	No	Yes
Yes	No	No	No	Single conf	Funds, mea	No	No	No
Yes	No	Yes	No	Single conf	high poupa	No	No	No
Yes	No	No	No	Single conf	.			
Yes	No	No	No	Single conf	There is m	No	No	No
No	No	No	No		detect the	No	No	No
Yes	No	No	No	Single conf	No chanler	Yes	No	No
Yes	No	No	No	Single conf	From the c			
Yes	No	No	No	Single conf	To control	No	No	No
Yes	No	Yes	No	Single conf	The people	No	No	No
Yes	No	Yes	No	Single conf	The people	No	No	No
Yes	No	No	No	Single conf	The patien	No	No	No
Yes	No	No	No	Single conf	They don't	No	No	No
Yes	No	No	No	Don't know	The people	No	No	No
No	No	No	No	>1 confirm	Yes			

Yes	No	Yes	No	Single conf	The terribl	No	No	No
Yes	No	No	No	Single conf	Yes			
Yes	No	No	No	Other thre	It is difficul	No	No	No
Yes	No	No	No	>1 confirm	Yes			
Yes	No	No	No	Single conf	budget	No	No	No
Yes	No	No	No	Single conf	Many peop	No	No	Yes
Yes	No	No	No	Single conf	Can't see p	No	No	No
No	No	No	No	Single conf	.			
Yes	No	No	No	Single conf	People are	No	No	Yes
Yes	No	No	No	Single conf	Not at hon	No	No	No
No	No	No	No	Single conf	The family	No	No	No
Yes	No	No	No	Single conf	Not availal	No	No	No
Yes	No	No	No	Single conf	.			
Yes	No	No	No	Single conf	Difficulty v	No	No	Yes
Yes	No	No	No	Single conf	No	Yes	No	No
Yes	No	No	No	Single conf	Difficulty t	No	No	No
Yes	No	No	No	Single conf	No	Yes	No	No
No	No	No	No	Single conf	People do	No	No	Yes
Yes	No	No	No	Single conf	People do	No	No	Yes
Yes	No	No	No	Single conf	Mostly, pa	No	No	Yes
Yes	No	No	No	Single conf	Difficult wa	No	No	Yes
Yes	No	No	No	Single conf	There mus	No	No	No
Yes	No	No	No	Single conf	People's kn	No	No	Yes
				Don't know	.			
Yes	No	No	No	Single conf	Some peop	No	No	Yes

Screening	Screening	Screening	Screening	Type of res	Response a	Response a	Response a	Response a
No	No	No	No	1,2,4	Yes	Yes	No	Yes
No	No	No	No	1,2,3,4	Yes	Yes	Yes	Yes
Yes	No	No	No	1.2	Yes	Yes	No	No
No	No	No	No	1,2,4	Yes	Yes	No	Yes
No	No	No	No	1.3	Yes	No	Yes	No
No	No	No	No	1	Yes	No	No	No
No	Yes	No	No	1,2,3	Yes	Yes	Yes	No
No	No	No	No	2	No	Yes	No	No
Yes	Yes	No	No	.				
Yes	No	No	No	1.2	Yes	Yes	No	No
				1,2,5	Yes	Yes	No	No
				1,2,3,4,5	Yes	Yes	Yes	Yes
No	Yes	No	No	1,2,3	Yes	Yes	Yes	No
No	Yes	No	No	1,2,3,4	Yes	Yes	Yes	Yes
No	No	No	No	1	Yes	No	No	No
No	No	No	No	1,2,3,4	Yes	Yes	Yes	Yes
Yes	No	No	No	1,2,3,4	Yes	Yes	Yes	Yes
No	No	Yes	No	1,2,3,4	Yes	Yes	Yes	Yes
No	No	Yes	No	1,2,3,4,5	Yes	Yes	Yes	Yes
No	Yes	Yes	No	2.3	No	Yes	Yes	No
No	No	No	Yes	1,2,3,4	Yes	Yes	Yes	Yes
No	No	No	No	2.3	No	Yes	Yes	No
No	Yes	Yes	No	3.5	No	No	Yes	No
Yes	No	No	No	1,2,3	Yes	Yes	Yes	No
				2	No	Yes	No	No
				1,2,3,4	Yes	Yes	Yes	Yes
No	Yes	No	No	1,2,3,4	Yes	Yes	Yes	Yes
No	No	No	Yes	1,2,3,4,5	Yes	Yes	Yes	Yes
No	No	Yes	Yes	2,3,4	No	Yes	Yes	Yes
Yes	No	No	No	1,2,3,5	Yes	Yes	Yes	No
No	No	No	No	1.2	Yes	Yes	No	No
No	Yes	Yes	No	1,2,3	Yes	Yes	Yes	No
				.				
Yes	No	No	No	2	No	Yes	No	No
No	No	Yes	No	1,2,3,4	Yes	Yes	Yes	Yes
No	Yes	Yes	No	1,2,3	Yes	Yes	Yes	No
Yes	No	No	No	1.2	Yes	Yes	No	No
				3	No	No	Yes	No
Yes	No	No	No	1	Yes	No	No	No
Yes	No	No	No	1.2	Yes	Yes	No	No
No	No	No	No	1	Yes	No	No	No
				1.2	Yes	Yes	No	No
Yes	No	No	No	1.2	Yes	Yes	No	No
Yes	No	No	No	1.2	Yes	Yes	No	No
Yes	No	No	No	2	No	Yes	No	No
Yes	No	No	No	1.2	Yes	Yes	No	No
Yes	No	No	No	1.5	Yes	No	No	No
Yes	No	No	No	1.2	Yes	Yes	No	No
				5	No	No	No	No

No	No	No	Yes	1,2,3	Yes	Yes	Yes	No
				4	No	No	No	Yes
Yes	No	No	No	2	No	Yes	No	No
				1,2,3	Yes	Yes	Yes	No
No	Yes	No	No	1,2,4	Yes	Yes	No	Yes
No	No	No	No	1,2,5	Yes	Yes	No	No
Yes	No	No	Yes	1,2,3	Yes	Yes	Yes	No
				1.2	Yes	Yes	No	No
No	No	No	No	1,2,3,4	Yes	Yes	Yes	Yes
Yes	No	No	No	2	No	Yes	No	No
Yes	No	No	No	2.3	No	Yes	Yes	No
Yes	No	No	No	1.2	Yes	Yes	No	No
				2	No	Yes	No	No
No	No	No	Yes	1.2	Yes	Yes	No	No
No	No	No	No	1,2,3	Yes	Yes	Yes	No
Yes	No	No	Yes	1.2	Yes	Yes	No	No
No	No	No	No	1,2,5	Yes	Yes	No	No
No	No	No	No	1.2	Yes	Yes	No	No
No	No	No	No	1,2,3	Yes	Yes	Yes	No
No	No	No	No	1.2	Yes	Yes	No	No
No	No	No	Yes	1,2,3	Yes	Yes	Yes	No
Yes	No	No	No	1,2,3,4	Yes	Yes	Yes	Yes
No	No	No	No	1.2	Yes	Yes	No	No
				1	Yes	No	No	No
No	No	No	No	2	No	Yes	No	No

Response a	How soon	How soon	How soon	Any specifi	Any specifi	Infomation	Infomation	Current RA
No	Within 7 d	Within 7 d	Within 7 d	Yes	Yes	Yes, we dic	Yes	Yes
No	Within 28	Within 28	Within 14	Distubute	Yes	Supervise t	Yes	Yes
No	Within 7 d	Within 14	Within 28	Yes, try to	Yes	Yes, to clas	Yes	Eliminate F
No	Within 7 d	Within 7 d	Within 7 d	Yes, we ha	Yes	Yes	Yes	Yes
No	Within 48	Within 7 d	Within 7 d	Yes	Yes	Yes, accura	Yes	yes
No	Within 24	Within 72	Within 72	No	No	Yes	Yes	Yes
No	Within 7 d	Within 7 d	Within 48	Distubute	Yes	Yes, it catc	Yes	Yes, To det
No	Within 48	Within 28	Within 28	No	No	No	No	Yes
	Within 48	Within 24	Within 7 d	Yes	Yes	To access t	Yes	To reduce
No	Within 28	Within 28	Within 72	it does not	No	Yes, to ma	Yes	Yes, monit
Yes	Within 7 d	Within 7 d	Within 7 d	Yes	Yes	Yes, impac	Yes	Yes
Yes	Within 14	Within 14	Within 7 d	Yes	Yes	Yes	Yes	Yes
No	Within 24	Within 24	Within 7 d	Yes, so as	Yes	Yes	Yes	Yes
No	Within 24	Within 24	Within 24	yes	Yes	yes	Yes	no
No	Within 7 d	Within 24	Within 24	No	No	Have a big	Yes	Has a grea
No	Within 24	Within 72	Within 24	Yes	Yes	Yes, timely	Yes	Yes, monit
No	Within 7 d	Within 72	Within 24	No	No	Yes, if the	Yes	Yes, if the
No	Within 72	Within 24	Within 28	yes	Yes	Positive im	Yes	yes
Yes	Within 24	Within 24	Within 72	yes	Yes	manage th	Yes	yes
No	Within 7 d	Within 7 d	Within 7 d	Yes	Yes	yes, as the	Yes	yes
No	Within 48	Within 24	Within 14	yes	Yes	yes, plan ir	Yes	incomplete
No	Within 24	Within 48	Within 7 d	yes	Yes	yes, don't	Yes	yes
Yes	Within 7 d	Within 7 d	Within 7 d	yes	Yes	yes, helps	Yes	yes
No	Within 72	Within 24	Within 24	yes	Yes	Yes, it help	Yes	yes
No	Within 24	Within 24	Within 14	yes	Yes	yes	Yes	yes
No	Within 7 d	Within 7 d	Within 28	Yes	Yes	Yes, deter	Yes	Yes, P. viva
No	Within 7 d	Within 14	Within 28	Yes, go to	Yes	Yes, epide	Yes	yes, comm
Yes	Within 72	Within 7 d	Within 28	yes, propa	Yes	yes, contri	Yes	yes, avoid
No	Within 7 d	Within 7 d	Within 28	There shou	No	have an im	Yes	yes
Yes	Within 7 d	Within 7 d	Within 24	Yes, village	Yes	have a maj	Yes	yes, becau
No	Within 7 d	Within 7 d	Within 72	yes	Yes	yes	Yes	yes
No	Within 48	Within 72	Within 7 d	yes	Yes	Yes, if ther	Yes	Yes, anti-ir
				.		.		.
No	Within 7 d	Within 7 d	Within 28	yes	Yes	Yes, better	Yes	No
No	After 28 da	Within 7 d	Within 28	Yes, propa	Yes	Have	Yes	yes
No	Within 48	Within 7 d	Within 14	yes but no	Yes	contribute	Yes	there is a r
No	Within 24	Within 7 d	Within 28	.		Yes, contro	Yes	.
No	Within 48	Within 7 d	Within 14	.		Stop trans	Yes	.
No	Within 72	Within 72	Within 72	.		Help the a	Yes	.
No	Within 24	Within 24	Within 24	.		To control	Yes	.
No	Within 28	Within 28	Within 28	.		No	No	.
No	Within 7 d	Within 7 d	Within 24	.		No	No	.
No	Within 72	Within 24	Within 72	.		No	No	.
No	Within 24	Within 48	Within 72	.		Yes, to mo	Yes	.
No	Within 24	Within 48	Within 72	.		Yes, to mo	Yes	.
No	Within 24	Within 48	Within 72	.		Have the k	Yes	.
Yes	Within 24	Within 48	Within 72	.		Yes, they a	Yes	.
No	Within 24	Within 48	Within 72	.		it is convin	Yes	.
Yes	Within 72	Within 14	Within 14	.		No	No	.

No	Within 48	Within 72	Within 7 d	.	Yes, reduc	Yes	.
No	Within 7 d	Within 48	Within 7 d	.	Yes, we cla	Yes	.
No	Within 24	Within 24	Within 24	.	No	No	.
No	Within 48	Within 48	Within 24	.	Yes, there	Yes	.
No	Within 7 d	Within 7 d	Within 24	.	no	No	.
Yes	Within 7 d	Within 72	Within 24	.	Yes, reduc	Yes	.
No	Within 24	Within 48	Within 72	.	Yes, chang	Yes	.
No	Within 72	Within 7 d	Within 7 d	.	Yes	Yes	.
No	Within 24	Within 72	Within 7 d	.	Yes	Yes	.
No	Within 72	Within 72	Within 72	.	No	No	.
No	Within 24	Within 48	Within 24	.	No	No	.
No	Within 24	Within 24	Within 24	.	No impact	No	.
No	Within 7 d	Within 48	Within 7 d	.	.		.
No	Within 48	Within 7 d	Within 7 d	.	No	No	.
No	Within 48	Within 72	Within 7 d	.	Effective ir	Yes	.
No	Within 24	Within 24	Within 24	.	.		.
Yes	Within 24	Within 24	Within 24	.	No	No	.
No	Within 24	Within 72	Within 14	.	Have	Yes	.
No	Within 48	Within 72	Within 7 d	.	Helping pe	Yes	.
No	Within 24	Within 24	Within 24	.	.		.
No	Within 48	Within 72	Within 7 d	.	Help raise	Yes	.
No	Within 7 d	Within 7 d	Within 14	.	Propagand	Yes	.
No	Within 24	Within 24	Within 48	.	Reducing t	Yes	.
No	Within 14	Within 28	Within 28	.	.		.
No	Within 24	Within 72	Within 7 d	.	Good	Yes	.

Current RA	Current ba	Noti barrri	Noti barrie	Noti barrie	Noti barrie	Noti barrie	Noti barrie	Noti barrie
Yes	Yes	No	No	No	No	No	No	No
Yes	Can do it	Yes	No	No	No	No	No	No
Yes	Yes, it is ha	No	No	No	No	Yes	No	No
Yes	No	Yes	No	No	No	No	No	No
Yes	It is hard tc	No	No	No	No	No	No	No
Yes	NO	Yes	No	No	No	No	No	No
Yes	No	Yes	No	No	No	No	No	No
Yes	Yes	No	No	No	No	No	No	No
Yes	Yes	No	No	No	No	No	No	No
Yes	No challen	Yes	No	No	No	No	No	No
Yes	No	Yes	No	No	No	No	No	No
Yes	Yes	No	No	No	No	No	No	No
Yes	Yes but dif	No	No	No	No	No	No	No
No	no	Yes	No	No	No	No	No	No
Yes	No	Yes	No	No	No	No	No	No
Yes	No	Yes	No	No	No	No	No	No
Yes	no, totally	Yes	No	No	No	No	No	No
Yes	Due to diff	No	No	No	Yes	No	No	Yes
Yes	yes	No	No	No	No	No	No	No
Yes	Do you ne	No	No	No	No	Yes	No	No
No	YES	No	No	No	No	No	No	No
Yes	no	Yes	No	No	No	No	No	No
Yes	yes	No	No	No	No	No	No	No
Yes	no	Yes	No	No	No	No	No	No
Yes	yes	No	No	No	No	No	No	No
Yes	There are	No	No	No	No	No	No	Yes
Yes	Doable	Yes	No	No	No	No	No	No
Yes	yes	No	No	No	No	No	No	No
Yes	are not	Yes	No	No	No	No	No	No
Yes	are not	Yes	No	No	No	No	No	No
Yes	are not	Yes	No	No	No	No	No	No
Yes	No	Yes	No	No	No	No	No	No
	.							
No	Yes	No	No	No	No	No	No	No
Yes	difficult	No	No	No	No	No	No	No
Yes	Yes, need	No	No	No	No	Yes	No	No
	Yes, not er	No	No	No	No	Yes	No	No
	Yes, relate	No	No	No	No	No	Yes	No
	It is difficul	No	No	No	No	No	No	No
	2 days	No	No	No	No	Yes	No	No
	No	Yes	No	No	No	No	No	No
	No	Yes	No	No	No	No	No	No
	No	Yes	No	No	No	No	No	No
	Yes; Becau	No	No	Yes	No	No	No	No
	Yes, it is nc	No	No	Yes	Yes	Yes	No	No
	Yes, it is nc	No	No	No	No	Yes	No	No
	Yes, there	No	No	No	Yes	No	No	No
	It is not en	No	No	No	No	Yes	No	No
	Yes	No	No	No	No	No	No	No

Yes	No	No	No	No	No	No	No
Yes	No	No	No	No	No	No	No
No	Yes	No	No	No	No	No	No
No	Yes	No	No	No	No	No	No
no	Yes	No	No	No	No	No	No
No	Yes	No	No	No	No	No	No
Good	Yes	No	No	No	No	No	No
No	Yes	No	No	No	No	No	No
No	Yes	No	No	No	No	No	No
No	Yes	No	No	No	No	No	No
No	Yes	No	No	No	No	No	No
No	Yes	No	No	No	No	No	No
No	Yes	No	No	No	No	No	No
Have	No	No	No	No	No	No	No
No	Yes	No	No	No	No	No	No
No	Yes	No	No	No	No	No	No
No	Yes	No	No	No	No	No	No
No	Yes	No	No	No	No	No	No
No	Yes	No	No	No	No	No	No
No	Yes	No	No	No	No	No	No
The inform	No	No	Yes	No	No	No	No
No networ	No	No	Yes	No	No	No	No
No	Yes	No	No	No	No	No	No
No	Yes	No	No	No	No	No	No
No becaus	Yes	No	No	No	No	No	No

Noti barrie	Current ba	CI barrrier	CI barrier -	CI barrier -	CI barrier -	CI barrier -	CI barrier -	CI barrier -
Yes	Yes	No	No	No	No	No	No	No
No	Can do it	Yes	No	No	No	No	No	No
No	NO	Yes	No	No	No	No	No	No
No	No	Yes	No	No	No	No	No	No
Yes	No, no cha	Yes	No	No	No	No	No	No
No	NO	Yes	No	No	No	No	No	No
No	No, no cha	Yes	No	No	No	No	No	No
Yes	No	Yes	No	No	No	No	No	No
Yes	Yes	No	No	No	No	No	No	No
No	Yes, time i	No	No	No	No	Yes	No	No
No	NO	Yes	No	No	No	No	No	No
Yes	Yes	No	No	No	No	No	No	No
Yes	Have	No	No	No	No	No	No	No
No	yes	No	No	No	No	No	No	No
No	There is a	No	No	No	No	No	No	No
No	No	Yes	No	No	No	No	No	No
No	no, totally	Yes	No	No	No	No	No	No
No	Yes	No	No	No	No	No	No	No
Yes	yes	No	No	No	No	No	No	No
No	no	Yes	No	No	No	No	No	No
Yes	YES	No	No	No	No	No	No	No
No	no	Yes	No	No	No	No	No	No
Yes	yes	No	No	No	No	No	No	No
No	no	Yes	No	No	No	No	No	No
Yes	yes	No	No	No	No	No	No	No
No	No	Yes	No	No	No	No	No	No
No	Do able	Yes	No	No	No	No	No	No
Yes	yes	No	No	No	No	No	No	No
No	No	Yes	No	No	No	No	No	No
No	No	Yes	No	No	No	No	No	No
No	No	Yes	No	No	No	No	No	No
No	Yes	No	No	No	No	No	No	No
	.							
Yes	No	Yes	No	No	No	No	No	No
Yes	Difficult bu	No	No	No	No	No	No	No
No	No	Yes	No	No	No	No	No	No
No	No, That ti	Yes	No	No	No	No	No	No
No	No	Yes	No	No	No	No	No	No
Yes	No	Yes	No	No	No	No	No	No
No	No	Yes	No	No	No	No	No	No
No	No	Yes	No	No	No	No	No	No
No	No	Yes	No	No	No	No	No	No
No	NO	Yes	No	No	No	No	No	No
No	No	Yes	No	No	No	No	No	No
No	No	Yes	No	No	No	No	No	No
No	No	Yes	No	No	No	No	No	No
No	Yes, it is nc	No	No	No	No	Yes	No	No
No	no	Yes	No	No	No	No	No	No
Yes	3 days is cl	No	No	No	No	Yes	No	No

Yes	No	Yes	No	No	No	No	No	No
Yes	Yes, it is 7	No	No	No	No	Yes	No	No
No	No	Yes	No	No	No	No	No	No
No	No	Yes	No	No	No	No	No	No
No	no	Yes	No	No	No	No	No	No
No	No	Yes	No	No	No	No	No	No
No	yes	No	No	No	No	No	No	No
No	No	Yes	No	No	No	No	No	No
No	No	Yes	No	No	No	No	No	No
No	No	Yes	No	No	No	No	No	No
No	yes	No	No	No	No	No	No	No
No	No	Yes	No	No	No	No	No	No
No	No	Yes	No	No	No	No	No	No
Yes	yes	No	No	No	No	No	No	No
No	No	Yes	No	No	No	No	No	No
No	Fear of not	No	No	No	Yes	Yes	No	No
No	No	Yes	No	No	No	No	No	No
No	No	Yes	No	No	No	No	No	No
No	No	Yes	No	No	No	No	No	No
No	No	Yes	No	No	No	No	No	No
No	Difficult wa	No	No	No	Yes	No	No	Yes
No	Difficulty w	No	No	No	No	No	No	Yes
No	No	Yes	No	No	No	No	No	No
No	No	Yes	No	No	No	No	No	No
No	No	Yes	No	No	No	No	No	No

CI barrier -	Current ba	FIR barrrie	FIR barrier	FIR barrier	FIR barrier	FIR barrier	FIR barrier	FIR barrier
Yes	Yes	No	No	No	No	No	No	No
No	Need to m	No	No	No	No	Yes	No	No
No	No	Yes	No	No	No	No	No	No
No	No	Yes	No	No	No	No	No	No
No	No, no cha	Yes	No	No	No	No	No	No
No	NO	Yes	No	No	No	No	No	No
No	Can do it	Yes	No	No	No	No	No	No
No	No	Yes	No	No	No	No	No	No
Yes	Yes	No	No	No	No	No	No	No
No	No	Yes	No	No	No	No	No	No
No	No	Yes	No	No	No	No	No	No
Yes	Yes	No	No	No	No	No	No	No
Yes	Normal	Yes	No	No	No	No	No	No
Yes	yes	No	No	No	No	No	No	No
Yes	There is a (	No	No	No	No	No	No	No
No	Yes, there	No	No	No	Yes	No	No	No
No	no, totally	Yes	No	No	No	No	No	No
Yes	No	Yes	No	No	No	No	No	No
Yes	yes	No	No	No	No	No	No	No
No	no	Yes	No	No	No	No	No	No
Yes	YES	No	No	No	No	No	No	No
No	no	Yes	No	No	No	No	No	No
Yes	yes	No	No	No	No	No	No	No
No	no	Yes	No	No	No	No	No	No
Yes	yes	No	No	No	No	No	No	No
No	NO	Yes	No	No	No	No	No	No
No	Difficulty ir	No	No	No	No	No	Yes	No
Yes	are not	Yes	No	No	No	No	No	No
No	are not	Yes	No	No	No	No	No	No
No	are not	Yes	No	No	No	No	No	No
No	are not	Yes	No	No	No	No	No	No
Yes	No	Yes	No	No	No	No	No	No
	.							
No	No	Yes	No	No	No	No	No	No
Yes	difficult	No	No	No	No	No	No	No
No	No	Yes	No	No	No	No	No	No
No	No	Yes	No	No	No	No	No	No
No	No	Yes	No	No	No	No	No	No
No	No challen	Yes	No	No	No	No	No	No
No	Yes, few da	No	No	No	No	Yes	No	No
No	No	Yes	No	No	No	No	No	No
No	No	Yes	No	No	No	No	No	No
No	No	Yes	No	No	No	No	No	No
No	no	Yes	No	No	No	No	No	No
No	No	Yes	No	No	No	No	No	No
No	No	Yes	No	No	No	No	No	No
No	No, it is no	No	No	No	No	Yes	No	No
No	no	Yes	No	No	No	No	No	No
No	7 days is cl	No	No	No	No	Yes	No	No

No	No	Yes	No	No	No	No	No	No
No	Yes	No	No	No	No	No	No	No
No	No	Yes	No	No	No	No	No	No
No	no	Yes	No	No	No	No	No	No
No	no	Yes	No	No	No	No	No	No
No	No	Yes	No	No	No	No	No	No
Yes	yes	No	No	No	No	No	No	No
No	No	Yes	No	No	No	No	No	No
No	No	Yes	No	No	No	No	No	No
No	No	Yes	No	No	No	No	No	No
Yes	No	Yes	No	No	No	No	No	No
No	No	Yes	No	No	No	No	No	No
No	No	Yes	No	No	No	No	No	No
Yes	No	Yes	No	No	No	No	No	No
No	No	Yes	No	No	No	No	No	No
No	No	Yes	No	No	No	No	No	No
No	No	Yes	No	No	No	No	No	No
No	No	Yes	No	No	No	No	No	No
No	Difficult to	No	No	No	Yes	No	No	No
No	No	Yes	No	No	No	No	No	No
No	Patient is c	No	No	No	Yes	No	No	No
No	During the							
No	No	Yes	No	No	No	No	No	No
No	No	Yes	No	No	No	No	No	No
No	No	Yes	No	No	No	No	No	No

FIR barrier	Current ba	RASR barrı	RASR barri	RASR barri	RASR barri	RASR barri	RASR barri	RASR barri
Yes	No	Yes	No	No	No	No	No	No
No	.							
No	Cutting do	No	No	No	No	No	No	Yes
No	No	Yes	No	No	No	No	No	No
No	finance an	No	No	No	No	No	No	Yes
No	finance an	No	No	No	No	No	No	Yes
No	No	Yes	No	No	No	No	No	No
No	The budge	No	No	No	No	No	No	Yes
Yes	Reduce ma	No	No	No	Yes	No	No	No
No	The budge	No	No	No	No	No	No	Yes
No	Influencing	No	No	No	No	No	No	No
Yes	Budget	No	No	No	No	No	No	Yes
No	yes	No	No	No	No	No	No	No
Yes	yes	No	No	No	No	No	No	No
Yes	Great Barr	No	No	No	No	No	No	No
No	Not availal							
No	Staff in cha	No	No	No	No	No	No	No
No	don't know							
Yes	yes	No	No	No	No	No	No	No
No	arrange sp	No	No	No	No	No	No	No
Yes	EXPENSE	No	No	No	No	No	No	Yes
No	Malaria pa	No	No	No	Yes	No	No	No
Yes	less manpo	No	No	No	No	No	No	Yes
No	Malaria is	No	No	No	Yes	No	No	No
Yes	alien, imm	No	No	No	No	Yes	No	No
No	There are l	No	No	No	No	No	No	No
No	Funds, rota	No	No	No	No	No	No	Yes
No	manpower	No	No	No	No	No	No	No
No	manpower	No	No	No	No	No	No	No
No	manpower	No	No	No	No	No	No	No
No	are not	Yes	No	No	No	No	No	No
No	Lack of hur	No	No	No	No	No	No	Yes
	.							
No	No	Yes	No	No	No	No	No	No
Yes	Time	No	No	No	No	No	Yes	No
No	Funds, mai	No	No	No	Yes	No	No	Yes
No	No	Yes	No	No	No	No	No	No
No	No	Yes	No	No	No	No	No	No
No	Need to IE	No	No	No	Yes	No	No	No
No	No	Yes	No	No	No	No	No	No
No	NO	Yes	No	No	No	No	No	No
No	Communit	No	No	No	Yes	No	No	No
No	IEC, strentl	No	No	No	Yes	No	No	No
No	The peopl	No	No	No	Yes	No	No	No
No	The aware	No	No	No	Yes	No	No	No
No	The aware	No	No	No	Yes	No	No	No
No	The aware	No	No	No	Yes	No	No	No
No	There are	No	No	No	Yes	No	No	No
No	No	Yes	No	No	No	No	No	No

No	No	Yes	No	No	No	No	No	No
Yes	Yes	No	No	No	No	No	No	No
No	no	Yes	No	No	No	No	No	No
No	no	Yes	No	No	No	No	No	No
No	no	Yes	No	No	No	No	No	No
No	People's a	No	No	No	Yes	No	No	No
Yes	It can be d	Yes	No	No	No	No	No	No
No	No	Yes	No	No	No	No	No	No
No	No	Yes	No	No	No	No	No	No
No	No	Yes	No	No	No	No	No	No
No	People's a	No	No	No	Yes	No	No	No
No	No barrier	Yes	No	No	No	No	No	No
No	No	Yes	No	No	No	No	No	No
No	No	Yes	No	No	No	No	No	No
No	No	Yes	No	No	No	No	No	No
No	No barrier	Yes	No	No	No	No	No	No
No	no	Yes	No	No	No	No	No	No
No	No	Yes	No	No	No	No	No	No
No	Difficult to	No	No	No	No	Yes	No	No
No	No	Yes	No	No	No	No	No	No
No	Language (	No	Yes	No	No	No	No	No
	The patien	No	No	No	No	Yes	No	No
No	Not availal							
No	No	Yes	No	No	No	No	No	No
No	Economic (	No	No	No	No	No	No	Yes

RASR barri	RASR barri	RASR barri	Impact of (	Impact of (	C-19 impac	C-19 impac	C-19 impac	C-19 impac
No	No	No	No, the iso	No	No	No	No	No
			The citizen	Yes	No	Yes	No	No
No	No	No	Lockdown	Yes	No	Yes	No	No
No	No	No	Yes, the cit	Yes	No	Yes	No	No
No	No	No	No	No	No	No	No	No
No	No	No	NO	No	No	No	No	No
No	No	No	Yes, the cit	Yes	No	Yes	No	No
No	No	No	Yes	Yes	Yes	No	No	No
Yes	No	No	Strengther	Yes	No	No	Yes	No
No	No	No	Lockdown	Yes	No	Yes	No	No
No	No	Yes	Affected b	Yes	Yes	No	No	No
No	No	No	Yes	Yes	Yes	No	No	No
No	No	Yes	yes	Yes	Yes	No	No	No
No	No	Yes	yes	Yes	Yes	No	No	No
No	No	Yes	Have a suc	Yes	Yes	No	No	No
			Yes, tracin	Yes	Yes	No	No	No
Yes	No	No	Yes	Yes	Yes	No	No	No
			Yes	Yes	Yes	No	No	No
No	No	Yes	yes	Yes	Yes	No	No	No
Yes	No	No	no	No	No	No	No	No
No	No	No	REDUCES I	Yes	Yes	No	No	No
No	No	No	no	No	No	No	No	No
Yes	No	No	yes, travel	Yes	No	No	No	Yes
No	No	No	Yes, the nu	Yes	No	Yes	No	No
No	No	No	no	No	No	No	No	No
No	No	Yes	Yes, the pa	Yes	No	No	No	No
Yes	No	No	Yes, travel	Yes	No	Yes	No	No
Yes	No	No	yes	Yes	Yes	No	No	No
Yes	No	No	yes, many	Yes	No	No	No	Yes
Yes	No	No	yes, helps	Yes	No	Yes	No	No
No	No	No	yes	Yes	Yes	No	No	No
Yes	No	No	Yes	Yes	Yes	No	No	No
			Controlling	Yes	No	Yes	No	No
No	No	No	Yes	Yes	Yes	No	No	No
No	No	No	Yes, due to	Yes	Yes	Yes	No	Yes
Yes	No	No	No	No	No	No	No	No
No	No	No	Yes, they h	Yes	No	Yes	No	No
No	No	No	Yes, they d	Yes	Yes	No	No	No
No	No	No	Yes, They I	Yes	No	Yes	No	No
No	No	No	Isolate anc	Yes	No	Yes	No	No
No	No	No	No	No	No	No	No	No
No	No	No	Yes, isolate	Yes	No	Yes	No	No
No	No	No	Yes, they li	Yes	No	Yes	No	No
No	No	No	Yes, the pe	Yes	No	No	No	No
No	No	No	Yes, the co	Yes	No	No	No	No
No	No	No	Yes, the cit	Yes	No	No	No	No
No	No	No	Yes, they d	Yes	No	No	No	No
No	No	No	The comm	Yes	No	No	No	No
No	No	No	Yes, it due	Yes	No	No	Yes	No

No	No	No	Yes, they is	Yes	No	Yes	No	No
No	No	Yes	Yes	Yes	Yes	No	No	No
No	No	No	No	No	No	No	No	No
No	No	No	no	No	No	No	No	No
No	No	No	no	No	No	No	No	No
No	No	No	Yes, quara	Yes	No	No	No	Yes
No	No	No	Yes	Yes	Yes	No	No	No
No	No	No	Yes	Yes	Yes	No	No	No
No	No	No	Yes	Yes	Yes	No	No	No
No	No	No	Yes, can't g	Yes	No	No	No	Yes
No	No	No	Yes, travel	Yes	Yes	No	No	No
No	No	No	Have. Unal	Yes	No	No	No	Yes
No	No	No	No effect	No	No	No	No	No
No	No	No	Yes, Can't i	Yes	No	No	No	Yes
No	No	No	Have	Yes	Yes	No	No	No
No	No	No	Travel rest	Yes	No	No	No	Yes
No	No	No	are not	No	No	No	No	No
No	No	No	Have	Yes	Yes	No	No	No
No	No	No	Have	Yes	Yes	No	No	No
No	No	No	During the	Yes	No	No	No	Yes
No	No	No	No	No	No	No	No	No
No	No	No	Can't gathe	Yes	No	No	No	Yes
			No	No	No	No	No	No
No	No	No	No	No	No	No	No	No
No	No	No	Yes becaus	Yes	No	Yes	No	No

C-19 impac	Suggestion	Suggestion	Number of	Total popu	Mobile pho	Quality of	Internet ac	Quality of
No	.							
No	.							
No	.							
No	.							
No	.							
No	.							
No	.							
No	.							
No	.							
No	.							
No	.							
No	.							
No	.							
No	.							
No	.							
No	.							
No	.							
No	.							
No	.							
No	.							
No	.							
No	.							
No	.							
No	.							
No	.							
Yes	.							
No	.							
No	.							
No	.							
No	.							
No	.							
No	.							
No	.							
No	.							
Yes	.							
No	.							
No	No	No suggest	230	1111	Yes	Yes	No	
No	No	No suggest	469	2649	Yes	Yes	Yes	Yes
No	Focus year		219	949	Yes	Yes	No	
No	IRS, sleepi	Providing l	204	942	Yes	Yes	Yes	Yes
No	Active case	Others	486	2649	Yes	Yes	Yes	Yes
No	Support th	Providing f	230	1250	Yes	Yes	Yes	Yes
No	Strenthern	Providing t	250	1003	Yes	Yes	Yes	Yes
Yes	Providing r	Providing l	234	905	Yes	Yes	Yes	Yes
Yes	distributio	Providing l	294	1227	Yes	Yes	Yes	Yes
Yes	Providing r	Providing l	234	905	Yes	Yes	Yes	Yes
Yes	Proposal th	Providing f	512	1027	Yes	Yes	Yes	Yes
Yes	Support th	Providing f	237	905	Yes	Yes	Yes	Yes
No	No	No suggest	204	942	Yes	Yes	No	

No	No	No suggest	230	1111	Yes	Yes	Yes	Yes
No	No	No suggest	331	850	Yes	Yes	Yes	Yes
No	Strenthern	Providing I	193	828	Yes	Yes	Yes	Yes
No	no	No suggest	320	1632	Yes	Yes	Yes	Yes
No	no	No suggest	193	828	Yes	Yes	Yes	Yes
No	Regular pr	Providing I	658	2069	Yes	Yes	Yes	Yes
No	Funding su	Providing f	1692	5041	Yes	Yes	Yes	Yes
No	curtain"		450	1567	Yes	No	Yes	Yes
No	No	No suggest	658	2069	Yes	Yes	Yes	Yes
No	No	No suggest	589	1895	Yes	Yes	No	
No	No	No suggest	3700	4263	Yes	No	Yes	No
No	Distributin	Providing IEC, preventive measur			Yes	Yes	Yes	Yes
No	Spraying cl	Providing I	103	368	Yes	Yes	Yes	Yes
No	Ky Du villa	Providing I	316	1368	Yes	Yes	Yes	No
No	Additional	Providing I	1260	4562	Yes	Yes	Yes	Yes
No	Level up m	Providing I	2770	9961	Yes	Yes	Yes	Yes
No	No	No suggest	2677	8600	Yes	Yes	Yes	Yes
No	No	No suggest	250	923	Yes	Yes	Yes	No
No	No	No suggest	2677	9691	Yes	Yes	Yes	Yes
No	Financial s	Providing I	286	955	Yes	No	No	
No	Support ra	Providing f	240	939	Yes	No	No	
No	Provide ne	Others	847	3377	No		No	
No	Extra fundi	Providing I	100	1000	Yes	No	Yes	No
No	Strengther	Providing t	240	939	No		No	
No	Financial s	Providing f	110	420	Yes	No	Yes	No

Average nu	Average nu	Method to	Notified by	Notified by	Notified by	Notified by	Notified by	Notified by
		.						
		.						
		.						
		.						
		.						
		.						
		.						
		.						
		.						
		.						
		.						
		.						
		.						
		.						
		.						
		.						
		.						
		.						
		.						
		.						
		.						
		.						
		.						
		.						
		.						
		.						
		.						
		.						
		.						
		.						
		.						
		.						
		.						
		.						
		.						
		.						
20	20	3	No	No	Yes	No	No	No
80	26	3	No	No	Yes	No	No	No
5	21	4	No	No	No	Yes	No	No
200	20	4	No	No	No	Yes	No	No
100	30	1	Yes	No	No	No	No	No
15	15	3	No	No	Yes	No	No	No
100	20	4	No	No	No	Yes	No	No
200	20	3	No	No	Yes	No	No	No
200	30	5	No	No	No	No	Yes	No
200	20	3	No	No	Yes	No	No	No
200	20	4.5	No	No	No	Yes	Yes	No
200	20	3	No	No	Yes	No	No	No
50	5	4	No	No	No	Yes	No	No

250	30	1,2,3,4,5	Yes	Yes	Yes	Yes	Yes	No
20	20	1	Yes	No	No	No	No	No
60	30	3	No	No	Yes	No	No	No
20	8	1.4	Yes	No	No	Yes	No	No
60	30	3	No	No	Yes	No	No	No
20	1	1.4	Yes	No	No	Yes	No	No
60	30	3	No	No	Yes	No	No	No
40	20	1	Yes	No	No	No	No	No
45	20	4	No	No	No	Yes	No	No
12	10	5	No	No	No	No	Yes	No
25	1	1	Yes	No	No	No	No	No
10	6	3	No	No	Yes	No	No	No
10	6	1	Yes	No	No	No	No	No
10	3	1	Yes	No	No	No	No	No
50	30	3	No	No	Yes	No	No	No
0		3	No	No	Yes	No	No	No
20	3	4	No	No	No	Yes	No	No
20	5	3	No	No	Yes	No	No	No
60	30	1.4	Yes	No	No	Yes	No	No
7	3	5	No	No	No	No	Yes	No
200	7	4	No	No	No	Yes	No	No
70	22	3	No	No	Yes	No	No	No
200	15	3	No	No	Yes	No	No	No
10	4	5	No	No	No	No	Yes	No
30	1	1	Yes	No	No	No	No	No

Involveme	If involved	If not invol	Follows th	Have been	Frequency	Perceived	Case inves	CI initiated
						.	for all case	Within 48
						.	for all case	After 48 ho
						.	for all case	After 48 ho
						.	for all case	Within 48
						.	for all case	Within 48
						.	not for all	Within 48
						.	not for all	Within 48
						.	for all case	Within 48
						.	not for all	After 48 ho
						.	not for all	Within 48
						.	for all case	After 48 ho
						.	not for all	After 48 ho
						.	for all case	Within 48
						.	for all case	Within 48
						.	not for all	After 48 ho
						.	for all case	After 48 ho
						.	for all case	Within 48
						.	for all case	Within 48
						.	for all case	Within 48
						.	for all case	Within 48
						.	for all case	After 48 ho
						.	for all case	Within 48
						.	for all case	Within 48
						.	not for all	After 48 ho
						.	not for all	After 48 ho
						.	for all case	After 48 ho
						.	not for all	After 48 ho
						.	for all case	After 48 ho
						.	for all case	After 48 ho
						.	for all case	Within 48
						.	for all case	After 48 ho
						.	for all case	After 48 ho
						.	for all case	Within 48
						.	for all case	Within 48
						.	for all case	Within 48
						.	for all case	After 48 ho
Yes, somet	Yes	Yes	Yes	Yes	Monthly	The health	for all case	After 48 ho
Yes, alway	Yes	Yes	Yes	Yes	Monthly	To confirm	for all case	After 48 ho
Yes, alway	Yes	Yes	Yes	Yes	Yearly	The difficu	for all case	After 48 ho
Yes, alway	No	Yes	Yes	Yes	Yearly	health staf	for all case	After 48 ho
Yes, alway	Yes	Yes	Yes	Yes	Monthly	Yes, to con	for all case	After 48 ho
Yes, alway	Yes	Yes	Yes	No		Dont know	not for all	Within 48
Yes, alway	Yes	Yes	Yes	Yes	Yearly	to be limit	not for all	After 48 ho
Yes, somet	Yes	Yes	Yes	Yes	Yearly	investigati	not for all	After 48 ho
Yes, alway	Yes	Yes	Yes	Yes	Yearly	Take blooc	for all case	After 48 ho
Yes, somet	Yes	Yes	Yes	No	Yearly	To control	for all case	After 48 ho
Yes, alway	Yes	Yes	Yes	Yes	Yearly	Take blooc	not for all	Within 48
No, never	Yes	Yes	Yes	Yes	Yearly	We mobili:	not for all	After 48 ho
Yes, alway	No	Yes	Yes	Yes	Yearly	no	for all case	After 48 ho

Yes, alway:	Yes	Yes	Yes	Yes	Yearly	Have know	for all case	Within 48 I
Yes, somet	Yes	Yes	Yes	Yes	Monthly	Yes	not for all (	Within 48 I
Yes, somet	No	Yes	Yes	No	Yearly	To confirm	for all case	Within 48 I
Yes, somet	Yes	Yes	Yes		Monthly	Dont know	not for all (	After 48 hc
Yes, alway:	Yes	Yes	Yes	Yes	Yearly	head of CH	not for all (	Within 48 I
Yes, somet	Yes	Yes	Yes	Yes	Yearly	Support fo	not for all (	Within 48 I
Yes, somet	Yes	Yes	Yes	Yes	Yearly	Importanc	for all case	Within 48 I
No, never	Yes		No		Quarterly	Commune	not for all (	Within 48 I
Yes, alway:	Yes		Yes	Yes	Yearly	In charge c	not for all (	After 48 hc
Yes, alway:	Yes		No	Yes	Six-monthl	don't know	for all case	After 48 hc
Yes, somet	Yes		Yes	No	Yearly	Capturing	not for all (	Within 48 I
Yes, somet	Yes		No	No		Consulting	for all case	Within 48 I
Yes, alway:	Yes		Yes	Yes	Yearly	Malaria co	not for all (	After 48 hc
Yes, alway:	Yes		Yes	Yes	Yearly	Communic	not for all (	Within 48 I
Yes, alway:	Yes		Yes	Yes	Yearly	Is it import	for all case	After 48 hc
Yes, somet	Yes		Yes	No		The role of	for all case	After 48 hc
Yes, somet	Yes		Yes	No		Blood colle	not for all (	Within 48 I
Yes, somet	Yes	Yes	Yes	Yes	Yearly	VHW	not for all (	Within 48 I
Yes, alway:	Yes		Yes	Yes	Yearly	Is it import	for all case	After 48 hc
Yes, alway:	Yes			Yes	Yearly	Take test, (	for all case	After 48 hc
No, never		Yes	Yes	No		Importanc	for all case	Within 48 I
Yes, alway:	Yes		Yes	Yes	Six-monthl	Timely sup	for all case	After 48 hc
Yes, somet	Yes		Yes	Yes	Quarterly	In charge c	for all case	Within 48 I
Yes, somet	No	Yes	Yes			.	not for all (	Within 48 I
Yes, somet	No	Yes	Yes	Yes	Yearly	VHW only	not for all (	After 48 hc

RECODE of
Yes
Yes
Yes
Yes
Yes
No
Yes
No
Yes

Yes
Yes
Yes
Yes
Yes
Yes
Yes
Yes
Yes
Yes
Yes
Yes
Yes
Yes
Yes
Yes
Yes
Yes
Yes
Yes
Yes
Yes
No
Yes
Yes
Yes
