# Supplementary material for: Facilitators, barriers and acceptability of malaria reactive surveillance and response strategies in Vietnam: a mixed-methods study
Source: BMJ Public Health. 2024 Dec 16;2(2):e000961. doi: 10.1136/bmjph-2024-000961 (PMC11816204; doi:10.1136/bmjph-2024-000961)
Supplement: online supplemental file 6 [file bmjph-2-2-s006.pdf]

RESEARCH PROGRAM TITLE

Optimizing reactive surveillance and response strategies to achieve malaria elimination across the Greater Mekong Subregion: A mixed-methods evaluation study

SPONSORED BY

Burnet Institute

RESEARCH TEAM

Principal Investigators

Dr Win Han Oo, MBBS, MPH (Global Health)

Professor Freya Fowkes, BSc (Hons), MSc, DipLSHTM, DPhil (Oxon)

Co-Investigators

Dr Julia Cutts, BA/BSc (Hons), MPH, PhD

Mr Paul Agius, BA, Grad Dip Soc, MSc

Dr Aung Thi, MBBS, MPH

Dr Wint Phyo Than, MBBS, MPH

Dr Nay Yi Yi Linn, MBBS, M.Med.Sc (Public Health), D.T.M&H

Dr Kyawt Mon Win, MBBS, MPH

Dr. Siv Sovannaroth, MD, CNM

Dr Phoutnalong Vilay, CMPE

Dr Tran Thanh Duong, NIMPE

Dr Win Htike, MBBS, MPH

Bangyuan Wang, MPA, HPA

Mr. Lun Sovanda, BPH, BA, HPA

Dr Thet Lynn, MBBS, MMedSc GH, HPA

Ms Nguyen Thi Minh Nguyet, BSc, MEBF

Dr Katherine O'Flaherty, BSc (Hons), PhD

Ms Ellen Kearney, BA BSci (Hons)

Dr Galau Naw Hkawng, MBBS

Dr Kaung Myat Thu, MBBS, MMedSc (P&TM)

Dr May Chan Oo, MBBS, MPH, PhD

Dr Aung Pyae Phyo, MBBS, PhD

Mr Zhang Shuhao, BA, MA

FUNDED BY

Regional Artemisinin-Resistance Initiative 3 – Elimination (RAI3E) Grant funded by United Nations Office for Project Services (UNOPS)

CONFIDENTIAL

This document is confidential and the property of Burnet Institute (Melbourne and Myanmar offices). No part of it may be transmitted, reproduced, published, or used without prior written authorization from the institutions.

STATEMENT OF COMPLIANCE

This document is a protocol for an operational research study. The study will be conducted in compliance with all stipulations of this protocol, the conditions of ethics committee approval, the NHMRC National Statement on Ethical Conduct in Human Research (2007) and the Note for Guidance on Good Clinical Practice (CPMP/ICH-135/95).

1. Contact details

Investigator	Address	Email
**Dr Win Han Oo**	Burnet Institute Myanmar<br>No. 226, 4th Floor, Wizaya Plaza, U Wisara Road, Bahan Township,11201 Yangon, Myanmar	winhan.oo@burnet.edu.au
**Professor Freya Fowkes**	Burnet Institute<br>85 Commercial Rd, Melbourne, VIC 3004, Australia	freya.fowkes@burnet.edu.au
**Dr Julia Cutts**	Burnet Institute<br>85 Commercial Rd, Melbourne, VIC 3004, Australia	julia.cutts@burnet.edu.au
**Mr Paul Agius**	Burnet Institute<br>85 Commercial Rd, Melbourne, VIC 3004, Australia	paul.agius@burnet.edu.au
**Dr Aung Thi**	Myanmar National Malaria Control Programme<br>Department of Public Health, Ministry of Health and Sports, Nay Pyi Taw, Myanmar	aungthi08@gmail.com
**Dr Wint Phyo Than**	Myanmar National Malaria Control Programme<br>Department of Public Health, Ministry of Health and Sports, Nay Pyi Taw, Myanmar	wintphyothan@gmail.com
**Dr Nay Yi Yi Linn**	Vector Borne Diseases Control Unit<br>Department of Public Health, Ministry of Health and Sports, Nay Pyi Taw, Myanmar	nayyiyilinn@gmail.com
**Dr Kyawt Mon Win**	Myanmar National Malaria Control Programme<br>Department of Public Health, Ministry of Health and Sports, Nay Pyi Taw, Myanmar	kyawtmonwin@gmail.com
**Dr Siv Sovannaroth**	National Center for Parasitology, Entomology and Malaria Control (CNM);<br>Ministry of Health, Cambodia 477, Corner street 92, Trapeng Svay village, Sangkat Kouk Khleang, Khan Sen Sok, Phnom Penh	sivsovannaroths@gmail.com
**Dr Phoutnalong Vilay**	Center for Malaria, Parasitology and Entomology (CMPE);<br>Ministry of Health, Vientiane capital, Lao PDR	phoutnalongvilay@gmail.com

**Dr Tran Thanh Duong**	Managing Director<br>National Institute Of Malariology, Parasitology And Entomology (NIMPE), Vietnam	tranthanhduong@hotmail.com
**Dr Win Htike**	Burnet Institute Myanmar<br>No. 226, 4th Floor, Wizaya Plaza, U Wisara Road, Bahan Township,11201 Yangon, Myanmar	win.htike@burnet.edu.au
**Mr Bangyuan Wang**	Health Poverty Action<br>Grd Floor, 31-33 Bondway, London, SW8 1SJ, United Kingdom	b.wang@healthpovertyaction.org
**Mr Lun Sovanda**	Health Poverty Action<br>#TR16-106B, Orkide Village, Street Daliya, Sangkat Ou Bek Ka Orm, Khan Sen Sok, Phnom Penh, Cambodia	s.lun@healthpovertyaction.org
**Dr Thet Lynn**	Health Poverty Action<br>Unit 18, House No. 362, Saysettha District, Vientiane Capital, Lao PDR	T.Lynn@healthpovertyaction.org
**Ms Nguyen Thi Minh Nguyet**	Vietnam Country Office<br>245 Luong The Vinh, Trung Van, Nam Tu Liem, Hanoi	N.Nguyet@healthpovertyaction.org
**Dr Katherine O'Flaherty**	Burnet Institute<br>85 Commercial Rd, Melbourne, VIC 3004, Australia	katherine.oflaherty@burnet.edu.au
**Ms Ellen Kearney**	Burnet Institute<br>85 Commercial Rd, Melbourne, VIC 3004, Australia	ellen.kearney@burnet.edu.au
**Dr Galau Naw Hkawng**	Burnet Institute Myanmar<br>No. 226, 4th Floor, Wizaya Plaza, U Wisara Road, Bahan Township,11201 Yangon, Myanmar	galaunaw.hkawng@burnet.edu.au
**Dr Kaung Myat Thu**	Burnet Institute Myanmar<br>No. 226, 4th Floor, Wizaya Plaza, U Wisara Road, Bahan Township,11201 Yangon, Myanmar	kaungmyat.thu@burnet.edu.au

**Dr May Chan Oo**	Burnet Institute Myanmar No. 226, 4th Floor, Wizaya Plaza, U Wisara Road, Bahan Township,11201 Yangon, Myanmar	maychan.oo@burnet.edu.au
**Dr Aung Pyae Phyo**	Myanmar Oxford Clinical Research Unit, Yangon, Myanmar	AungPyaePhyo@tropmedres.ac
**Mr Zhang Shuhao**	Health Poverty Action, Kunming, China	zhangshuhao@healthpovertyaction.org.cn

2. Table of Contents

1. Contact details....ii
2. Table of Contents....i
3. Composition of Research Team....v
4. Glossary of Abbreviations and Terms....xi
5. Overview of Research Program....xii
6. Introduction....1
6.1. Malaria....1
6.2. Recent global achievements in malaria control and elimination....1
6.3. Malaria in the Greater Mekong subregion....3
6.4. Enhancing surveillance in the transition from malaria control to elimination....4
6.5. Reactive surveillance and response activities in elimination settings....5
6.5.1. Case and focus investigations....5
6.5.2. Focus based surveillance and response activities....7
6.6. Reactive surveillance and response strategies in the Greater Mekong Subregion....10
6.6.1. China's 1-3-7 strategy....10
6.7. Rationale for a region-wide study of reactive surveillance and response strategies in the GMS....11
7. Aims and Objectives....12
7.1. Aim of study....12
7.2. Research Questions....13
7.3. Research Objectives....14
7.4. Study Outcomes....14
8. Research Design and Methods....15
8.1. Mixed-methods study design....15
Part A: Systematic review....16
Part B: Questionnaire survey of malaria program stakeholders....16
Part C: Qualitative assessment (interviews and focus group discussions) of malaria program stakeholders, frontline malaria service providers and beneficiaries....16
Part D: Secondary data analysis....17
8.2. Part A: Systematic Review of published and grey literature on malaria reactive surveillance and response strategies....20
8.2.1. Administrative Information....20

8.2.2. Systematic review protocol 21
Aim: 21
Review questions: 21
Objectives: 22
Methodology 22
Search methods for identification of studies 22
Criteria for considering studies for this review 23
Types of studies 23
Quantitative and mixed-methods studies 23
Qualitative studies 24
Exclusions 24
Study populations 24
Interventions of interest 24
Outcomes of interest 25
Selection of studies 25
Quality and Risk of bias assessment 25
Data extraction 26
Data management, analysis, and synthesis 26
Quantitative studies 26
Qualitative studies 27
8.2.3. Overall outcomes of systematic review 27
8.3. Part B: Questionnaire survey of malaria program field staff, field supervisors, and frontline service providers 28
8.3.1. Study design 28
8.3.2. Objectives 28
8.3.3. Outcomes 28
8.3.4. Study settings and study sites 29
8.3.5. Study populations and sample size 29
Questionnaire 1 29
Questionnaire 2 30
8.3.6. Sampling Strategy and Participant recruitment 32
8.3.7. Study procedures, data collection methods and tools 33
Other considerations for study procedures 34
8.3.8. Data processing, management and analysis 34

8.4. Part C: Qualitative assessment (interviews and focus group discussions) with malaria program stakeholders, service providers and beneficiaries 35
8.4.1. Study design 35
8.4.2. Objectives 35
8.4.3. Outcomes 36
8.4.4. Study setting and study sites 36
8.4.5. Study population and sample size 36
Semi-structured interviews with higher and middle level malaria program stakeholders 37
Focus group discussion with lower-level malaria program stakeholders 38
Focus group discussion with frontline malaria service providers 39
Focus group discussion with mobile and migrant populations 40
8.4.6. Sampling strategy 41
8.4.7. Participant recruitment 41
8.4.8. Study procedures, data collection methods and tools 42
Semi-structured interviews 42
Focus group discussions 43
Other considerations for study procedures 44
Pilot testing of interview and FGD topic guides 44
8.4.9. Data processing and management 45
8.4.10. Qualitative data analysis 45
8.5. Part D: Secondary data analysis 45
8.5.1. Study design 45
8.5.2. Objectives 46
8.5.3. Outcomes 46
8.5.4. Study setting, study sites and study population 47
8.5.5. Data retrieval, processing, management and analysis 47
9. Results dissemination and future directions 48
10. Ethical considerations 49
10.1. Ethics review 49
10.2. Informed consent 49
Written informed consent 49
Waiver of consent 50
10.3. Risks and Benefits 52

10.3.1. Risks 52
10.3.2. Benefits 52
10.3.3. Confidentiality 53
11. Research Timeline 54
12. Capability statement 55
13. Appendices 62
Appendix A. Outcomes 62
Appendix B. Participant selection tools for Part B: Questionnaire surveys 62
Screening tool for states/regions/provinces eligible for Questionnaire 1 of the study 62
Screening tool for malaria program stakeholders eligible for Questionnaire 1 of the study 62
Screening tool for states/regions/provinces eligible for Questionnaire 2 of the study 62
Screening tool for frontline malaria service providers eligible for Questionnaire 2 of the study 62
Appendix C. Questionnaires for surveying the malaria program stakeholders 62
Questionnaire 1 for surveying the malaria program stakeholders responsible for managing or supervising field reactive surveillance and response activities 62
Questionnaire 2 for surveying frontline malaria service providers 62
Appendix D. Topic guides for semi-structured interviews and focus group discussions 62
Topic guide for semi-structured interview with malaria program stakeholders responsible for designing and overviewing malaria reactive surveillance and response policies and strategies 62
Topic guide for focus group discussion with malaria program stakeholders responsible for managing or supervising field reactive surveillance and response activities 62
Topic guide for focus group discussion with frontline malaria service providers 62
Topic guide for focus group discussion with mobile and migrant populations 62
Appendix E. List of variables to be collected from the national malaria control programs for secondar data analysis (Part D) 62
Appendix F. Data transfer agreement 62
Appendix G. Participant information and consent forms 62
Participant information and consent form for questionnaire survey of the malaria program stakeholders responsible for managing or supervising field reactive surveillance and response activities 63
Appendix H. Outline of data flow 63
Appendix I. Approved budget 63

3. Composition of Research Team

No.	Name & designation	Role	Responsibilities
1	**Dr Win Han Oo**<br>Senior Program Manager,<br>Burnet Institute	Co-Principal Investigator	As a principal investigator, Dr Win Han Oo will contribute to the study protocol and finalization of all study documents. Dr Win Han Oo will coordinate the trainings and activities of the research and will liaise with policy makers and stake holders in Myanmar, as well as with the technical staff of Burnet Institute. Dr Win Han Oo will play a role in the analysis and communication of data relating to the research project. He will also provide in assessing the eligibility of the villages, recruiting the study participants and obtaining their consent.
2	**Prof. Freya Fowkes**<br>Deputy Program Director,<br>Maternal and Child Health<br>Head, Malaria and Infectious Disease Epidemiology,<br>NHMRC Fellow,<br>Burnet Institute	Co-Investigator	As a co-investigator, Professor Freya Fowkes will contribute to the study protocol and finalization of all study documents. Professor Freya Fowkes will coordinate the training and activities of the research coordinators, and will liaise with policy makers, and senior technical staff in Melbourne. Professor Freya Fowkes will play a key role in the analysis and communication of data relating to the project
3	**Dr Julia Cutts**<br>Senior Research Officer,<br>Malaria and Infectious Disease Epidemiology Group,<br>Burnet Institute	Co-Investigator	Dr Julia Cutts will be involved in initial study design, writing the study protocol and preparing study documents for this project. Dr Julia Cutts will contribute to laboratory-based assays, analysis, and communication of data relating to the project.
4	**Mr Paul Agius**<br>Burnet Senior Fellow &<br>Applied Statistician,<br>Adjunct Senior Research Fellow (Monash University),<br>Research Fellow (La Trobe University),<br>Burnet Institute	Co-Investigator	Mr Paul Agius will provide statistical, methodological and study design expertise to this project and will be responsible for supervising the analysis of trial data.

5	**Dr Aung Thi**<br>Programme Manager and Director (Malaria), National Malaria Control Programme, Ministry of Health and Sports	Principal Investigator	Dr Aung Thi will provide technical support, coordinate the research activities with malaria teams of Ministry of Health and Sports. He will also provide in assessing the eligibility of the villages, recruiting the study participants and obtaining their consent.
6	**Dr Kyawt Mon Win**<br>Assistant Director (Malaria), National Malaria Control Programme, Ministry of Health and Sports	Co-Investigator	Dr Kyawt Mon Win will provide technical support in protocol development, coordinate the research activities with malaria teams of Ministry of Health and Sports. She will also take the role of coordination between research team and State and Regional VBDC teams during implementation and will also contribute to data collection, analysis and report writing.
7	**Dr. Siv Sovannaroth, CNM**<br>Malaria Program Manager/ Chief of Technical, National Center for Parasitology, Entomology and Malaria Control (CNM); Ministry of Health, Cambodia	Co-Investigator	Dr. Siv Sovannaroth, will provide technical support, coordinate the research activities with CNM and public health teams at central, provincial, district, primary health facility and community levels. He will also provide in assessing the eligibility of the study sites, recruiting the study participants and obtaining their consent.
8	**Dr Phoutnalong Vilay,**<br>Vice of unit, Center for Malaria, Parasitology and Entomology (CMPE); Ministry of Health, Vientiane capital, Lao PDR	Co-Investigator	Dr. Phoutnalong will provide technical support, coordinate the research activities with CMPE and public health teams at central, provincial, district, primary health facility and community levels. He will also provide in assessing the eligibility of the study sites, recruiting the study participants and obtaining their consent.
9	**Dr Tran Thanh Duong,**<br>Managing Director National Institute Of Malariology, Parasitology And Entomology Vietnam	Co-Investigator	Dr Tran Thanh Duong will provide technical support, coordinate the research activities with NIMPE and public health teams at central, provincial, district, primary health facility and community levels. He will also provide in assessing the eligibility of the study sites, recruiting the study participants and obtaining their consent.

10	**Dr Win Htike**<br>Program Manager (Malaria), Burnet Institute	Co-Investigator	Dr Win Htike will liaise between Myanmar-based operation support team, and research team in Burnet Institute Melbourne. His main role is project management which includes financial monitoring and donor reporting. He will also provide technical support in study design, protocol development, analysis and report writing.
11	**Mr Bangyuan Wang**<br>Head of Programme, Asia, Health Poverty Action	Co-Investigator	Mr Bangyuan Wang will be a co-investigator in the HPA team. As Head of Asia Programmes, he will have overall responsibility for HPA operations related to the research across Cambodia, Laos, Myanmar and Vietnam as well as operations in the UK. This includes (but is not limited to) supervision of country offices, overall financial and programmatic oversight.
12	**Mr Lun Sovanda**<br>Malaria Program Officer, Health Poverty Action, Cambodia	Co-Investigator	Mr Lun Sovanda will be a co-investigator in the HPA team. As Program Officer in Cambodia, he will have overall responsibility for HPA operations related to the research in Cambodia. This includes (but is not limited to) supervision of the country office and field offices, in-country financial and programmatic oversight, relations with the CNM including securing of MOUs and other agreements, sector partners and also with related projects, as well as day to day coordination with BI on research activities and scheduling.
13	**Dr Thet Lynn**<br>Country Director, Health Poverty Action, Lao PDR	Co-Investigator	Dr Thet Lynn will be a co-investigator in the HPA team. As Country Director in Laos, he will have overall responsibility for HPA operations related to the research in Laos. This includes (but is not limited to) supervision of the country office and field offices, in-country financial and programmatic oversight, relations with the CMPE including securing of MOUs and other agreements, sector partners and also with related projects, as well as day to day coordination with

			BI on research activities and scheduling.
14	**Ms Nguyen Thi Minh Nguyet**<br>Country Director,<br>Health Poverty Action,<br>Vietnam	Co-Investigator	Ms Nguyen Thi Minh Nguyet will be a co-investigator in the HPA team. As Team Leader in Vietnam, she will have overall responsibility for HPA operations related to the research in Vietnam. This includes (but is not limited to) supervision of the country office and field offices, in-country financial and programmatic oversight, relations with the NIMPE including securing of MOUs and other agreements, sector partners and also with related projects, as well as day to day coordination with BI on research activities and scheduling.
15	**Dr Katherine O'Flaherty**<br>Postdoctoral Scientist,<br>Malaria and Infectious Diseases Epidemiology,<br>Burnet Institute	Co-Investigator	Dr Katherine O'Flaherty will contribute to coordination of sample collection and transport, laboratory-based assays, analysis, and communication of data relating to the project.
16	**Ms. Ellen Kearney**<br>PhD Student,<br>Malaria and Infectious Diseases Epidemiology Group, Burnet Institute	Co-Investigator	Ms Ellen Kearney will be a co-investigator in the research team and will function as a member of the Melbourne research team. She will support the PI and in country research team. Ellen's key roles and responsibilities will involve liaising with researchers at the Burnet Institute and Burnet Institute Myanmar to develop the protocol and oversee the Melbourne based ethics submission.
17	**Dr Galau Naw Hkawng**<br>Technical Specialist (Malaria), Burnet Institute	Co-Investigator	Dr Galau Naw Hkawng will be a co-investigator in the research team. The detailed tasks assigned include contribution in research protocol development including the data collection tools, field management and supervision of data collection process, and contribution in data analysis, reporting and manuscript writing. He will also provide in assessing the eligibility of the villages, recruiting the study participants, and obtaining their consent.
18	**Dr Kaung Myat Thu**<br>Technical Specialist (Malaria), Burnet Institute	Co-Investigator	Dr Kaung Myat Thu will be a co-investigator in the research team. The detailed tasks assigned include

			contribution in research protocol development including the data collection tools, field management and supervision of data collection process, and contribution in data analysis, reporting and manuscript writing. He will also provide in assessing the eligibility of the villages, recruiting the study participants, and obtaining their consent.
19	**Dr May Chan Oo**<br>Project Manager (Malaria), Burnet Institute	Co-Investigator	Dr May Chan Oo will be a co-investigator in the research team. The detailed tasks assigned include contribution in research protocol development including the data collection tools, field management and supervision of data collection process, and contribution in data analysis, reporting and manuscript writing. He will also provide in assessing the eligibility of the villages, recruiting the study participants, and obtaining their consent.
20	**Dr Aung Pyae Phyo**<br>Post-doctoral research fellow, Burnet Institute<br>Senior Clinician Scientist Myanmar Oxford Clinical Research Unit (MOCRU), Myanmar and Shoklo Malaria Research Unit (SMRU), Thailand	Co-Investigator	Dr Aung Pyae Phyo will act as the focal person for implementation of field research activities in Thailand, including stakeholder engagement. field management and supervision of qualitative data collection. He will coordinate with the Bureau of Vector Borne Diseases (BVD) staff in Thailand and contribute to protocol development, data analysis, reporting and manuscript writing.
21	**Mr Zhang Shuhao**<br>Program Officer, Health Poverty Action (China)	Co-investigator	Mr Zhang Shuhao will be a co-investigator in the HPA team and will act as the focal person for implementation of field research activities in China, including stakeholder engagement. field management and supervision of qualitative data collection. He will coordinate with the Yunnan Institute of Parasitic Diseases (YIPD) staff in China and contribute to protocol development, data analysis, reporting and manuscript writing.
22	**Dr Wint Phyo Than**	Co-Investigator	Dr Wint Phyo Than will provide technical support in protocol development, coordinate the research

	Deputy Director and Prgramme Manager for National Malaria Control Programme, Ministry of Health and Sports, Myanmar		activities with malaria teams of Ministry of Health and Sports. She will also take the role of coordination between research team and State and Regional VBDC teams during implementation and will also contribute to data collection, analysis and report writing.
23	**Dr Nay Yi Yi Linn**<br>Deputy Director and Programme Manager for Dengue and Lymphatic Filariasis under Vector Borne Disease Control Unit (VBDC), Ministry of Health and Sports**,** Myanmar	Co-Investigator	Dr Nay Yi Yi Linn will provide technical support in protocol development, coordinate the research activities with VBDC teams of Ministry of Health and Sports. She will also take the role of coordination between research team and State and Regional VBDC teams during implementation and will also contribute to data collection, analysis and report writing.

4. Glossary of Abbreviations and Terms

- ACD – Active Case Detection
- API – Annual Parasite Incidence
- BCC – Behaviour Change Communication
- BI – Burnet Institute
- BIMM – Burnet Institute Myanmar
- BVD – Bureau of Vector Borne Diseases, Thailand
- CI – Confidence Interval
- CHW – Community-based Health Worker
- CMPE – Centre for Malaria Parasitology and Entomology, Lao PDR
- CNM – Cambodia National Malaria Center, Cambodia
- FGD – Focus Group Discussion
- GFATM/ GF – The Global Fund to Fight AIDS, Tuberculosis and Malaria
- GMS – Greater Mekong Sub-region
- GTS – Global Technical Strategy for Malaria (2016 – 2030)
- IP – Implementing Partner
- IRS – Indoor Residual Spraying
- Lao PDR – Laos People's Democratic Republic
- LLIN – Long Lasting Insecticidal Net
- MoHS – Myanmar Ministry of Health and Sports
- NIMPE – National Institute of Malariology, Parasitology and Entomology, Vietnam
- NMCP – National Malaria Control Programme, China/Myanmar *(will specify)*
- PCD – Passive Case Detection
- *Pf* – *P. falciparum*
- *P.v.* – *Plasmodium vivax*
- RAI3E – Regional Artemisinin-Resistance Initiative 3 – Elimination
- RDT – Rapid Diagnostic Test
- RACD – Reactive case detection
- RASR – Reactive surveillance and response Strategies
- SEA – South East Asia
- UNOPS – United Nations Office for Project Services
- VBDC – Vector Borne Disease Control
- WHO – World Health Organization
- YIPD – Yunnan Institute of Parasitic Diseases

5. Overview of Research Program

Aim	To conduct a formative assessment of current malaria reactive surveillance and response strategies in the malaria elimination programs of GMS countries namely Cambodia, Lao PDR, Myanmar, Thailand and Vietnam, and Yunnan, China and identify how these strategies may be optimised and synthesised for improving their quality, effectiveness, and coverage in the context of existing national health systems for achieving regional malaria elimination targets
Objectives	1. To describe the malaria reactive surveillance and response strategies currently being implemented in GMS countries (Cambodia, Lao PDR, Myanmar, Thailand, Vietnam, Yunnan Province, China)<br>2. To investigate the knowledge, attitudes and practices of GMS malaria program stakeholders regarding current malaria reactive surveillance and response strategies, including the adherence of the program stakeholders to the strategies<br>3. To identify barriers and enablers to the successful implementation of reactive surveillance and response strategies in the GMS<br>4. To determine the effectiveness of the current malaria reactive surveillance strategies in GMS countries, including the positive case yield of reactive case detection (RACD) strategies<br>5. To investigate the timeliness and completeness of reactive surveillance and response activities in GMS countries<br>6. To explore the acceptability of reactive surveillance and response strategies to malaria program stakeholders and beneficiaries and the feasibility of successful implementation of time-bound reactive surveillance and response strategies in GMS countries<br>7. To explore how current malaria reactive surveillance and response strategies can be adapted to overcome existing barriers and improve their effectiveness in malaria elimination in the GMS countries, including those measures regarding the participation of CHWs in the strategies and optimization of the strategies for the MMPs
Design	A region-wide formative assessment of malaria reactive surveillance and response strategies currently being used in GMS, with a focus on target schedules of case notification, case investigation, foci investigation, and response (CIFIR) activities, and persons/departments responsible for these activities.<br>This formative assessment will include four components:<br>**Part A:** Systematic review of published and grey literature on malaria reactive surveillance and response strategies<br>**Part B:** Questionnaire survey of malaria program field staff, field supervisors, and frontline service providers<br>**Part C:** Qualitative assessment (interviews and focus group discussion) of malaria program stakeholders and beneficiaries<br>**Part D:** Secondary data analysis of malaria reactive surveillance and response data from GMS countries
Study Populations	**Part B & C:** Malaria program stakeholders, field staff, field supervisors, frontline malaria service providers, mobile and migrant populations in GMS countries

	**Part D:** Malaria case data from 2019 and 2020 calendar years in GMS countries
Study Sites	Five GMS countries (Cambodia, Lao PDR, Myanmar, Thailand and Vietnam) and one province (Yunnan, China)
Study duration	1 year and 9 months (1st January 2021 to 30th September 2022)
Outcomes	8. Detailed description of malaria reactive surveillance and response strategies currently being implemented in GMS countries namely Cambodia, Lao PDR, Myanmar, Thailand, Vietnam and Yunnan Province, China<br>9. Levels of knowledge, attitudes and practices of malaria program stakeholders in the GMS countries regarding different activities of their current malaria reactive surveillance and response strategies<br>10. Estimated proportion of malaria cases that result in timely and complete case notification, case and focus investigations, and response activities according to the current national reactive surveillance and response strategies in the five GMS countries and Yunnan Province, China<br>11. Estimated positive case yield of reactive case detection in each GMS country<br>12. Views and perspectives different malaria program stakeholders and beneficiaries in GMS countries on the acceptability of the current malaria reactive surveillance and response strategies<br>13. Identified policy, strategic, and operational barriers and enablers in successful implementation of the current malaria reactive surveillance and response strategies in GMS countries<br>14. A matrix of opinions and suggestions on how the current malaria reactive surveillance and response strategies in GMS countries can be optimized so that the existing barriers are overcome, effectiveness in malaria elimination is improved, and the strategies fit well into current elimination settings in the region<br>15. A matrix of opinions and suggestions on how participation of CHWs can be improved in these reactive surveillance and response activities and how malaria reactive surveillance and response activities can be improved for the MMPs in GMS countries
Budget	US $500,000

6. Introduction

6.1. Malaria

Malaria is a life-threatening disease caused by *Plasmodium* parasites transmitted to the human host by the bites of infected female *Anopheles* mosquitoes [1]. In 2019, approximately 229 million malaria cases and 409,000 malaria deaths were recorded worldwide, with the majority of both cases and deaths occurring in Africa (>90%) [1]. Of the five *Plasmodium* species to infect humans (*P. falciparum, P. vivax, P. malariae*, *P. ovale*, and *P. knowlesi), P. falciparum* and *P. vivax* are responsible for the greatest burden of disease worldwide [2]. While *P. falciparum* causes the vast majority of malaria-related deaths, *P. vivax* infections are particularly challenging to prevent and treat because the parasite can remain in a dormant form in the liver causing repeated relapses over months or years [1, 3]. Clinical manifestations of uncomplicated malaria include headache, lassitude, fatigue, abdominal discomfort, muscle and joint aches, fever, chills, perspiration, anorexia, vomiting and worsening malaise. If effective medicines are not given, or treatment is delayed, life-threatening severe malaria may develop, with manifestations including cerebral malaria, metabolic acidosis, severe anaemia, hypoglycaemia, acute renal failure, and acute pulmonary oedema [2].

6.2. Recent global achievements in malaria control and elimination

Over the last two decades increased investment in malaria control programs has led to dramatic decreases in malaria cases and deaths, but in the last five years the rate of progress has slowed [1]. Global malaria case incidence declined by 27% between 2000 and 2015 (from 80 to 58 cases per 1000 population at risk), and by less than 2% between 2015 and 2019 (to 57 cases per 1000 population at risk) [1].

The World Health Organization (WHO) developed a Global Technical Strategy for Malaria 2016–2030 (GTS), endorsed by the World Health Assembly in 2015 [3] which set the target of reducing the global malaria burden by 90% by 2030, compared with 2015, and to eliminate malaria in at least 35 countries by 2030 [3], where malaria elimination is defined as the *"interruption of local transmission (reduction to zero incidence of indigenous cases) of a specified malaria parasite species in a defined geographical area as a result of deliberate activities; continued measures to prevent re-establishment of transmission are required"* [4]. The GTS provides technical guidance to countries and development partners and recommends

countries set their own national or subnational targets and tailor the intervention packages for use in different settings within a country [2]. Globally, the number of countries that were malaria endemic in 2000 and that reported fewer than 10,000 malaria cases increased from 26 in 2000 to 46 in 2019. In the same period, the number of countries with fewer than 100 indigenous cases increased from six to 27 [1]. Paraguay and Uzbekistan have received WHO certification of elimination in 2018, Algeria and Argentina in early 2019, and China and El Salvador had no indigenous malaria cases for a third consecutive year and have made a formal request for certification [1]. The GTS is an overarching approach based on three pillars and two supporting elements (**Figure 1**). The GTS promotes universal coverage of malaria prevention, diagnosis and treatment services (Pillar 1), accelerated efforts towards elimination and attainment of malaria-free status (Pillar 2), and the transformation malaria surveillance into a core intervention (Pillar 3). These pillars are based on the two supporting elements of harnessing innovation and expanding research and strengthening the enabling environment [4].

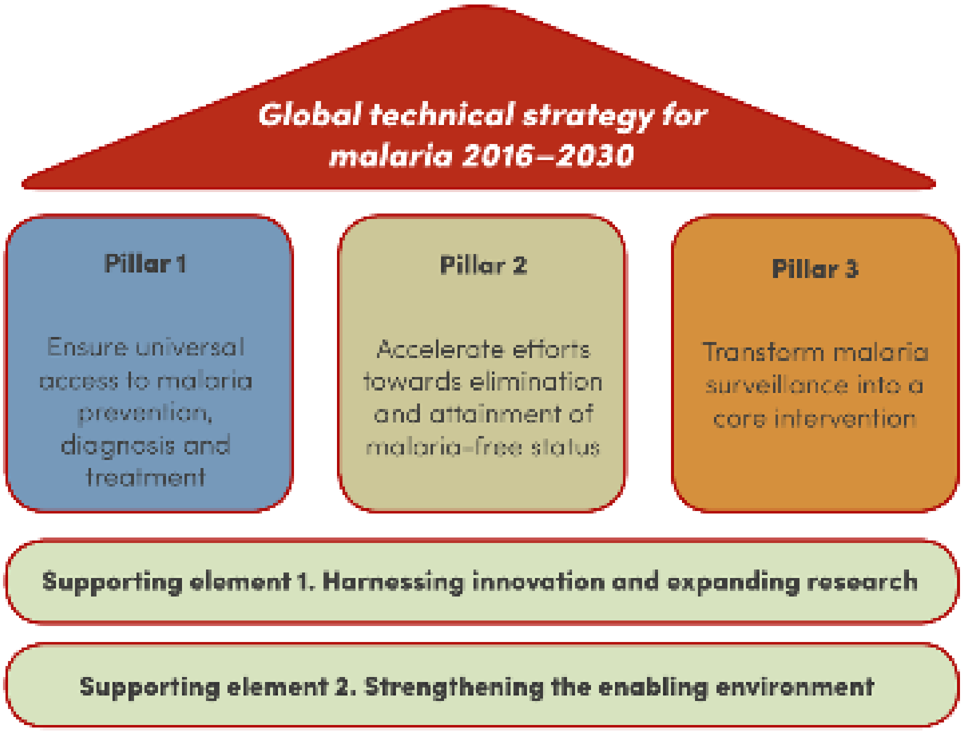

Figure 1. Global Technical Strategy framework: pillars and supporting elements [2]

6.3. Malaria in the Greater Mekong subregion

The Greater Mekong subregion (GMS), comprised of Cambodia, China (Yunnan Province), Lao People's Democratic Republic (Lao PDR), Myanmar, Thailand and Vietnam, has complex malaria epidemiology characterized by geographical heterogeneity in disease distribution [5]. Between 2000 and 2019, *P. falciparum* malaria cases fell by 97% in the GMS, while all malaria cases fell by 90% [1]. Of the 239,000 malaria cases reported in 2019, 65,000 were *P. falciparum* cases [1]. However, progress towards malaria elimination is threatened by artemisinin resistant *Plasmodium falciparum*, which first emerged in Cambodia and has now spread, or emerged independently, throughout the Greater Mekong Subregion [6-8]. Artemisinin resistance is characterised by delayed parasite clearance following treatment with artemisinin-based monotherapy or with an artemisinin-based combination therapy (ACT) and has been associated with non-synonymous mutations in the propeller region of the *P. falciparum kelch13* (K13) gene [7, 9]. Concerns around artemisinin resistance led to a 2015 commitment by all Ministers of Health in GMS countries and the WHO to adopt a subregional strategy aiming to eliminate *P. falciparum* malaria by 2025 and all species of human malaria by 2030 [8, 9].

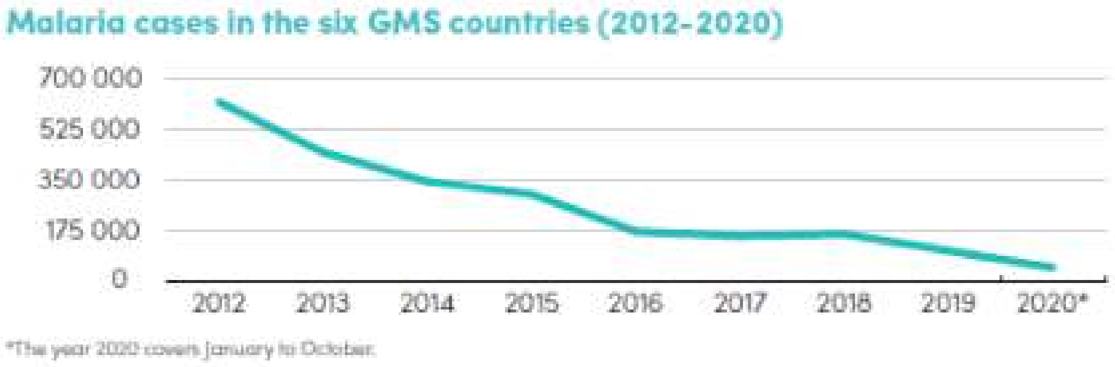

Figure 2. Malaria incidence in six GMS countries (2012 – 2020) [10]

In the GMS, remaining malaria caseloads are concentrated in hard-to-reach areas, often along international borders and in forest and forest fringe communities, presenting challenges for targeting of interventions to those at risk [10, 11]. Forest-going and other mobile and migrant populations are susceptible to malaria because they tend to work and live in remote areas where

conditions limit the effectiveness of standard vector control and case management approaches [10]. Individuals who spend most of their nights sleeping outdoors in the forest, forest fringe or on farms, are at the greatest risk of malaria transmission, including those engaged in plantation labour, farming, logging, hunting, and construction [12-14].

At a regional level, malaria strategies in the GMS focus on strengthening country-led interventions, with an emphasis on malaria case and vector surveillance, monitoring and evaluation, and improving access to diagnosis, treatment and other essential services, particularly for high risk populations [11]. In recent years, GMS countries have intensified and refined malaria elimination approaches with a shift from country-based interventions to foci-based approaches [10]. Cambodia, Lao PDR and Myanmar have begun implementing national intensification plans and more aggressive approaches in hotspots [10]. It is expected that in 2021 all GMS countries will adopt similar intensive elimination efforts [10]. In Thailand, malaria elimination activities being increasingly mainstreamed and Vietnam has focussed on strengthening its surveillance system in hotspots [10].

6.4. Enhancing surveillance in the transition from malaria control to elimination

Once the number of malaria cases has been reduced to low levels in a particular area or country, the priorities and activities of a malaria programmes may need to be adjusted in order to achieve elimination [3]. In addition to continuing interventions to ensure universal access to malaria prevention, diagnosis and treatment (Pillar 1 of WHO's Global Technical Strategy), programmes should "enhance surveillance to ensure that every infection is detected and implement targeted measures for attacking both parasites and vectors in order to interrupt local transmission, eliminate all parasites from humans, and manage the risk of re-establishment through imported malaria" [3]. Strengthening malaria surveillance is fundamental to malaria programme planning and implementation and is crucial for accelerating progress towards elimination [3]. Surveillance encompasses the whole process of recording, reporting, analysing and interpretating data, and using the information for decision making related to prevention, case detection and treatment of malaria cases, antimalarial drug efficacy and drug resistance, and vector control. In areas targeted for elimination, such as much of the GMS, malaria-specific reporting systems are increasingly needed to meet the demands of targeting and monitoring interventions to particular high-risk groups and foci. As malaria caseloads decrease, it is necessary to implement timely and effective case-based surveillance and response systems to ensure every malaria case is investigated in order to understand risk factors and eliminate foci

of transmission [3, 4]. Thus, information systems must become increasingly "granular" allowing the identification, tracking and classification of all malaria cases and implementation of appropriate response activities [2].

6.5. Reactive surveillance and response activities in elimination settings

Once a parasitologically confirmed malaria case is detected by a health worker or a volunteer, timely case notification, case investigation and focus investigation must be conducted for reliable determination of the source of infection and classification of cases and foci to inform appropriate response. These activities are interconnected and are collective referred to as case investigation foci investigation and response" (CIFIR) or herein as "Reactive surveillance and response activities" [10]. The World Health Organization (WHO) recommends that national programs define a suitable schedule for case investigation, case detection and focus investigation and conduct regular monitoring and evaluation of their surveillance systems [3]. Furthermore, in elimination settings, the WHO recommends that countries classify malaria as a notifiable disease by national legal requirements and every case of malaria is to be notified to the responsible health authorities of their area.

6.5.1. Case and focus investigations

Correct epidemiological classification of malaria cases becomes more important in malaria elimination settings when there are very few cases, because it is the basis for classifying foci and for planning appropriate surveillance and other control measures [15]. The WHO recommends that for each parasitologically-confirmed malaria case, a case investigation form is to be completed to provide characterization of the case including the demographics of the patient, history of current illness, diagnostic test results, treatment received, and a complete travel history to provide information on how and where the infection might have been acquired and the possibility of onward transmission. Afterwards, the case should be classified as imported, introduced, indigenous, relapsing, recrudescent or induced, as per the case definitions described in

Table *1* [2].

Table 1. Classification of malaria cases according to WHO [2]

Type of case	Definition
Imported case	Malaria case or infection in which the infection was acquired outside the area in which it is diagnosed
Locally acquired case	A case acquired locally by mosquito-borne transmission<br>Note: Locally acquired cases can be indigenous, introduced, relapsing or recrudescent; the term "autochthonous" is not commonly used.
Indigenous case	A case contracted locally with no evidence of importation and no direct link to transmission from an imported case
Introduced case	A case contracted locally, with strong epidemiological evidence linking it directly to a known imported case (first-generation local transmission)
Relapsing case	Malaria case attributed to activation of hypnozoites of *P. vivax* or *P. ovale* acquired previously<br>Note: The latency of a relapsing case can be > 6 – 12 months. The occurrence of relapsing cases is not an indication of operational failure, but their existence should lead to evaluation of the possibility of ongoing transmission.
Induced case	A case the origin of which can be traced to a blood transfusion or other form of parenteral inoculation of the parasite but not to transmission by a natural mosquito borne inoculation<br>Note: In controlled human malaria infections in malaria research, the parasite infection (challenge) may originate from inoculated sporozoites, blood or infected mosquitoes.

The correct epidemiological classification of malaria cases is the basis for classifying foci and selecting appropriate response measures, where a "focus" is a defined as *"a circumscribed area situated in a currently or formerly malarious area that contains the epidemiological and ecological factors necessary for malaria transmission"* [10]. For every malaria case, a focus investigation is to be conducted and the focus is classified/updated as one of three categories: active, residual non-active, or clear focus (**Table 2**) [2]. A focus investigation involves identifying the main features of a location, populations at risk, the rates of infection or disease, the distribution of vectors responsible for malaria transmission and the underlying conditions that support it [15]. Focus investigations may also involve demographic, epidemiological,

entomological and environmental surveillance and monitoring of intervention coverage and quality [15].

Case and focus investigating teams may include:

- Health workers at health facilities or intermediate-level malaria focal points
- Skilled laboratory technicians, if microscopy is the main diagnostic tool or a health worker with good training in RDTs when these tests are used for surveillance
- Epidemiologists
- Entomological staff from intermediate or central levels
- Local health facility personnel and village health volunteers [15]

Table 2. Classification of malaria focus and operational criteria according to WHO [2]

Type of focus	Definition	Operational criteria
Active	A focus with ongoing transmission	Indigenous case(s) have been detected within the current calendar year
Residual non-active	Transmission interrupted recently (1-3 years ago)	The last indigenous case(s) was detected in the previous calendar year or up to 3 years earlier
Cleared	A focus with no indigenous transmission for more than 3 years	There has been no indigenous case for more than 3 years, and only imported or/and relapsing or/and recrudescent or/and induced cases may occur during the current calendar year.

6.5.2. Focus based surveillance and response activities

Routine activities that underpin the elimination of malaria in a focus include the following:

- Implementation of RACD when cases are few (for example no more than 3 cases per week per investigation team)
- Implementation of proactive case detection (PACD) among high-risk groups or during high-risk periods (high transmission season) if cases are still too many to implement reactive case detection (RACD)
- Focus investigation and response micro-planning

- Continuous community mobilization to participate in elimination activities and communication to raise awareness
- Follow up of cases once a case investigation and/or a RACD approach is in place to ensure compliance with treatment
- Regular entomological surveillance through representative sentinel sites, supplemented with spot checks during the focus investigation as necessary
- Annual monitoring and evaluation activities to track trends in malaria, ensure optimization of interventions, including surveillance systems, and to reclassify foci as necessary.

In addition, specific focus response activities may be triggered depending on the classification of the focus as active, residual non-active or cleared (**Table 3**). Some focus response activities such as providing treatment to infected individuals, supplementary vector control and increasing community awareness can be done during house-to-house visits during RACD [16]. Reactive case detection is triggered by the identification and notification of an index case and may be implementing within the household of an index case, within a radius around the household, or within the whole focus. Household members, neighbours, and other contacts of passively detected index cases are screened for infection and treated [17-19]. Depending on the type of transmission focus, RACD may be combined with other reactive surveillance and response activities, such as vector control and community education and participation.

Table 3. Different focus response activities based on the types of malaria focus and case [16]

Active focus		Residual non-active focus		Clear focus	
**Local case**	**Imported case**	**Local case**	**Imported case**	**Local case**	**Imported case**
Case detection in index household or radius or whole focus (if unusual parasite). Cases treated, investigation forms completed for all and classified.<br><br>Raise awareness about possible causes of transmission and advise on prevention and treatment. Provide additional vector control if needed.If no recent entomological data, do spot checks. Pay close attention to new developments that pose risks of transmission<br><br>Complete focus investigation form (if no focus investigation in last 4 weeks).<br><br>Update focus register and maps.	Case detection in index household or radius or whole focus (if unusual parasite). Cases treated, investigation forms completed for all and classified. Co-travellers tracked, tested, treated and investigation forms completed.<br><br>Raise awareness about possible causes of transmission and advise on prevention and treatment.<br><br>Provide additional vector control if needed. If no recent entomological data, do spot checks.<br><br>Pay close attention to new developments that pose risks of transmission.<br><br>Complete focus investigation form (if no focus investigation in last 4 weeks); updated focus register and maps	Case detection in whole focus. Cases treated, investigation forms completed for all and classified. Any introduced case must be clearly linked with imported case.<br><br>Raise awareness about possible causes of transmission and advise on prevention and treatment.<br><br>Provide additional vector control if needed. For local cases, assess reasons for secondary or primary transmission.<br><br>If no recent or relevant entomological data, do spot checks.<br><br>Pay close attention to new developments that pose risks of transmission (receptivity and vulnerability).	Case detection in index household or radius. Cases treated, investigation forms completed for all and classified. Co-travellers tracked, tested, treated and investigation forms completed.<br><br>Raise awareness about possible causes of transmission and advise on prevention and treatment.<br><br>Provide additional vector control if needed.<br><br>For local cases, assess reasons for secondary or primary transmission.<br><br>If no recent or relevant entomological data, do spot checks.<br><br>Pay close attention to new developments that pose risks of transmission (receptivity and vulnerability).	Case detection in whole focus. Cases treated, investigation forms completed for all and classified. Any introduced case must be clearly linked with imported case.<br><br>Provide additional vector control if needed; raise awareness.<br><br>Undertake entomological surveillance to establish reasons for local transmission.<br><br>Pay close attention to new developments that pose risks for transmission (receptivity and vulnerability)<br><br>Complete focus investigation form. Update focus register and maps. Reclassify focus immediately as active if local case is indigenous. Develop appropriate response plan to interrupt transmission.	Case detection in index household or radius. Cases treated, investigation forms completed for all and classified. Co-travellers tracked, tested, treated and investigation forms completed.<br><br>Raise awareness about possible causes of transmission and advise on prevention and treatment.<br><br>Provide additional vector control if needed.

6.6. Reactive surveillance and response strategies in the Greater Mekong Subregion

6.6.1. China's 1-3-7 strategy

WHO recommends national malaria programmes develop standard operating procedures defining a suitable schedule for the timing of reactive surveillance and response activities following the detection of a positive malaria case in elimination settings [10]. China commenced a malaria elimination program in 2010, aiming to achieve country-wide elimination by 2020. China formulated its elimination goal based on a four-catalogue classification of malaria transmission risk and incidence at county level. Since 2010, China has classified its 2858 mainland counties according to "types" based on the malaria burden. Each year, the number of "Type 1" and "Type 2" counties – those with malaria transmission in the last 3 years – has steadily declined to zero. From just under 5000 indigenous cases in 2010, China reported zero indigenous cases for the third consecutive year in 2019 and applied for WHO certification of malaria elimination [1]. China's elimination success has been partly attributed to their development and use of the **"1-3-7 strategy"** which entails case notification within 1 day, case investigation within 3 days, and focus investigation and appropriate public health response within 7 days [11]. Whilst China's health infrastructure has enabled successful implementation of 1-3-7 it is unclear how this strategy may be translated or adapted to different transmission settings.

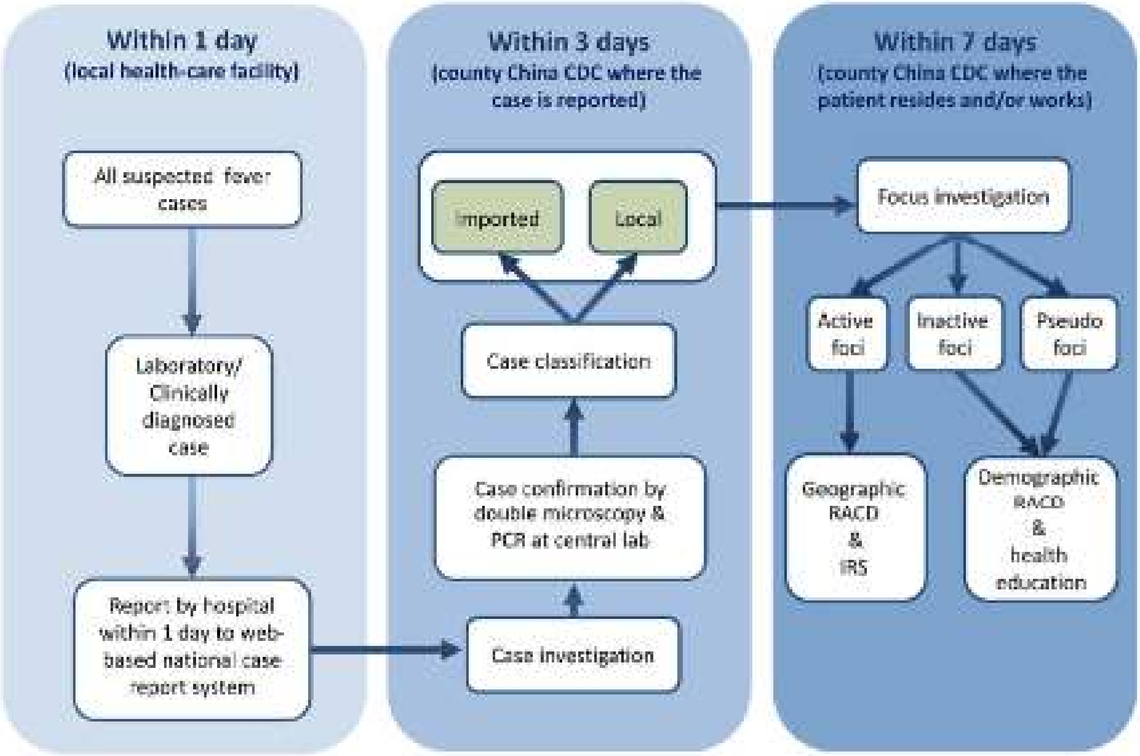

Figure 3. The chain of events conducted within the 1-3-7 day time windows (adapted from Cao J et al. Communicating and Monitoring Surveillance and Response Activities for Malaria Elimination: China's "1-3-7" Strategy. PLoS Medicine. 2014 May; 11(5): e1001642.https://www.ncbi.nlm.nih.gov/pmc/articles/PMC4019513/). IRS, indoor residual spraying.

6.7. Rationale for a region-wide study of reactive surveillance and response strategies in the GMS

Although the 1-3-7 strategy has now been deployed by the Asia Pacific Malaria Elimination Network (APMEN) and National Malaria Control Programmes (NMCP) in GMS countries, adherence to this schedule and case and foci investigation and response activities are thought to vary widely [6][20]. A survey of thirteen Asia Pacific Malaria Elimination Network (APMEN) member countries found that countries employ a wide variety of case investigation and active case detection activities [21]. Studies in specific areas of Myanmar and Cambodia have investigated the feasibility, knowledge of and adherence to the 1-3-7 strategy (including an evaluation of a simplified 1-3-7 model in Cambodia) [7-9]. Limited analyses of the yield of reactive case detection in the GMS has indicated low yield suggesting refinement of approaches is necessary [22]. To-date there have been no comprehensive, region-wide systematic

evaluations of malaria case investigation, reactive surveillance and response approaches in the GMS in terms of their effectiveness, acceptability and feasibility of implementation. Notably, there has been no formal assessment of health systems' receptivity to the 1-3-7 approach or alternatives. Evidence for the effectiveness, acceptability and feasibility of the 1-3-7 approach and other reactive surveillance and response strategies is needed to enable NMCPs in the GMS to adopt the most effective and appropriate strategy. Optimisation of the 1-3-7 surveillance and response approach from the China context to that of GMS countries' health infrastructure may be warranted to advance progress towards regional malaria elimination goals.

The aim of this mixed methods study is to investigate how GMS countries are currently applying reactive surveillance and response strategies, also known as case investigation, foci investigation and response (CIFIR) strategies, including but not exclusive to, the 1-3-7 strategy, in malaria elimination programs. The study will examine how these strategies may be optimised and synthesised improving their quality, effectiveness, and coverage in the context of existing national health systems for achieving regional malaria elimination targets.

This first stage will consist of a formative assessment of current to malaria reactive surveillance and response strategies in five GMS countries. Findings of this stage 1 study will be a basis for adapting and creating a realistic and feasible reactive surveillance and response strategy for malaria elimination in the region.

7. Aims and Objectives

7.1. Aim of study

To conduct a formative assessment of current malaria reactive surveillance and response strategies in the malaria elimination programs of GMS countries (Cambodia, Lao PDR, Myanmar, Thailand and Vietnam) and Yunnan Province, China, and identify how these strategies may be optimised and synthesised to improve their quality, effectiveness, and coverage in the context of existing national health systems for achieving regional malaria elimination targets.

7.2. Research Questions

1. What malaria reactive surveillance and response strategies are currently being implemented in the GMS and how do they differ between localities? Are these strategies species-specific and do they address the specific challenges of *P. vivax* infections in the GMS?

2. What are the knowledge, attitudes and practices of malaria program stakeholders regarding malaria reactive surveillance and response strategies in GMS countries?

3. What is the feasibility of implementing time-bound reactive surveillance and response strategies in GMS countries?

4. What are the barriers and enablers to successful implementation of reactive surveillance and response strategies GMS countries?

5. Are program stakeholders currently adhering to reactive surveillance and response strategies?

6. What is the timeliness and completeness of reactive surveillance and response activities in GMS countries?

7. What are the estimated positive case yields of reactive case detection (RACD) in GMS countries?

8. What is the acceptability of current malaria reactive surveillance and response strategies to malaria program stakeholders and beneficiaries in the GMS countries?

9. How can malaria reactive surveillance and response activities be improved to better target forest-goers and mobile and migrant populations?

10. How can participation of community health workers be improved in reactive surveillance and response activities?

11. Are malaria reactive surveillance and response strategies effective in progressing national malaria elimination agendas?

12. Which adaptations of the current malaria reactive surveillance and response strategies can be made to overcome existing barriers and improve their effectiveness in malaria elimination in the GMS countries?

7.3. Research Objectives

1. To describe the malaria reactive surveillance and response strategies currently being implemented in five GMS countries (Cambodia, Lao PDR, Myanmar, Thailand and Vietnam) and Yunnan Province, China

2. To investigate the knowledge, attitudes and practices of GMS malaria program stakeholders current malaria reactive surveillance and response strategies, including the adherence of the program stakeholders to the strategies

3. To identify barriers and enablers to the successful implementation of reactive surveillance and response strategies in the GMS

4. To determine the effectiveness of the current malaria reactive surveillance strategies in the five GMS countries, including the positive case yield of RACD

5. To investigate the timeliness and completeness of reactive surveillance and response activities in GMS countries

6. To explore the acceptability of reactive surveillance and response strategies to malaria program stakeholders and beneficiaries and the feasibility of successful implementation of time-bound reactive surveillance and response strategies in GMS countries

7. To explore how the current malaria reactive surveillance and response strategies can be adapted to overcome existing barriers and improve their effectiveness in malaria elimination in the five GMS countries, including those measures regarding the participation of CHWs in the strategies and optimization of the strategies for the MMPs

7.4. Study Outcomes

1. A description of malaria reactive surveillance and response strategies currently being implemented in Cambodia, Lao PDR, Myanmar, Thailand and Vietnam and Yunnan Province, China

2. Assessment of knowledge, attitudes and practice of malaria program stakeholders in the GMS countries regarding different activities of their current malaria reactive surveillance and response strategies

3. Estimated proportion of malaria cases that result in timely and complete case notification, case and focus investigations, and response activities according to the current national time-bound reactive surveillance and response strategies in GMS countries

4. Estimated positive case yield of RACD in the GMS countries in areas where it is practiced

5. Views and perspectives different malaria program stakeholders and beneficiaries in GMS countries on the acceptability of the current malaria reactive surveillance and response strategies

6. Identified policy, strategic, and operational barriers and enablers to successful implementation of the malaria reactive surveillance and response strategies in GMS countries

7. A matrix of opinions and suggestions on how the current malaria reactive surveillance and response strategies in the GMS countries can be optimized so that the existing barriers are overcome, effectiveness in malaria elimination is improved, and the strategies fit well into current elimination settings in the region

8. Research Design and Methods

8.1. Mixed-methods study design

A region-wide formative assessment of malaria reactive surveillance and response strategies currently being used in GMS will be performed, with a focus on target schedules of case notification, case investigation, foci investigation, and response (CIFIR) activities, and persons/departments responsible for these activities.

This formative assessment will include four components:

Part A: Systematic review of published and grey literature on malaria reactive surveillance and response strategies

Part B: Questionnaire survey of malaria program field staff, field supervisors, and frontline service providers

Part C: Qualitative assessment (interviews and focus group discussions) with malaria program stakeholders (current and past), key experts, and beneficiaries, and surveillance focal persons

Part D: Secondary data analysis of malaria case notification, foci investigation, reactive surveillance and response data from GMS countries

Part A: Systematic review

A systematic review of the published and grey literature on malaria reactive surveillance and response strategies will be conducted to establish evidence of different malaria reactive surveillance and response strategies worldwide, and how they are currently being implemented and how effective they are in the current malaria elimination settings of different countries. In addition to the main review of global literature, an additional focused examination of standard operating procedures, relevant forms (e.g., case investigation forms, focus investigation forms) and other policy and procedural documents relevant to malaria reactive surveillance and response activities of the GMS countries will be conducted. Findings of Part A will also help formulate the quantitative and qualitative data collection tools to be used in Parts B and C.

Part B: Questionnaire survey of malaria program stakeholders

Quantitative surveys of malaria program stakeholders from ministries of health, national malaria control programs and malaria implementing partners, and frontline malaria service providers in the five GMS countries will be conducted using survey questionnaires. Personnel responsible for managing, coordinating and supervising the field level malaria reactive surveillance and response activities, and frontline malaria service providers in the field will be included in the study. Two different survey questionnaires will be used to collect information about the knowledge, attitudes and practices of malaria program stakeholders regarding current malaria reactive surveillance and response strategies, and acceptability and feasibility of the current malaria reactive surveillance and response strategies currently implemented in their countries.

Part C: Qualitative assessment (interviews and focus group discussions) of malaria program stakeholders, frontline malaria service providers and beneficiaries

Semi-structured interviews and focus group discussions with malaria program stakeholders from ministries of health, national malaria control programs and malaria implementing

partners, frontline malaria service providers and beneficiaries of malaria programs, including mobile and migrant groups at heightened risk of malaria, from the GMS countries will be conducted. The qualitative assessment will further explore perceptions and practice of the stakeholders regarding the current malaria reactive surveillance and response strategies, acceptability of the strategies, and feasibility of successful implementation of the strategies in GMS countries. It will also explore how the current malaria reactive surveillance and response strategies can be optimized so that they fit well into current elimination settings of different GMS countries. Implementation strategies for the questionnaire and interviews will be flexible and adaptive and may vary according to country and respondent due to ongoing constraints associated with the COVID-19 pandemic and competing work priorities.

Part D: Secondary data analysis

Secondary data analysis of malaria case-based reporting data and reactive surveillance and response data from GMS countries in 2019 and 2020 will be conducted to investigate adherence to and effectiveness of different malaria reactive surveillance and response strategies in each country where this data is available.

The coverage of different components of the study for different research questions is described in **Table 4**. A summary of study populations/data source, study methods and targeted number of participants is described in **Table 5**.

Table 4. Alignment between components of study and research questions

Research Question	Part A Systematic review	Part B Questionnaire Survey	Part C Interview/ FGD	Part D Secondary data analysis
1. What are the malaria reactive surveillance and response strategies currently being implemented in GMS countries and how do they differ between the localities? Are these malaria reactive surveillance and response strategies species-specific and do they address the specific challenges of *P. vivax* infections in the GMS?	*	*	*	
2. What is the knowledge, attitudes and practice of malaria program stakeholders regarding malaria		*	*	*

reactive surveillance and response strategies in GMS countries?				
3. What is the feasibility of implementing time-bound reactive surveillance and response strategies in GMS countries?	*	*	*	
4. What are the barriers and enablers to successful implementation of reactive surveillance and response strategies in GMS countries?	*	*	*	
5. Are program stakeholders currently adhering to reactive surveillance and response strategies?		*	*	*
6. What is the timeliness and completeness of reactive surveillance and response activities in GMS countries?		*	*	*
13. What are the estimated positive case yields of reactive case detection (RACD) in GMS countries?				*
14. What is the acceptability of current malaria reactive surveillance and response strategies to malaria program stakeholders and beneficiaries in GMS countries?	*	*	*	
15. How can malaria reactive surveillance and response activities be improved to better target forest-goers and mobile and migrant populations?		*	*	
16. How can participation of community health workers be improved in reactive surveillance and response activities?		*	*	
17. Are malaria reactive surveillance and response strategies effective in		*	*	

progressing national malaria elimination agendas?				
18. Which adaptations of the current malaria reactive surveillance and response strategies can be made to overcome existing barriers and improve their effectiveness in malaria elimination in GMS countries?	*	*	*	

Table 5. A summary of study populations/data sources, methods and targeted number of participants

Group/Data source	Methods	No of participants	
		Per country	Expected total
Published and grey literature	Systematic review	NA	NA
Malaria program policy makers, managers (current and past), key experts, and beneficiaries and surveillance focal persons.	Semi-structured interviews	5	25
Field staff and field supervisors	Questionnaire 1	40	200
	FGD	4 FGDs	20 FGDs
Frontline malaria service providers	Questionnaire 2	40	200
	FGD	4 FGDs	20 FGDs
MMPs	FGD	3 FGDs	15 FGDs
Malaria case-based reporting data and reactive surveillance and response data	Secondary data analysis	NA	NA

Note: *The number of participants for each qualitative assessment methods are just indicative. The number of participants in each FGD session can range from 6 to 8. Whilst attempts will be made to conduct research activities in each of six GMS countries, research targeting frontline service providers and MMPs will likely be restricted to Cambodia, Lao PDR, Myanmar and Vietnam where HPA operates and Thailand where SMRU operates.*

8.2. Part A: Systematic Review of published and grey literature on malaria reactive surveillance and response strategies

8.2.1. Administrative Information

Registration:

In accordance with the guidelines, our systematic review protocol was registered with the International Prospective Register of Systematic Reviews (PROSPERO) on xxxxxxx (registration number - xxxxxxxxxxxxxxxx).

Running title: Acceptability, feasibility and effectiveness of reactive surveillance and response strategies on reducing malaria cases: A systematic review

Start date: 15 January 2021

Anticipated completion date: 15 December 2021

Authors:

1) Julia Cutts
   a) Senior Research Officer, Burnet Institute, julia.cutts@burnet.edu.au
2) Win Han Oo
   a) Senior Program Manager (Health Security), Burnet Institute, winhan.oo@burnet.edu.au
3) Win Htike
   a) Program Manager, Burnet Institute Myanmar, win.htike@burnet.edu.au
4) Kaung Myat Thu
   a) Technical Specialist (Malaria), Burnet Institute Myanmar, kaungmyat.thu@burnet.edu.au
5) Paul Agius
   a) Statistician, Burnet Institute, paul.aguis@burnet.edu.au
   b) Department of Epidemiology and Preventive Medicine, Monash University, Melbourne, Australia
   c) Judith Lumley Centre, La Trobe University, Melbourne, Australia
6) Katherine O'Flaherty
   a) Research Officer, Burnet Institute, katherine.oflaherty@burnet.edu.au
7) May Chan Oo
   a) Project Manager (Malaria), Burnet Institute Myanmar, maychan.oo@burnet.edu.au
8) Galau Naw Hkawng
   a) Technical Specialist, Burnet Institute Myanmar, galaunaw.hkawng@burnet.edu.au
9) Freya Fowkes

a) Professor, Head of Malaria and Infectious Diseases Epidemiology Group, Burnet Institute, freya.fowkes@burnet.edu.au

Named contact: Julia Cutts

85 Commercial Road, Melbourne, VIC, 3004, Australia, julia.cutts@burnet.edu.au

Contributions:

Julia Cutts is the guarantor. Freya Fowkes provides technical supervision on all aspects. Win Han Oo is an independent data extractor as well as quality assessor of the included papers that will lead to synthesis and write up. Paul Agius will supervise all statistical analysis. Katherine O'Flaherty, Win Htike, Kaung Myat Thu, May Chan Oo and Galau Naw Hkawng will contribute in designing the review and, report and manuscript writing.

Support:

This systematic review is funded and supported by the United Nations Offices for Project Services (UNOPS). Burnet Institute has overall control of the data. The funder will have no input on the interpretation or publication of the study results.

8.2.2. Systematic review protocol

Aim:

To investigate the acceptability, feasibility and effectiveness of reactive surveillance and response strategies in reducing malaria cases and identify barriers and enablers of their successful implementation.

Review questions:

1) What malaria reactive surveillance and response strategies are being implemented in malaria endemic countries, particularly those approaching malaria elimination?
2) What is the effectiveness of different malaria reactive surveillance and response strategies in detecting malaria cases, reducing malaria burden, and advancing malaria elimination goals in malaria endemic countries, particularly those approaching malaria elimination?
3) What is the acceptability of malaria reactive surveillance and response strategies according to community members, local authorities and health stakeholders in malaria endemic countries?
4) What is the feasibility of implementing reactive surveillance and response strategies in malaria endemic countries?

5) What are the barriers and enablers to the successful implementation of reactive surveillance and response strategies?

Objectives:

1. To establish evidence for the effectiveness of malaria reactive surveillance and response strategies on detecting malaria cases, reducing malaria burden and advancing malaria elimination goals in malaria endemic countries

2. To establish evidence for acceptability and feasibility of different malaria reactive surveillance and response strategies for detecting malaria cases, reducing malaria burden and advancing malaria elimination goals in malaria endemic countries.

Methodology

A mixed methods systematic review will be conducted according to Preferred Reporting Items for Systematic Reviews and Meta-Analyses (PRISMA) guidelines [23]. Due to the anticipated heterogeneity in quantitative endpoints between studies, the systematic review will be predominantly descriptive. However, where possible pooled estimates for effectiveness of policy changes (e.g., effect on proportion of cases reported within a relevant defined timeframe) will be presented.

Search methods for identification of studies

For published studies, online electronic databases namely PubMed, Web of Science, Scopus, African Index Medicus, and LILACS (Latin American and Caribbean Health Sciences Literature) will be searched for quantitative and qualitative studies published in all years up to current date that examined malaria reactive surveillance and response strategies and their effectiveness, acceptability and feasibility without any restriction on the geographical locations of the studies implemented. Key words will include: malaria, vivax, falciparum, “case detection”, “case investigation”, “reactive surveillance”, “reactive case detection”, RACD, “focus investigation”, “foci investigation”, CIFIR, 1-3-7, “surveillance schedule”, “response schedule”. The reference lists of included studies will be searched for additional studies. Studies and relevant documents reported in languages other than English will be included and translated into English by translators, or where native speakers are unavailable, by using online translation applications.

Relevant white and grey literature relating to reactive surveillance and response strategies will be sourced websites such as the following:

- UN agencies (World Health Organization http://www.who.int/en/ , UNICEF http://www.unicef.org/ , UNOPS https://www.unops.org/english/Pages/Home.aspx);
- International Organizations (IO) and Non-Government Organizations (NGO) (Population Services International http://www.psi.org/ , Malaria Consortium http://www.malariaconsortium.org/
- International Organizations for Migration http://www.iom.int/ , Save the Children International https://www.savethechildren.net/ , Médecins Sans Frontières (MSF) International http://www.msf.org/ , Community Partners International http://cpintl.org/);
- Philanthropies and donor agencies (Bill and Malinda Gate Foundation http://www.gatesfoundation.org/ , United States Agency for International Development https://www.usaid.gov/ , UK Department for International Development https://www.gov.uk/government/organisations/department-for-international-development ,
- Australian Department of Foreign Affairs and Trade http://dfat.gov.au/pages/default.aspx , Asia Development Bank http://www.adb.org/ , Japan International Cooperation Agency http://www.jica.go.jp/english/).
- [OpenGrey (www.opengrey.eu; to date of search)]
- [Agency for Healthcare Research and Quality (AHRQ; www.ahrq.gov; to date of search)]
- [National Institute for Health and Clinical Excellence (NICE; www.nice.org.uk; to date of search).]
- EThOs

Criteria for considering studies for this review

Types of studies

Primary qualitative, quantitative, and mixed methods studies will be eligible for inclusion in this review.

Quantitative and mixed-methods studies

Population-based cross-sectional, case-control, cohort studies, randomized controlled trials (RCTs), mixed methods studies, program evaluations, and feasibility studies that present evidence addressing the review questions will be considered for inclusion in this review. Data collection methods may include surveys, questionnaires, audits, routine surveillance data, population-based malariometric surveys. Published systematic reviews with similar research aims and objectives will be identified and discussed where appropriate.

Qualitative studies

Studies that employ qualitative study designs such as ethnography, phenomenology, case studies, grounded theory studies and qualitative process evaluations will be considered for inclusion. Studies that use both qualitative methods for data collection (e.g. focus group discussions, interviews, observation, document analysis, open-ended survey questions) and qualitative methods for data analysis (e.g. thematic analysis, framework analysis, grounded theory) will be considered for inclusion. Qualitative studies from grey literature are also eligible.

Exclusions

The following types of publications will be excluded:

- Case reports or case series
- Letters, editorials, commentaries, reviews or other articles that do not present primary findings or data.
- Study protocols
- Conference abstracts

Study populations

Study populations will include those living in areas at risk of malaria (stable areas where Plasmodium Falciparum Annual Parasite Index (PfAPI) ≥0.1 per 1,000 pa & unstable areas where PfAPI > 0 and < 0.1 per 1,000 pa) as defined by the Malaria Atlas Project [24]. In addition, health care and key groups/individuals who have direct or indirect interactions with populations/individuals living in areas at risk of malaria will also be included where relevant. Examples include village heads, ministers of health, malaria program leads, malaria patients and other community members at risk of malaria, and public health officials.

Interventions of interest

This review will focus on malaria prevention, control and elimination activities undertaken in direct response to the detection of a positive malaria case, described herein as "reactive surveillance and response interventions". In some contexts hese activities are collectively referred to as "Case Investigation, Foci Investigation and Response, CIFIR" All reactive surveillance and response interventions will be included in the review, including overarching time-bound policies such as the 1-3-7 approach, as well as specific focus response activities like reactive surveillance, prevention, and control activities conducted in response to the identification of an initial index case or foci. For example, reactive case detection,

supplementary vector control, and community awareness activities.

Outcomes of interest

The primary criterion for study inclusion is examination of the acceptability, feasibility, or effectiveness of a malaria reactive surveillance and response strategy or strategies in a malaria endemic area. Outcomes relating to the effectiveness of reactive surveillance and response will include but will not be limited to, the number of positive malaria cases identified by microscopy, RDT, or PCR (or any combination thereof), the number of people screened for malaria symptoms, the number of malaria tests performed at either a local or national level. For example, number of cases identified by reactive case detection, foci investigations, and other response activities precipitated by the identification of an initial index case of malaria. This review will consider the perceptions and experiences of health care workers, policy makers, and other malaria program stakeholders, malaria patients and other community members with particular attention paid to findings relating to the acceptability, feasibility as well as barriers and enablers to the implementation of reactive surveillance and responses interventions in malaria endemic countries.

Selection of studies

One investigator will conduct initial literature searches and screen titles and abstracts for inclusion. Two investigators will independently screen the full text of identified studies against inclusion and exclusion criteria, with discrepancies resolved by discussion with a third investigator. Covidence will be used as a tool to coordinate the screening process.

Quality and Risk of bias assessment

For studies presenting quantitative findings, The Risk of Bias In Non-randomized Studies – of Interventions (ROBINS-I) assessment tool will be used to assess the risk of bias and classify studies in one of five levels: low risk, moderate risk, serious risk and critical risks of bias, and no information. If possible, we will attempt to report our level of confidence in synthesis recommendations relating to quantitative measures by adopting GRADE for effectiveness studies [25]. Quality and risk of bias in studies presenting qualitative data will be assessed using CERQual [26] and/or CASP tools https://casp-uk.net/casp-tools-checklists/.

Data extraction

Two investigators will independently undertake data extraction using a data extraction form (Appendix X). Discrepancies will be resolved following discussion with a third investigator. The data extraction form will cover the following information:

- Basic study/publication information (e.g. first author, journal/source, date of publication)
- Basic characteristics of study (e.g. study site, study design, participants, objectives)
- Details of reactive surveillance and response intervention(s) being studied
- Outcomes including acceptability, case detection, feasibility, barriers and enablers.
- Qualitative findings: themes, findings and quotations
- Quantitative findings: data source, outcome measures, summary of results.
- Conclusions

Data management, analysis, and synthesis

After data extraction, data analysis and synthesis will initially be conducted separately for qualitative and quantitative data and by intervention type.

Quantitative studies

Narrative descriptions of study characteristics, outcome measures, and key findings will be reported. For estimates of the effectiveness of reactive surveillance and response strategies on malaria testing and cases numbers, measures of association (odds ratio (OR), risk ratio (RRs), incidence rate ratio (IRR), or hazard ratios (HR)) and their 95% confidence intervals (CIs) will be extracted or derived using data reported in the publications. Where there are two or more studies that can be combined (e.g. same intervention and same outcome), a pooled estimate for each outcome will be calculated using a random-effects model. Where a study did not provide measures of association for the association between reactive surveillance and responses interventions and effectiveness in reducing malaria cases, or increasing screening or testing of potential malaria cases (or they could not be calculated using the information provided) the study results will be included in narrative terms only. Quantitative data will be summarized as a descriptive analysis (and inferential analysis whenever possible) using frequencies, distributions, ratios, means and proportions. Dichotomous outcomes will be presented using proportions with 95% confidence interval (CI). Continuous outcomes (number and/or percentage of people diagnosed with malaria by RDT, microscopy or PCR, number and/or

percentage of people who died from malaria, quantitative measures of barriers and enablers) will be analyzed using difference in means (with 95% CI) or medians.

Qualitative studies

Malaria reactive surveillance and response strategies currently worldwide will be described in narrative terms, using information extracted from published studies and white and grey literature. Qualitative data will be summarized as a comparative content analysis facilitated by matrix displays. Narrative reporting of qualitative data synthesis will be structured around SURE guideline categories (SURE Collaboration, 2011).

8.2.3. Overall outcomes of systematic review

1. A description of malaria reactive surveillance and response strategies
2. Evidence for the effectiveness of different malaria reactive surveillance and response strategies in reducing malaria burden and advancing malaria elimination goals worldwide
3. Evidence for the acceptability different malaria reactive surveillance and response strategies by program stakeholders and beneficiaries
4. Evidence for the feasibility of different malaria reactive surveillance and response strategies in their successful implementation worldwide
5. Identification of barriers and enablers to the successful implementation of reactive surveillance and response strategies worldwide.

8.3. Part B: Questionnaire survey of malaria program field staff, field supervisors, and frontline service providers

8.3.1. Study design

Cross-sectional quantitative survey questionnaires will be conducted with malaria program field staff, field supervisors and frontline malaria service providers from national malaria control programs and malaria implementing partners.

Two different survey questionnaires will be used to collect information about the knowledge, attitude and practices of malaria program stakeholders regarding the current malaria reactive surveillance and response strategies of their countries, and the acceptability and feasibility of these strategies.

Questionnaire 1 will be used for personnel responsible for field staff and field supervisors responsible for managing, coordinating and supervising field level malaria reactive surveillance and response activities. **Questionnaire 2** will be used for frontline malaria service providers in the field. See *Questionnaire 1* and *2* in **Appendix** .

8.3.2. Objectives

1. To determine knowledge, attitudes and practices of malaria program stakeholders in GMS countries regarding their current malaria reactive surveillance and response strategies, including the adherence of the field staff and frontline malaria service providers to the strategies and the timeliness and completeness of their reactive surveillance and response activities

2. To identify the facilitators and barriers in implementation and adherence of the current malaria reactive surveillance and response strategies in the GMS, and possible solutions to the identified barriers

3. To determine the acceptability of different malaria program stakeholders to the current malaria reactive surveillance and response strategies in the GMS

8.3.3. Outcomes

1. Level of knowledge of malaria program staff/volunteers in GMS countries regarding different activities of the malaria reactive surveillance and response strategies currently implemented in their countries

2. Attitudes of malaria program staff/volunteers regarding adherence to time-bound malaria reactive surveillance and response strategies currently implemented in their countries
3. The proportion of malaria program staff/volunteers in GMS countries who strictly follow different activities of the malaria reactive surveillance and response strategies currently implemented in their countries
4. Facilitators and barriers to the implementation and adherence of the current malaria reactive surveillance and response strategies in the GMS, and identification of possible solutions to the identified barriers
5. Level of acceptability of current malaria reactive surveillance and response strategies for malaria program staff/volunteers in GMS countries.

8.3.4. Study settings and study sites

In Cambodia, Lao PDR, Myanmar, Thailand and Vietnam, study areas will be identified in consultation with national malaria control programs and HPA with consideration of malaria epidemiology and current surveillance systems. In Cambodia, the study area would be located at Ratanakiri, and Stung Treng provinces. In Vietnam, the Gia Lai and Binh Phuoc areas will be included.

8.3.5. Study populations and sample size

Questionnaire 1

Approximately 40 personnel from Ministries of Health, National Malaria Control Programs (namely Cambodia National Malaria Center (CNM) in Cambodia, Centre for Malaria Parasitology and Entomology (CMPE) in Lao PDR, National Malaria Control Program (NMCP) in Myanmar, Bureau of Vector Borne Diseases (BVD) in Thailand, National Institute of Malariology, Parasitology and Entomology (NIMPE) in Vietnam) and their malaria program implementing partners in the GMS countries, who are responsible for managing, coordinating and supervising field level malaria reactive surveillance and response activities will be recruited from each country, to give a total of 200 participants. If possible, personnel from Yunnan Province, China will participate in the questionnaire. The samples size is estimated based on the availability of stakeholders in GMS countries, with consideration of likely logistical and accessibility challenges during the COVID-19 pandemic.

The participants for Questionnaire 1 may include but will not be limited to:

Cambodia:

- Operational District Malaria Supervisor (ODMS)
- Health Center Staff (HC Staff).

Lao PDR:

- Disease control unit leaders
- Vector unit staff in selected provincial and district health offices

Myanmar:

- Assistant Director/Team Leader (VBDC)
- Medical Officer (VBDC)
- Malaria Assistant
- Malaria Inspector
- Field Coordinator
- Health Assistant
- Public Health Supervisor (2)

Thailand:

- Bureau of Vector Borne Diseases (BVD) staff

Vietnam:

- Director and staff of provincial CDCs and District Health Centre and leaders
- Staff of CSOs working for malaria control in Vietnam

China:

- Yunnan Institute of Parasitic Diseases (YIPD) staff

Questionnaire 2

Approximately 40 frontline malaria service providers who are working under the supervision of either of the ministries of health, national malaria control programs and their malaria program implementing partners in GMS countries, who usually undertake malaria case detection and case management in the field and have to directly or indirectly take part in the field level malaria reactive surveillance and response activities will be recruited from each country, to give a total of 200 participants. The participants for Questionnaire 2 may include but not limited to:

Cambodia:

- Health Centre Staff

- Village Malaria Workers

Lao PDR:

- Health centre and primary healthcare facility staff (heads of facility and malaria unit leaders)
- Village Malaria Workers (VMWs)

Myanmar:

- Integrated Community Malaria Volunteer (ICMV)
- Public Health Supervisors (2)
- Midwives

Thailand:

- Village Health Volunteers

Vietnam:

- Commune Health Centre staff
- Village Health Workers and Volunteers

Exclusion criteria (for both Questionnaire 1 and 2)

Individuals with any one of the following criteria will be excluded from the study.

- Aged below 18 years
- Participant living in a village or a location not easily assessable by the investigator/data collector because of either remoteness of the location, difficult transportation, or ongoing armed conflict.

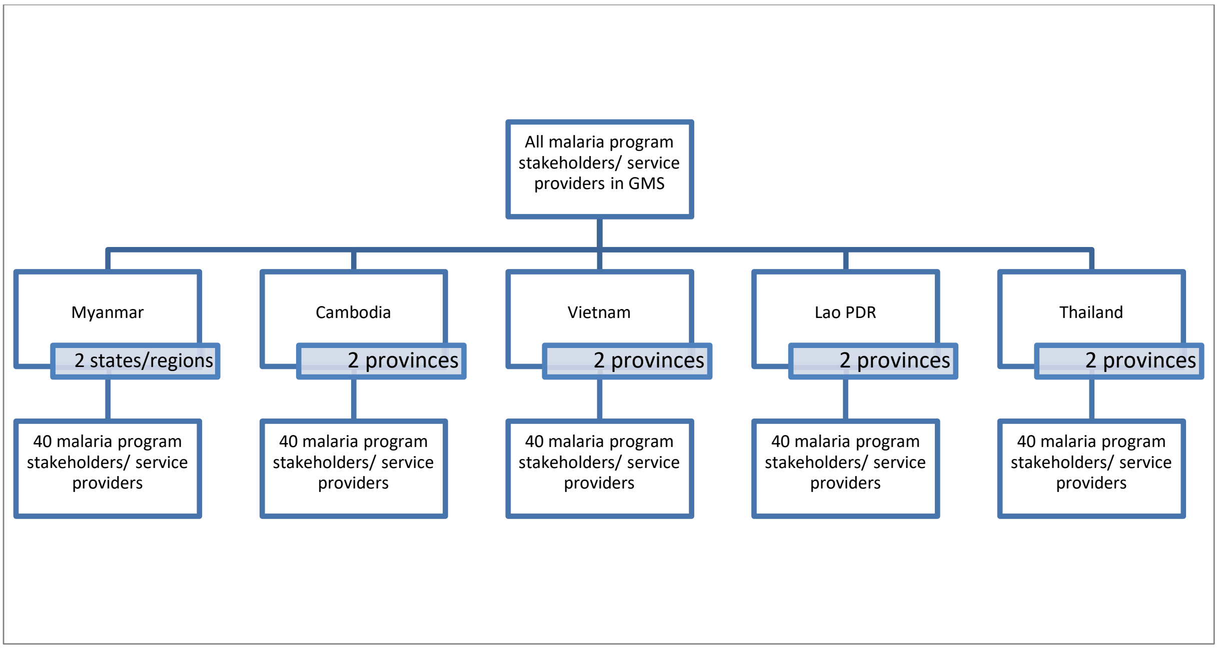

Figure 4. Sampling strategy for questionnaire surveys

8.3.6. Sampling Strategy and Participant recruitment

For both Questionnaire 1 and 2, approximately 40 malaria program stakeholders and service providers (respectively) from at least two provinces (or equivalent) in each GMS country will be purposively sampled. Personnel, staff and volunteers suitable for each questionnaire will be identified by the research team in consultation with the national malaria control programs and malaria program implementation partners of the respective countries, and/or in consultation with the RAI Regional Steering Committee (funders), and the sampling frame will also be developed based on this information. The consultation bodies will be contacted through email, mobile phone calling, video conferencing, or in person if possible. The target for sampling will be forty malaria program stakeholders and forty service providers from two provinces from each country (Figure 5).

The prospective participants will be approached by the research team in each country through the respective national malaria control programs and malaria program implementation partners of the country to request their participation in the study. For both Questionnaire 1 and 2, the prospective participants will be approached individually in their working place, or they may be individually or collectively invited to a meeting place most probably at the state/ regional/ provincial level malaria program offices for the recruitment procedure. For Questionnaire 2, the same procedure will apply but a meeting place nearest to their working place is preferred.

Investigators/trained data collectors will facilitate the informed consent taking procedure in person. If in-person meeting is not possible, Participant Information and Consent Form (PICF) will be sent via email and the informed consent taking procedure will be done through email, mobile phone calling or video conferencing.

Screening tools for identifying eligible states/regions/provinces and participants for the surveys are described in Error! Reference source not found..

8.3.7. Study procedures, data collection methods and tools

Once written informed consent has been obtained, participants will be surveyed by a surveyor using the semi-structured questionnaire which is framed in the REDCap which is a web-based application for building and managing online surveys and databases. The surveyor will be an investigator from the research team or a trained data collector familiar with the local context of the participant. The questionnaire will cover the sociodemographic characteristics of the participant, and knowledge, attitude and practice of the participant regarding the malaria reactive surveillance and response strategies currently implemented in their country. See details in **Appendix** . The survey questionnaires will be pilot tested with 1 – 2 malaria program stakeholders and 1 – 2 frontline malaria service providers from each country. Based on the pilot-testing results, the questionnaires will be reviewed and revised.

Surveys will be facilitated in-person by the surveyor (and one translator if necessary). The surveyor will record the participant's responses using the REDCap application on their Android OS running mobile devices (most probably a mobile phone or a mobile tablet). The semi-structured questionnaire will contain single and multiple responses questions with some pre-expected answers to choose to record, and open-ended questions which the surveyor will write down the responses in verbatim in their mobile application. The survey will be conducted in the local language of the participant, sometimes with the help of a translator if necessary, and they surveyor will translate the responses into English on site and record them in English into their mobile phones.

The surveyor may conduct the survey with his mobile application in online or offline mode. But once data verification and data cleaning (which will be done in the field on a daily basis) is done, the surveyor, with his mobile phone connected to an internet access and the REDCap application in the online mode, will submit the completed surveys to the Burnet Institute's

REDCap server (which will be done on a weekly basis). Then, the research team will access the completed surveys in the server through their computers.

Other considerations for study procedures

Implementation strategies for the survey will be flexible and adaptive and may vary according to country and respondent due to ongoing constraints associated with the COVID-19 pandemic and competing work priorities. The survey will be conducted in person whenever possible. However, if an in-person meeting is not possible, other most convenient possible methods will be used such as mobile phone calling, internet-based voice calling, or video-conferencing.

8.3.8. Data processing, management and analysis

Survey data in the Burnet Institute's REDCap server will be accessible only to the research team members from Burnet Institute, the data custodians being Professor Freya Fowkes and Dr Win Han Oo from Burnet Institute. The data in the server will be exported in Stata data format (".dta" and ".do") by the research team members from Burnet Institute. Then, the exported data will be imported into, processed and analysed using Stata version 15. The exported data will also be accessible only to Survey data in the Burnet Institute's REDCap server will be accessible only to the research team members from Burnet Institute.

8.4. Part C: Qualitative assessment (interviews and focus group discussions) with malaria program stakeholders, service providers and beneficiaries

8.4.1. Study design

Semi-structured interviews (in-depth interviews (IDI) and key-informant interviews (KII)) and focus group discussions (FGD) will be conducted with malaria program stakeholders from ministries of health, national malaria control programs and malaria implementing partners, frontline malaria service providers and beneficiaries of malaria programs from GMS countries namely Cambodia, Lao PDR, Myanmar, Thailand, Vietnam and China. To represent beneficiaries of malaria programs, mobile and migrant populations at higher risk of malaria will be approached in Cambodia, Lao PDR, Myanmar and Vietnam. Semi-structured interviews will be conducted with malaria program stakeholders who are responsible for designing or overseeing malaria reactive surveillance and response policy, strategies and activities. Focus group discussion will be conducted with malaria program stakeholders who are responsible for managing, coordinating and supervising field reactive surveillance and response activities, frontline malaria service providers, and mobile and migrant populations.

The qualitative assessment will further explore the perceptions and practice regarding the current malaria reactive surveillance and response strategies; acceptability to the strategies; and feasibility in successful implementation of the strategies in the five GMS countries. It will also explore how the current malaria reactive surveillance and response strategies can be optimized so that they fit well into current elimination settings of different GMS countries.

8.4.2. Objectives

1. To explore perceptions and practice of malaria program stakeholders in GMS countries regarding their current malaria reactive surveillance and response strategies, especially focusing on their adherence to the strategies

2. To explore the acceptability of current malaria reactive surveillance and response strategies to malaria program stakeholders and beneficiaries in GMS countries

3. To explore the feasibility of successful implementation of malaria reactive surveillance and response strategies in GMS countries

4. To explore how the current malaria reactive surveillance and response strategies can be adapted to overcome existing barriers and improve their effectiveness in malaria elimination in GMS countries, including participation of CHWs in the strategies and optimization of the strategies for the MMPs

8.4.3. Outcomes

1. Perceptions and practice of malaria program stakeholders in GMS countries regarding their current malaria reactive surveillance and response strategies, mainly focusing on the adherence to target schedules of different case notification, case investigation, focus investigations and response activities

2. Views and perspectives different malaria program stakeholders and beneficiaries in GMS countries on the acceptability of the current malaria reactive surveillance and response strategies

3. Identified policy, strategic, and operational barriers and enablers in successful implementation of the current malaria reactive surveillance and response strategies in GMS countries

4. A matrix of opinions and suggestions on how the current malaria reactive surveillance and response strategies in GMS countries can be optimized so that the existing barriers are overcome, effectiveness in malaria elimination is improved, and the strategies fit well into current elimination settings in the region

8.4.4. Study setting and study sites

Considerations of the study setting and study sites for Part C (the qualitative component) are the same as those for Part B.

8.4.5. Study population and sample size

Types of personnel, staff and MMPs suitable with the purposes of each qualitative study component will be identified by the research team in consultation with focal persons from the national malaria control programs (namely Cambodia National Malaria Center (CNM) in Cambodia, Centre for Malaria Parasitology and Entomology (CMPE) in Lao PDR, National Malaria Control Program (NMCP) in Myanmar, Bureau of Vector Borne Diseases (BVD) in Thailand, National Institute of Malariology, Parasitology and Entomology (NIMPE) in

Vietnam), Yunnan Institute of Parasitic Diseases (YIPD) in China, and their malaria program implementing partners from each of the five GMS countries. The number of participants for each qualitative component is as described in **Table 5** however the numbers are just indicative and data saturation will be the determinant factor for discontinuing participant recruitment.

Semi-structured interviews with higher and middle level malaria program stakeholders

Personnel from ministries of health, national malaria control programs and their malaria program implementing partners in the GMS countries, who are responsible for designing or overseeing malaria reactive surveillance and response policy, strategies and activities will be recruited for the interview.

The participants recruited for the interview may include but not limited to the following persons in each country of interest:

Regional organizations:

- WHO surveillance focal persons

Cambodia:

- Malaria Surveillance Working Group at CNM
- Provincial Health Department (PHD) and Operational District (OD) managers

Lao PDR:

- Director/Dy Director of CMPE
- Coordinator of the Administrative unit, CMPE
- Team Leader of the epidemiology unit, CMPE
- Team Leader of the technical management unit, CMPE
- Disease control unit leader in provincial and district health offices from the selected sites

Myanmar:

- Director/Program Manager (VBDC)
- Deputy Director (VBDC)
- Deputy Director (Disease Control)
- Assistant Director (VBDC)
- Program Manager (Malaria)

- Project Manager (Malaria)

Thailand:

- Director/Program Manager of National Malaria Control Programme

Vietnam:

- Director/Vice Director of Preventive Medicine Department – Ministry of Health
- Director/Vice Director of NIMPE – National Malaria Programme,
- Leaders and staff of Epidemiology Department,
- Vector Control/Entomology Department, NIMPE
- Director and staff of provincial CDCs and leaders
- Staff of CSOs working for malaria control in Vietnam.

China

- Director/Program Manager of Yunnan Institute of Parasitic Diseases (YIPD)

Approximately 5 interview sessions will be conducted in each country with a total of approximately 25 interviews.

Focus group discussion with lower-level malaria program stakeholders

Personnel from ministries of health, national malaria control programs and their malaria program implementing partners in the GMS countries (where possible), who are responsible for managing, coordinating and supervising the field level malaria reactive surveillance and response activities will be recruited for this FGD.

The participants recruited for this FGD may include but will not be limited to:

Cambodia:

- Operational District Malaria Supervisor (ODMS)
- Health Center Staff (HC Staff).

Lao PDR:

- Disease control unit leaders
- Vector unit staff in selected provincial and district health offices

Myanmar:

- Assistant Director/Team Leader (VBDC)
- Medical Officer (VBDC)

- Malaria Assistant
- Malaria Inspector
- Field Coordinator
- Health Assistant
- Public Health Supervisor (2)

Thailand:

- Bureau of Vector Borne Diseases (BVD) staff

Vietnam:

- Director and staff of provincial CDCs and District Health Centre and leaders
- Staff of CSOs working for malaria control in Vietnam

China:

- Yunnan Institute of Parasitic Diseases (YIPD) staff

Approximately 4 such FGD sessions will be conducted in each country with a total of approximately 20. The number of participants in each FGD session will range from 6 to 8.

Focus group discussion with frontline malaria service providers

Frontline malaria service providers who are working under the supervision of either of the ministries of health, national malaria control programs and their malaria program implementing partners in the GMS, who routinely perform malaria case detection and case management in the field and may directly or indirectly take part in the field level malaria reactive surveillance and response activities will be recruited for this FGD.

The participants recruited for this FGD may include but will not be limited to:

Cambodia:

- Health Centre Staff
- Village Malaria Workers

Lao PDR:

- Health centre and primary healthcare facility staff (heads of facility and malaria unit leaders)
- Village Malaria Workers (VMWs)

Myanmar:

- Integrated Community Malaria Volunteer (ICMV)
- Public Health Supervisors (2)
- Midwives

Thailand:

- Village health volunteers

Vietnam:

- Commune Health Centre staff
- Village Health Workers and Volunteers

Approximately 4 such FGD sessions will be conducted in each country with a total of approximately 20. The number of participants in each FGD session can range from 6 to 8.

Focus group discussion with mobile and migrant populations

Mobile and migrant populations (MMPs) who are at high risk of malaria infection and are usually receiving malaria prevention and treatment services from national malaria control programs and their malaria program implementing partners in the five GMS countries will be recruited for this FGD.

In this study, a ***migrant*** is defined as "*a person who takes up residence or remains in another place for an extended period of time (including seasonal migrants and forest dweller: Any person who regularly works in forests and stays overnight). A migrant moves from one location to another, regardless of duration or distance; experiences inequitable access to public health services resulting from the movement; and is vulnerable to becoming infected with malaria as a result of the movement*" and a ***mobile person*** is defined as *"any person who is constantly moving, such as truck drivers, seafarers, travelling salespersons, sex workers etc"* [27]. The MMPs recruited for this FGD may include but not limited to traditional slash-and-burn and paddy field farming communities visiting their forest farms, seasonal agricultural laborers, defense services, non-state combatants, internally displaced people and forest workers in the formal sectors (police, border guards, forest/wildlife protection services), forest workers in the informal sectors and formal and informal cross-border migrant workers. The MMP can be either internal or external.

Approximately 4 such FGD sessions will be conducted in each country with a total of approximately 20. The number of participants in each FGD session can range from 6 to 8.

Exclusion criteria

Individuals with any one of the following criteria will be excluded from the study.

- Aged below 18 years
- Participant living in a village or a location not easily assessable by the investigator/data collector because of either remoteness of the location, difficult transportation, or ongoing armed conflict.

8.4.6. Sampling strategy

A purposive sampling approach will be applied in recruiting the participants. Within each defined group of study population for each qualitative study component (for both interviews and FGDs), participant recruitment will encourage participation of a mix of different personnel, staff or MMPs representing different localities, different levels of responsibilities (such as national level, provincial level, township level, etc.), and different types of organizations (such as government, implementation partners, etc.) so that a rich and saturated data with diverse ideas and opinions can be obtained. For FGDs with MMPs, participation of different types of MMPs of all genders will also be encouraged.

Participant recruitment for the qualitative component has taken the following factors into consideration: limited number of malaria program stakeholders in each sub-group who are available to engage in the interview and the FGD, complexity of political landscape to interview government staff, prolong approval process and time constraint, and complexity of authorization to conduct FGDs and workshop in different locations, prolong approval process and time constraints. However, those factors will be weighed against the data saturation for continuing the participant recruitment.

8.4.7. Participant recruitment

Types of personnel, staff and volunteers suitable for each questionnaire will be identified by the research team in consultation with the national malaria control programs and malaria program implementation partners of the respective countries, and/or in consultation with the RAI Regional Steering Committee. The local administrative bodies may also be needed to consult for recruiting MMP participants for FGD. The consultation bodies will be contacted through email, mobile phone calling, video conferencing, or in person if possible.

The prospective participants will be approached by the research team in each country through the respective national malaria control programs and malaria program implementation partners

of the country to request their participation in the study. The prospective participants for interview will be approached individually in their working place, or they may be invited to a meeting place most probably at the state/ regional/ provincial level malaria program offices for the recruitment procedure. For prospective participants for FGDs, they will be invited to a meeting place most probably at the state/ regional/ provincial level malaria program offices for the recruitment procedure. For FGDs with MMPs, the prospective participants may also be approached at their current residing or working place if appropriate. Investigators/trained data collectors will facilitate the informed consent taking procedure in person. If in-person meeting is not possible, Participant Information and Consent Form (PICF) will be sent via email or physical mail and the informed consent taking procedure will be done through email, mobile phone calling or video conferencing.

8.4.8. Study procedures, data collection methods and tools

Semi-structured interviews

Once the written informed consent has been obtained, the participant will be interviewed by an interviewer who will mostly be a principal investigator or by a nominated in-country senior member of the research team at the discretion of the principal investigator. The interview will be conducted individually in-person in local language of the participant whenever possible, and the interview will be helped facilitated by a note taker and, if necessary, a translator. The interview will be conducted in a place with privacy so confidentiality can be maintained. The location of interviews will be determined separately for each interview and the location selected will depend on the potential sensitivities and risks relevant to each participant. Prior to commencing the interview, the interviewer will obtain non-identifying information relating to the job title, role/responsibility and relevant experience of the participant.

The interview will be aided by an interview topic guide. The topic guide contains questions covering the perceptions and practice of malaria program stakeholders regarding their current malaria reactive surveillance and response strategies, acceptability of the strategies, feasibility for successful implementation, and how the current strategies can be optimized for malaria elimination settings of the GMS countries. The interview topic guide can be seen in **Appendix D**. The wordings and sequence of the questions in the topic guide do not need to be exactly followed but can be modified depending on the exiting dynamic of the interview. The interviewer will facilitate the interview with open-ended questions to allow participants to reveal their opinions freely. The interview session will be audio recorded using voice recorders

and written notes will be taken with the informed consent of the participant. During the session, participant will be provided with refreshments. It is anticipated that each interview session will take approximately 60 minutes. After completion of each interview, the interviewer will complete notes of reflection on each interview within 24 hours of completion. To ensure the participants have opportunity to check what was said during the interview, the copy of transcript will be sent to each participant for their own record of the discussion through mail and/ or email where available.

Focus group discussions

Once the written informed consent has been obtained, the participants will be seated in circle with each two note-takers on the sides of the circle. The FGD will be facilitated by a facilitator who can be an in-country investigator of the research team or a trained facilitator/data collector familiar with the local context. The FGD will be conducted in local language of the participants whenever possible, with the help of a translator if necessary. The FGD will be conducted in a place with privacy so confidentiality can be maintained. The location of interviews will be determined separately for each FGD session and it will depend on the potential sensitivities and risks relevant to each participant. Prior to commencing the FGD, the facilitator will obtain non-identifying information relating to the age, sex, occupation and role/responsibility of the participants.

The FGD facilitator will be aided by an FGD topic guide. The topic guide contains questions covering the perceptions and practice of malaria program stakeholders regarding their current malaria reactive surveillance and response strategies, acceptability of the strategies, feasibility for successful implementation, and how the current strategies can be optimized for malaria elimination settings of the GMS countries. Topic guide for FGD with MMPs will focus mainly on the acceptability of the current reactive surveillance and response strategies/activities for them and how they can be optimized for them. Separate FGD topic guides for the three FGDs can be seen in **Appendix** D **D**. The wordings and sequence of the questions in the topic guides do not need to be exactly followed but can be modified depending on the exiting dynamic of the discussion session. The facilitator will facilitate the discussion with open-ended questions to allow participants to reveal their opinions freely and interact with each other to build responses. The FGD session will be audio recorded using voice recorders and written notes will be taken with the informed consent of the participant. During the session, participants will be provided with refreshments. It is anticipated that each FGD session will take approximately

60 to 90 minutes. After completion of each discussion session, the interviewer will complete notes of reflection on each session within 24 hours of completion. To ensure the participants have opportunity to check what was said during the focus group discussion, the copy of transcript will be sent to each participant for their own record of the discussion through mail and/ or email where available.

Other considerations for study procedures

Implementation strategies for the interviews and FGD will be flexible and adaptive and may vary according to country and respondent due to ongoing constraints associated with the COVID-19 pandemic and competing work priorities. The interview and FGD sessions will be conducted in person whenever possible. However, if in person meeting is not possible, other most convenient possible methods will be used; the interviews will be tried to conduct through mobile phone calling, internet-based voice calling, or video-conferencing; and FGDs will be tried to conduct through internet-based group calling (voice calling) or video-conferencing, with the help of a local health or administrative personnel if necessary.

Pilot testing of interview and FGD topic guides

Each interview and FGD topic guide will be pilot tested with similar participants before commencing actual data collection. Pilot testing will help fine-tune the topic guides that will lead to more reliable results and rich data. It will provide an opportunity to validate the wording of the tasks, understand the time necessary for the session and may supply additional data points for the research. It will also identify problems and barriers related to participant recruitment, including the informed consent procedure, and commence the engagement in the research as a qualitative researcher. Pilot testing will be conducted with at least participant for the interview topic guide and one pilot testing FGD session will be conducted for each FGD topic guide, if possible, once in each country. The participants in the pilot test will be selected to be as similar as possible to the intended participants in the actual study. The study procedure for the pilot test will be the same as the actual research described in this protocol. Where necessary, the data collection tools and procedures will be reviewed and revised following pilot testing.

8.4.9. Data processing and management

Each participant will be assigned a unique code linked to his or her data and no identifying information was stored with the collected data. Voice recorders used to take audio-recordings will be kept in a locked box in the field before the data will be transferred into a password-protected computer and will only be accessible to the investigators. Once the quality of electronic audio files has been confirmed, the original files in the recorders will be erased. The audio recordings will be transcribed verbatim and translated them into English by the in-country research team members from each country. Transcripts in English will be used for analysis. Written field notes will be stored in locked filing cabinets in the Burnet Institute office. No personal identifiers will be recorded on the written notes.

8.4.10. Qualitative data analysis

Deductive followed by inductive thematic analysis, including constant comparative analysis where appropriate, will be used. The process will include the steps of data immersion, coding, categorisation/sub-theme development and major theme development, guided by the collected data via an in-depth code guide. Emerging themes during the data collection will be captured and incorporated into the thematic framework in data analysis stage. One investigator will analyse all the data and another investigator randomly will extract 10% of the data and perform an independent analysis. Afterwards, both investigators will discuss the themes and subthemes and reach a consensus. The findings will be reported thematically. Key findings will be illustrated with direct quotations from the data. NVivo version 12 will assist the qualitative data analysis.

Member checking with all types of study participants will be done and reflexivity will be employed from the data collection stage up to the data analysis and reporting stages to improve the rigour of the study. Experiences of the data collection and analysis will also be considered during the write up.

8.5. Part D: Secondary data analysis

8.5.1. Study design

This component will involve cross-sectional analysis of routinely collected malaria case-based reporting data and reactive surveillance and response data during the 2019 and 2020 calendar

years in GMS countries namely Cambodia, Lao PDR, Myanmar, Thailand and Vietnam (excluding China) will be conducted to investigate adherence to and effectiveness of different malaria reactive surveillance and response strategies in each country.

8.5.2. Objectives

1. To determine the adherence (timeliness and completeness) of the national malaria control programs and malaria implementation partners to the malaria reactive surveillance and response strategies currently implemented in GMS countries

2. To determine the effectiveness of the current malaria reactive surveillance and response strategies in GMS countries, including the positive case yield of the strategies in the countries

3. To assess impact of the COVID-19 pandemic on the timeliness of case notification, case and focus investigations, and response activities

8.5.3. Outcomes

1. Number and percentage of malaria cases that result in timely and complete '**case notification, case and focus investigations, and response activities**' according to the current national reactive surveillance and response strategies in GMS countries

2. Number and percentage of positive cases yield from RACD in GMS countries

3. API and annual malaria positivity rate in villages implemented RACD in 2019 and 2020

Table 6: Malaria case reporting data metrics

Domain	Measurement metric	Reported as
Timeliness	Number and percentage of malaria positive test notifications completed within time frame specified by current reactive surveillance and response strategy in the country (e.g., 24 hours according to 1-3-7)	% (95% CI)
	Number and percentage of malaria positive test notifications for which case investigations were completed within time frame specified by current reactive surveillance and response strategy in the country (e.g., 3 days according to 1-3-7)	% (95% CI)
	Number and percentage of malaria positive test notifications for which focus investigations were completed within time	% (95% CI)

	frame specified by current reactive surveillance and response strategy in the country (e.g., 7 days according to 1-3-7)	
	Number and percentage of malaria positive test notifications for which all necessary response activities were completed within time frame specified by current reactive surveillance and response strategy in the country (e.g., 7 days according to 1-3-7)	% (95% CI)
Completeness	Number and percentage of positive test notifications for which all case investigation activities were completed	% (95% CI)
	Number and percentage of positive test notifications for which all focus investigation activities were completed	% (95% CI)
	Number and percentage of positive test notifications for which all necessary response activities were completed	% (95% CI)
Effectiveness	Number of positive cases yield from RACD activity in each GMS country	
	Percentage of positive cases derived from RACD among all malaria positive cases in each GMS country	% (95% CI)
	Village / commune API in villages implemented RACD in 2019 and 2020	
	Malaria positivity rate in villages implemented RACD in 2019 and 2020	% (95% CI)

8.5.4. Study setting, study sites and study population

Part D of the study will cover five GMS countries namely Cambodia, Lao PDR, Myanmar, Thailand and Vietnam (excluding China) and will try to collate nationally representative datasets for each GMS country in the study as much as possible. The period of the data will be the 2019 and 2020 calendar years.

8.5.5. Data retrieval, processing, management and analysis

National level national malaria control programs (namely Cambodia National Malaria Center (CNM) in Cambodia, Centre for Malaria Parasitology and Entomology (CMPE) in Lao PDR, National Malaria Control Program (NMCP) in Myanmar, Bureau of Vector Borne Diseases (BVD) in Thailand, National Institute of Malariology, Parasitology and Entomology (NIMPE) in Vietnam) will be approached to request national level datasets of routinely collected malaria case-based reporting data and reactive surveillance and response data. Malaria program implementation partners will also be approached where necessary. Data transfer agreements will also be obtained from the respective authoritative bodies (**Appendix**

F) before submission of the protocol to the respective institutional review boards in the study countries. The potential variables to be requested from the national malaria control programs is listed in **Appendix E**.

The study aim to collate malaria case-based reporting datasets and malaria reactive surveillance and response datasets from the national malaria control programs in each country for the 2019 and 2020 calendar years. Malaria reporting data will relate to malaria tests performed in health facilities and within communities under the supervision of the national malaria control program in each country. Secondary data will be made available from sources including, but not limited to, DHIS2, district malaria case registers, and any reporting frameworks of NMCPs. For the malaria reactive surveillance and response data, examination of the records, registers and reports of case notification, case investigation, reactive case detection, focus investigation and response activities may also be needed.

The data will be requested in Microsoft Excel format as appropriate. Data to be shared will be coded with a unique ID number by the relevant national malaria control program or implementation partner prior to sharing, but these linking documents will not be shared. Therefore, all data shared with research team members will be non-identifiable.

Data analysis will be performed with Stata version 15. The number and percentage of positive malaria test notifications for which reactive surveillance and response activities are completed and completed within the time frame specified by local policy will be calculated as outlined in **Table 5** and reported as percentages and 95% confidence intervals for each country. Where appropriate, proportions between countries or reporting systems will be compared using Chi-squared tests (Stata version 15.0, StataCorp, Texas, USA).

9. Results dissemination and future directions

A final technical report will be produced, and a final dissemination workshop will be held with the national malaria control programs and malaria implementation partners from each country to disseminate the findings of the study. Findings may also be presented at international and national conferences and published in peer-reviewed journals. The number and content of manuscripts will be dictated by study findings. All investigators will be given the opportunity to contribute to manuscripts, including opportunities for investigators from national malaria control programs of the GMS, to be first or joint first author.

Findings of this mixed methods study will be a basis for designing an optimized malaria reactive surveillance and response strategy for the GMS. There will be a three-month pilot implementation of the optimized strategy in order to identify barriers and enablers for its implementation and to inform refinements of proposed strategies. The penultimate model will then be presented to stakeholders in GMS for feedback, refinement, and finalisation.

10. Ethical considerations

10.1. Ethics review

This study protocol will be reviewed by the Alfred Hospital Ethics Committee in Melbourne, Australia, one of the Australian Government recognised Ethics Review Committees (ERCs) and in-country ERCs in Cambodia, Laos PDR, Myanmar, Thailand and Vietnam.

10.2. Informed consent

Two approaches of informed consent will be involved in this study. While written informed consent will be sought for all active participants (survey, interview and FGD) (for Part B and C), a waiver of consent will be sought for Part D – which involves the analysis of routinely collected, non-identifiable participant data collected throughout the 2020 calendar year – from both the Alfred Hospital Ethics Committee and the relevant in-country ERCs.

Written informed consent

Written informed consent will be obtained from all participants taking part in the survey, interviews and FGDs (Parts B and C). At time of recruitment, the investigator/data collector will thoroughly explain to all prospective participants the overarching scope and purposes of the study, the role of a participant in the study, the study procedures, and the risks and benefits of participating in the study which are described in the Participant Information and Consent Form (PICF) in details. The prospective participant will also be asked to read through the PICF. The researcher will confirm the participant's understanding of the information provided and adequately answer any questions. If the individual agrees to participate in the study, he/she will be asked to sign the PICF and the researcher will obtain written informed consent from the individual. The original signed PICFs for the participants will be stored in lockable document storage facility, such as locked folders or locked filing cabinets, and will be stored separately

from other study documentation. The PICF forms for the surveys, interview and FGDs in this study are attached in **Appendix G**.

The informed consent taking procedure will always be conducted in the local language of the prospective participant, with the help of a translator familiar with the local context if necessary. Care will be taken to prepare culturally appropriate and comprehensible explanations about the study with a particular emphasis on the participant's right to withdraw from participation at any time without a reason and without any consequences.

Waiver of consent

A waiver of consent will be sought for Part D (Secondary Data analysis) to cover the access and analysis of routinely collected, non-identifiable malaria program data on malaria cases and malaria reactive surveillance and response activities collected throughout the 2019 and 2020 calendar years. A waiver of consent is sought for the following reasons, consistent with factors listed in 2.3.10 of the National Statement on Ethical Conduct in Human Research:

a) This research poses no foreseeable risk to the participants. Participation in the secondary data analysis would be limited to the use of non-identifiable data relating to malaria tests, case investigation, foci investigations and other public health response activities conducted in response to the identification of a malaria case.

b) The benefits from the research (a greater understanding of the timeliness and quality of current malaria reactive surveillance and response strategies in the GMS countries) justify any risk of harm associated with not seeking consent noting that there is no foreseeable risk of harm associated with inclusion of one's malaria case record(s) in this study.

c) It is impracticable to obtain consent from all individuals whose malaria case records are included in the national datasets we intend to analyse because of the sheer volume of records (approximately 23,900 individual records). The datasets will include basic information on all malaria cases, malaria case notifications, case investigations, focus investigation and responses that occurred in the five GMS countries in the 2019 and 2020 calendar years. Therefore, it would be impracticable to obtain consent from each individual who contributed a malaria case record to this dataset.

d) There is no known or likely reason for thinking that participants would not have consented if they had been asked to participate in this study. Firstly, the same datasets are routinely analysed by national malaria control programs to investigate trends in the epidemiology of malaria in GMS countries. The analyses to be conducted by Burnet researchers, using the same datasets, come under the broader topic of malaria epidemiology, but are specifically focused on determining the proportion of malaria cases that are followed up with case investigations, foci investigations and other public health measures. Secondly, it is not foreseeable that analysis of these datasets by Burnet researchers will lead to any significant findings that would impact on an individual's health or welfare. Importantly, any health information determinable from the data (that is, malaria test results) has already been communicated to individual patients.

e) There will be sufficient protection of participant privacy because the records contained in the datasets will have already been collated and names and contact details removed by the relevant national malaria programmes or implementing partner prior to sharing the datasets with Burnet Institute. In other words, the individual to whom the malaria case record relates ("participant") will never be in a position where they are directly disclosing any information to the reserachers, as this information will have already been collected in 2019-2020.

f) There is an adequate plan to protect the confidentiality of data. The malaria case records will be non-identifiable to researchers because all names and contact details will have been removed by the relevant national malaria programmes or implementing partner prior to sharing the datasets with Burnet Institute. Datasets will be shared with Burnet Institute using a secure password protected sharing platform (most probably Google Drive). Subsequently, data will be stored on secure Burnet Institute servers, accessible only to authorised researchers at the Burnet Institute.

g) The results will not have significance for the participants' welfare because they relate to the effectiveness of existing malaria surveillance approaches, rather than being relevant to the health of specific individuals. Any health information contained in the datasets, namely the results of point of care malaria tests, will have already been communicated to the patient at the point of care.

h) There is no foreseeable possibility of commercial exploitation of derivatives of the data to deprive the participants of any financial benefits to which they would be entitled

i) The waiver is not prohibited by State, federal, or international law.

The list of data variables to be shared with the research team in this study is attached in **Appendix E**. The waivers of consent will be submitted to the Alfred Hospital Ethics Committee and in-country ERCs in the study countries as required.

10.3. Risks and Benefits

10.3.1. Risks

The risk of participating is minimal for all participants both individually and collectively in all individual components of the study. During the surveys, interviews and FGDs, the participants may feel some discomfort discussing some personal information and sensitive issues if there will be any. To minimise these risks, trained data collector/interviewer/investigator will conduct the surveys, interviews and FGDs in a private space where the participants cannot be overheard. The participants will always be allowed to refuse to answer any personal questions and questions in-any-way sensitive to them. Moreover, the materials containing the personal information and sensitive data will always be non-identifiable and the data will always be assessable to the research team members. Reporting of the findings about the sensitive issues will always takes cautions to make the data source non-identifiable. Researchers (interviewers) will always inform the interview and FGD participants about the use of digital voice recorders to audio-record interviews and FGDs before the start of each session and the use of these voice recorders will be done only with the consent of the participants.

10.3.2. Benefits

There will be no direct benefit to study participants for participating in the study, and this will be made clear in the PICF. However, the study participants will be provided with refreshments during the study procedures. The cost of travel and accommodation for study participants, if they will be any, will be reimbursed as considered appropriate by the principal investigator. Participants of survey and FGDs will be provided with a cash money not more than 5 USD to compensate their time spent for participation in this study. Participants of the semi-structured interviews will be provided with a token of appreciations worth not more than 5 USD for participation in this study. Apart from them, other forms of remuneration will not be provided to any participants in this study.

10.3.3. Confidentiality

All individual data entries will always be made non-identifiable. No personal information will be recorded on the data collection tools and materials. Personal information of the participants will be recorded in a separate document and numerical and/or alphabetical coding systems will be used to link the data with their personal identifications. The documents containing the personal information will be securely stored separately from the data and these documents will only be accessible only to the research team members. Signed PICF forms will also be securely stored separately from the survey forms, transcripts and audio-recordings. Moreover, all data-containing documents and material (for all Part B, C and D) will be securely in locked filing cabinet and they will be accessible to research team members. The audio records of the interviews and FGDs will be permanently deleted from the recorders when they are securely transferred to a password-protected computer. All forms of electronic data collected and processed in this study will always be securely stored in password-protected computers and password-protected cloud servers and they will be accessible only to the nominated research team members with the permission of the data custodians, Professor Freya Fowkes and Dr. Win Han Oo from Burnet Institute. All primary data collected through research will be destroyed within seven years.

11. Research Timeline

Activities	2021												2022											
	Jan	Feb	Mar	Apr	May	June	July	Aug	Sept	Oct	Nov	Dec	Jan	Feb	Mar	Apr	May	June	July	Aug	Sept	Oct	Nov	Dec
Protocol development																								
ERC submission and defend																								
Finalisation of protocol with ERC comments																								
Recruitment & training of data collectors																								
Initial engagement with GMS NMCPs (commenced since 2020)																								
Systematic review																								
Questionnaire survey																								
Semi-structured interviews																								
FGDs																								
Data processing & analysis																								
Report writing																								
Dissemination																								

Figure 5. Research timeline

12. Capability statement

The Burnet Institute is an Australian, not-for-profit, unaligned, and independent organization that links laboratory-based medical research with field-based public health programs aimed at creating a healthier world. The Burnet Institute is committed to improving the health of disadvantaged, poor or otherwise vulnerable communities and is focused on infectious diseases of global health significance, including malaria, HIV and AIDS, viral hepatitis, influenza, and tuberculosis. The Institute has over 380 staff located in Melbourne and internationally, including long-term offices in Myanmar, Papua New Guinea and Lao PDR. The Institute is also involved in programs in China, Africa, South Pacific, Indonesia, East Timor and Sri Lanka. The Burnet Institute works closely with local partners and government to ensure that information and services are culturally appropriate and comply with government policy and guidelines.

Burnet Institute Myanmar (BIMM) is Burnet Institute's largest country program. Since 2003, BIMM is committed to improving health care and services for communities in Myanmar. Staff members include public health professionals and social research scientists with organisational development, and monitoring and evaluation expertise, all of whom are passionate in their commitment to working together to create a healthier world. In 2005, BI-MM entered into a Memorandum of Understanding with the Ministry of Health and in 2012 was registered with the Ministry of Home Affairs which enables the program to work across 13 States and Regions in 94 townships. Current portfolio of BIMM shows 8 development and 2 research projects implementing a budget of AUD13m between 2014 and 2016.

BIMM is funded by a broad range of bilateral and multilateral agencies directly and through multi-donor trust funds; for example, the Australian Aid Program, UK Department for International Development, Global Fund for AIDS, Malaria and TB, 3 Millennium Development Goal Fund; European Union, Population Council and UNDP. BIMM is currently delivering projects across three sectorial priority areas of Maternal, Neonatal and Child Health, Infectious Diseases (HIV/AIDS, Malaria and TB) and School Health. Enabled by the socio-political reform agenda and strengthening relationship with the government, Burnet is now scaling up our research capabilities in Myanmar supported by our global expertise and reputation for high quality public health research and knowledge generation that links medical research with public health action.

BIMM prioritises a rights based, health systems strengthening approach to programming and deliver public health services directly and through local partners in collaboration with central, state and township level health authorities. Implementation approaches are diverse and are developed in response to identified need, they include (i) consortiums with International NGO's and other commercial bodies; (ii) partnership with Government agencies e.g. Department of Health, Department of Medical Research; (iii) Township Medical Officers as project counterparts; (iv) subcontracted to UN agencies; (v) consultancies with non-government organisations both local and international; and in (vi) partnership with international and/or domestic institutes with program alignment in public health e.g. Australian Broadcasting Corporation.

Our technical approaches include, community-based delivery of health care services and health seeking behaviour changes; township level health systems strengthening; increasing research and operational research, monitoring and evaluation and knowledge generation, dissemination, and publications. Our Country Representative and Senior management and technical staff participate and engage in Technical and Strategic Working Groups in Malaria, HIV, TB, Maternal and Child Health under the direction of the Myanmar Health Country Coordination Mechanism.

The malaria program at the Burnet Institute, Melbourne, is conducted across the Centre for International Health, Centre for Biomedical Research and the Centre for Population Health and has the following research focuses:

- Population studies in malaria-endemic countries, such as Thailand, Kenya and Papua New Guinea through collaborations with national research institutes based in those countries.
- Therapeutic studies of artemisinin efficacy - Burnet Institute is involved in the Tracking Resistance to Artemisinin Collaboration the largest international collaboration investigating the spread of parasite resistance to artemisinin-based therapies in sites in Asia and Africa.
- Epidemiological surveillance of malaria in high risk populations
- Implementing community-based management of malaria programs in the Asia-Pacific
- Immunity to malaria in humans, vaccines, new treatments, mechanisms of infection of red blood cells and clinical studies on malaria

- Discovering new drug targets that can control the disease-causing asexual blood stage growth phase of the parasite life cycle

Health Poverty Action is a UK based international NGO founded in 1984, originally under the name of Health Unlimited. Our mission is to strengthen poor and marginalized people in their struggle for health. We specialize in communicable disease and maternal health programs in underserved communities and through cross-border programs. HPA views health as an issue of social justice, recognizing that the greatest causes of poor health worldwide are political, social and economic injustices, and we prioritize those missed out by others, making neglected and marginalized populations our highest priority, applying culturally-appropriate methodologies that meet the specific needs of indigenous people (IP), ethnic minorities, migrants and other mobile and marginalized groups. We build strong, lasting relationships in these communities and forge effective collaborations between health staff and communities across national borders. HPA currently implements programs in 15 countries in Asia, Africa and Latin America. Past and present areas of intervention include: malaria, HIV/AIDS, TB, maternal and child health, health system strengthening, health education/BCC, food security and nutrition, water and sanitation, and gender based violence. Most of programs have focused on indigenous populations/ethnic minorities, particularly those in Cambodia, Laos and Viet Nam.

HPA has successfully implemented over $49 million in GF resources (as an SR or SSR) for programs in 10 different countries since 2004. HPA has operated in Cambodia since 1990, in Laos and Myanmar since 1994, working with remote IPs and MMPs. HPAs work in Viet Nam commenced in 2015, in the central highlands through a cross-border initiative. HPA currently implements malaria activities on all sides of the borders between Cambodia, Laos and Viet Nam as well as in Myanmar. All of these interventions have targeted IPs, MMPs, and/or FGs. In addition, HPA has successfully implemented cross-border programs between Myanmar/China and Cambodia/ Laos/Viet Nam, establishing new channels of communication and technical cooperation between countries, even in the face of political tensions related to military border disputes.

HPA has implemented the RAI2E Regional Component (2018-2020) in north eastern Cambodia, Southern Laos and Western Vietnam since January 2018, managing 3 country offices and 4 SRs to cover all the listed activities in the package. During Jan-Dec 2019 alone, this involved 18,037 LLINs distributed (continuous, +70,246 mass), training 973

VHWs/MMWs and setting up 137 MP/MP/MOTs to conduct 88,283 tests amongst MMP/EMs/Forest Goers, providing first line treatment to 740 patients and 7 referrals. During the same period, HPA conducted 175 supportive supervisions of VHWs/MPs/MOTs and widespread IEC/SBCC activities covering a total targeted population of 385,204. HPA's PM&E and its partnership with UCSF continue to build evidence surveillance capacity, mapping MMEV venues and assessing treatment-seeking behaviours.

Beyond this, in the RAI3E key areas in Cambodia, Laos and Myanmar, HPA has administered RAI2E ICC (2018-2020) grants and a malaria reduction grant from Comic Relief (2017-2020) in Cambodia and Laos. We worked extensively with VMWs to build capacity, and specifically in Comic Relief, to conduct IEC/SBCC activities through radio shows, videos, events on malaria, dengue and Covid19. In Myanmar, HPA has distributed 567,851 LLINs during 2014-2019 in the special regions in Kachin and Shan state. Under HPA's RAI2E grant in Myanmar, HPA is supporting 554 ICMV to provide free malaria diagnosis and treatment by end of 2019.

Under the current GFATM grant round, HPA is working on the following malaria projects in the region:

- RAI3E Regional Component: "Extending access to Malaria Services among migrant, mobile, ethnic and vulnerable populations" in Laos and Vietnam, 2021-2023
- RAI3E Country Component: "Regional Artemisinin-resistance Initiative 3 Elimination (RAI3E)", Laos, 2021-2023
- RAI3E Country Component: "Regional Artemisinin-resistance Initiative 3 Elimination (RAI3E)", Vietnam, 2021-2023
- COVID-19 Response Mechanism: "Improve response capacity to mitigate COVID-19 impact on HIV, TB and Malaria programmes", Vietnam, 2021-2023
- RAI3E Regional Operational Research (OR): 1-3-7 Reactive Surveillance and Response, GMS, 2021-2022
- RAI3E Regional Operational Research (OR): Forest-Goer Vector Control, 2021 2022

References

1. World Health Organization: **World malaria report 2020: 20 years of global progress and challenges**. In. Geneva: World Health Organization; 2020.
2. World Health Organization: **Guidelines for the treatment of malaria**. In., Third edition edn. Italy: World Health Organisation,; 2015.
3. World Health Organization: **Global technical strategy for malaria 2016-2030**. In. Geneva; 2015.
4. World Health Organization: **A framework for malaria elimination**. In. Edited by World Health Organization. Geneva: World Health Organization,; 2017.
5. Cui L, Yan G, Sattabongkot J, Cao Y, Chen B, Chen X, Fan Q, Fang Q, Jongwutiwes S, Parker D *et al*: **Malaria in the Greater Mekong Subregion: Heterogeneity and complexity**. *Acta tropica* 2012, **121**(3):227-239.
6. Dondorp AM, Nosten F, Yi P, Das D, Phyo AP, Tarning J, Lwin KM, Ariey F, Hanpithakpong W, Lee SJ *et al*: **Artemisinin resistance in Plasmodium falciparum malaria**. *N Engl J Med* 2009, **361**(5):455-467.
7. Ashley EA, Dhorda M, Fairhurst RM, Amaratunga C, Lim P, Suon S, Sreng S, Anderson JM, Mao S, Sam B *et al*: **Spread of artemisinin resistance in Plasmodium falciparum malaria**. *N Engl J Med* 2014, **371**(5):411-423.
8. Takala-Harrison S, Jacob CG, Arze C, Cummings MP, Silva JC, Dondorp AM, Fukuda MM, Hien TT, Mayxay M, Noedl H *et al*: **Independent emergence of artemisinin resistance mutations among Plasmodium falciparum in Southeast Asia**. *J Infect Dis* 2015, **211**(5):670-679.
9. Ariey F, Witkowski B, Amaratunga C, Beghain J, Langlois AC, Khim N, Kim S, Duru V, Bouchier C, Ma L *et al*: **A molecular marker of artemisinin-resistant Plasmodium falciparum malaria**. *Nature* 2014, **505**(7481):50-55.
10. World Health Organization: **The Mekong Malaria Elimination Programme: Countries of the Greater Mekong ready for the "last mile" of malaria elimination**. In.: World Health Organization; 2020.
11. World Health Organization: **Strategy for Malaria Elimination in the Greater Mekong Subregion (2015–2030)**. In.; 2015.
12. Canavati SE, Kelly GC, Quintero CE, Vo TH, Tran LK, Ngo TD, Tran DT, Edgel KA, Martin NJ: **Targeting high risk forest goers for malaria elimination: a novel approach for investigating forest malaria to inform program intervention in Vietnam**. *BMC infectious diseases* 2020, **20**(1):757.
13. Parker DM, Landier J, von Seidlein L, Dondorp A, White L, Hanboonkunupakarn B, Maude RJ, Nosten FH: **Limitations of malaria reactive case detection in an area of low and unstable transmission on the Myanmar-Thailand border**. *Malar J* 2016, **15**(1):571.

14. Guyant P, Canavati SE, Chea N, Ly P, Whittaker MA, Roca-Feltrer A, Yeung S: **Malaria and the mobile and migrant population in Cambodia: a population movement framework to inform strategies for malaria control and elimination**. *Malar J* 2015, **14**:252.
15. World Health Organization: **Malaria Surveillance, Monitoring & Evaluation: A reference manual**. In., Licence: CC BY-NC-SA 3.0 IGO. edn. Geneva; 2018.
16. World Health Organization: **Malaria surveillance, monitoring & evaluation: a reference manual**. Genève: World Health Organization; 2018.
17. Organization WH: **Disease Surveillance for Malaria Elimination**. 2012.
18. Sturrock HJ, Hsiang MS, Cohen JM, Smith DL, Greenhouse B, Bousema T, Gosling RD: **Targeting asymptomatic malaria infections: active surveillance in control and elimination**. *PLoS Med* 2013, **10**(6):e1001467.
19. Moonen B, Cohen JM, Snow RW, Slutsker L, Drakeley C, Smith DL, Abeyasinghe RR, Rodriguez MH, Maharaj R, Tanner M *et al*: **Operational strategies to achieve and maintain malaria elimination**. *Lancet* 2010, **376**(9752):1592-1603.
20. Wang D, Cotter C, Sun X, Bennett A, Gosling RD, Xiao N: **Adapting the local response for malaria elimination through evaluation of the 1-3-7 system performance in the China–Myanmar border region**. *Malaria Journal* 2017, **16**(1):54.
21. Smith Gueye C, Sanders KC, Galappaththy GNL, Rundi C, Tobgay T, Sovannaroth S, Gao Q, Surya A, Thakur GD, Baquilod M *et al*: **Active case detection for malaria elimination: a survey among Asia Pacific countries**. *Malaria Journal* 2013, **12**(1):358.
22. Deen J, Mukaka M, von Seidlein L: **What is the yield of malaria reactive case detection in the Greater Mekong Sub-region? A review of published data and meta-analysis**. *Malaria Journal* 2021, **20**(1):131.
23. Page MJ, McKenzie JE, Bossuyt PM, Boutron I, Hoffmann TC, Mulrow CD, Shamseer L, Tetzlaff JM, Akl EA, Brennan SE *et al*: **The PRISMA 2020 statement: an updated guideline for reporting systematic reviews**. *BMJ* 2021, **372**:n71.
24. Gething PW, Patil AP, Smith DL, Guerra CA, Elyazar IR, Johnston GL, Tatem AJ, Hay SI: **A new world malaria map: Plasmodium falciparum endemicity in 2010**. *Malaria Journal* 2011, **10**(1):1-16.
25. Balshem H, Helfand M, Schünemann HJ, Oxman AD, Kunz R, Brozek J, Vist GE, Falck-Ytter Y, Meerpohl J, Norris S *et al*: **GRADE guidelines: 3. Rating the quality of evidence**. *Journal of clinical epidemiology* 2011, **64**(4):401-406.
26. Lewin S, Glenton C, Munthe-Kaas H, Carlsen B, Colvin CJ, Gülmezoglu M, Noyes J, Booth A, Garside R, Rashidian A: **Using Qualitative Evidence in Decision Making for Health and Social Interventions: An Approach to**

Assess Confidence in Findings from Qualitative Evidence Syntheses (GRADE-CERQual). *PLOS Medicine* 2015, **12**(10):e1001895.

27. Ministry of Health MaIOfM: **Guidelines on the prevention and control of malaria for migrants in Myanmar**. In. Yangon: International Organization for Migration; 2012.

13. Appendices

Appendix A. Outcomes

Appendix B. Participant selection tools for Part B: Questionnaire surveys

Screening tool for states/regions/provinces eligible for Questionnaire 1 of the study

Screening tool for malaria program stakeholders eligible for Questionnaire 1 of the study

Screening tool for states/regions/provinces eligible for Questionnaire 2 of the study

Screening tool for frontline malaria service providers eligible for Questionnaire 2 of the study

Appendix C. Questionnaires for surveying the malaria program stakeholders

Questionnaire 1 for surveying the malaria program stakeholders responsible for managing or supervising field reactive surveillance and response activities

Questionnaire 2 for surveying frontline malaria service providers

Appendix D. Topic guides for semi-structured interviews and focus group discussions

Topic guide for semi-structured interview with malaria program stakeholders responsible for designing and overviewing malaria reactive surveillance and response policies and strategies

Topic guide for focus group discussion with malaria program stakeholders responsible for managing or supervising field reactive surveillance and response activities

Topic guide for focus group discussion with frontline malaria service providers

Topic guide for focus group discussion with mobile and migrant populations

Appendix E. List of variables to be collected from the national malaria control programs for secondar data analysis (Part D)

Appendix F. Data transfer agreement

Appendix G. Participant information and consent forms

Participant information and consent form for questionnaire survey of the malaria program stakeholders responsible for managing or supervising field reactive surveillance and response activities

Participant information and consent form for questionnaire survey of frontline malaria service providers

Participant information and consent form for semi-structured interview with malaria program stakeholders responsible for designing and overviewing malaria reactive surveillance and response policies and strategies

Participant information and consent form for focus group discussion with malaria program stakeholders responsible for managing or supervising field reactive surveillance and response activities

Participant information and consent form for focus group discussion with frontline malaria service providers

Participant information and consent form for focus group discussion with mobile and migrant populations

Appendix H. Outline of data flow

Appendix I. Approved budget
